# Flash Communication: Intramolecular Chalcogen Bonding as a Driver of Diaryltelluride Oxidation by *o*‑Chloranil

**DOI:** 10.1021/acs.organomet.6c00178

**Published:** 2026-07-17

**Authors:** You Jiang, Chenggang Jiang, François P. Gabbaï

**Affiliations:** Department of Chemistry, 14736 Texas A&M University, College Station, Texas 77843-3255, United States

## Abstract

Given potential ramifications in catalysis, exploring redox cycling at heavy main group centers is attracting renewed interest. In this context, this study explores how chalcogen bonding interactions can influence the oxidative addition of *o*-chloranil to the Te­(II) center of diaryltellurides. Using a series of diaryltellurides functionalized by *ortho*-methylene-dimethylamino or -methyl ether functionalities, we show that the formation of a chalcogen bond between the donor atom and the tellurium center drives the oxidation reaction, as confirmed by the isolation and structural characterization of the corresponding Te­(IV) monocatecholates. Furthermore, this study also shows that these tetravalent tellurium derivatives react with an additional equivalent of *o*-chloranil to afford hexachloro-dibenzo­[1,4]­dioxine-2,3-dione, a known compound, and the corresponding Te­(IV) dichlorides. Lastly, this work also documents the facile reductions of the Te­(IV) dichlorides into their Te­(II) diaryl precursors using dithiothreitol or DTT.

## Graphical abstract

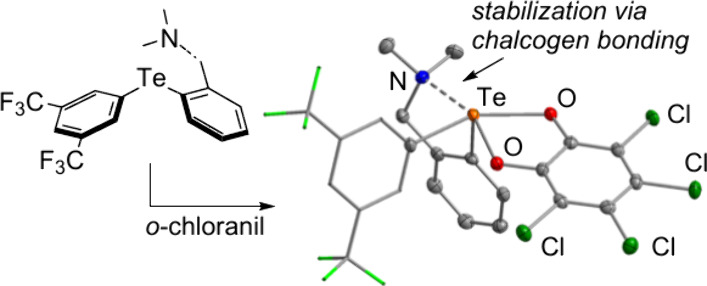

The use of main group elements as platforms for two-electron redox catalysis is an active area of research that has witnessed significant recent advances.^1–5^ In this general context, understanding the elementary steps that govern the oxidative addition and reductive elimination steps in main group chemistry is a worthwhile objective. An interesting case is presented by diaryltellurides, which have seen some use in redox catalysis.^6–9^ Such species are readily oxidized by halogens or halogen-like reagents; yet they appear to resist the addition of *ortho*-quinones. This fact may appear surprising given that bis­(catecholate) Te­(IV) complexes of type **I** (Figure 1), can be obtained by mixing *ortho*-quinones with elemental tellurium under heating or from catechols and TeO_2_.^10–14^ Diorganyl tellurium catecholates are scarce and have mostly been documented in systems featuring tellurium-bound alkyl groups (**II**, Figure 1),^10,15^ reinforcing our contention concerning the inert character of diaryltellurides toward *ortho*-quinones. Along this line, we note that confirmed diaryltellurium catecholates are rather elusive,^15^ suggesting that such species may be inherently unstable or prone to elimination of the corresponding *ortho*-quinone.

**Figure 1:**
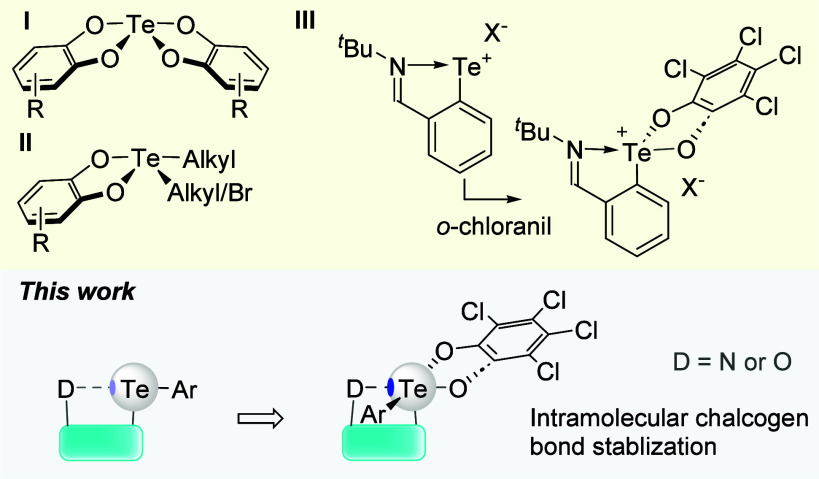
Previously reported tellurium based catecholates and design of such compounds assisted by intramolecular chalcogen bonding.

Bearing in mind that oxidative addition to reluctant main-group centers can be assisted and thus promoted by coordination of a Lewis base,^16,17^ we questioned whether a strategy based on intramolecular Lewis base stabilization or chalcogen bonding,^18–22^ would also provide access to simple diaryl tellurium catecholates. As this work was in progress, our contention was reinforced by an elegant account by Hejda who showed that this strategy is effective to access cationic aryl tellurium catecholates as in the case of **III**,^23^ obtained by reaction of the tellurenyl species with 3,4,5,6-tetrachloro-1,2-benzoquinone (*o*-chloranil), as shown in Figure 1.

To begin our investigation, we evaluated the reactivity of simple diphenyl telluride (Ph_2_Te) toward *o*-chloranil to access Te­(IV) monocatecholate species. As shown by ^1^H NMR (), two broad peaks formed, along with the resonances of Ph_2_Te, indicating a possible equilibrium between the Te­(II) and Te­(IV) species. Efforts to isolate the Te­(IV) compound from this mixture did not bear fruit, including upon addition of exogenous bases such as Et_3_N and Ph_3_PO (). Returning to our original contention that intramolecular Lewis base coordination may alter the fate of such reactions, we considered the donor-substituted diaryltellurides **1**–**3**. While compound **2** is known in the literature,^24^ compounds **1** and **3** were prepared by reaction of donor-functionalized aryl lithium reagents with *in situ* generated ArTeBr, followed by purification by column chromatography (see ). Treatment of compounds **1**–**3** with one equivalent of *o*-chloranil in CH_2_Cl_2_ under ambient conditions led to the clean formation of the new derivatives **4**–**6** (Figure 2). These derivatives, obtained yellow solids, are all stable to air and moisture. Their formation was readily apparent by ^1^H NMR spectroscopy. In all cases, the most diagnostic change is observed in the methylene region. For example, in precursor **1**, the methylene protons appear as a single resonance at 3.59 ppm (), whereas in compounds **4**, this moiety gives rise to an AB system at 3.62 ppm (), consistent with the diastereotopic relationship of the two methylene protons, induced by coordination of the nitrogen to a chiral tellurium center. The same changes are observed in compounds **5** and **6**, with splitting of the methylene peak into two doublets (). Such changes are well documented in the literature,^24–26^ for example during the conversion of **2** into the corresponding telluroxide.^24^

**Figure 2:**
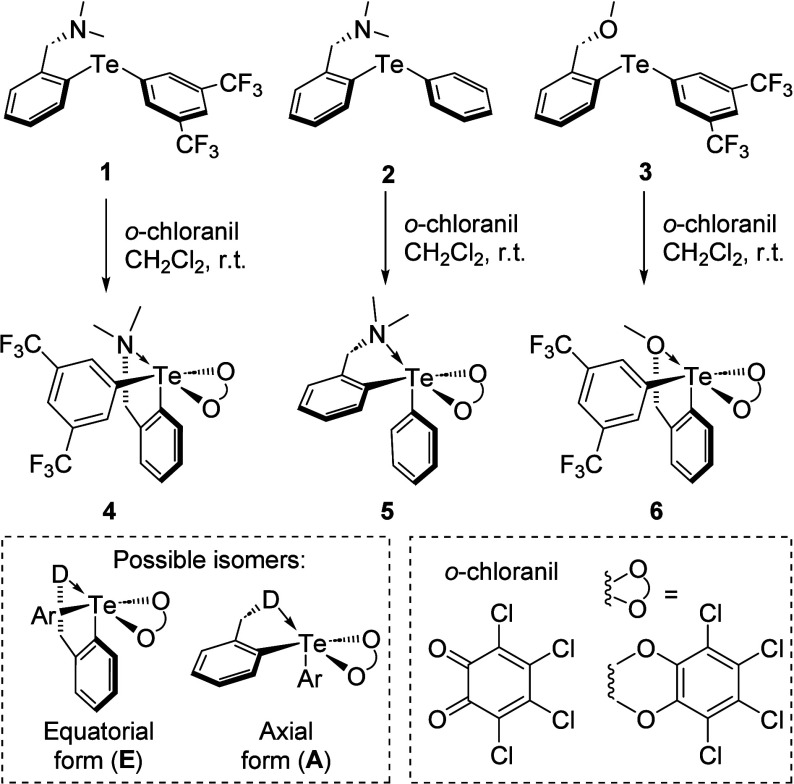
Synthesis of compounds **4**-**6**.

The oxidized compounds **4**-**6** were further characterized by single-crystal X-ray diffraction (sc-XRD), which unambiguously confirms the formation of diaryl Te­(IV) monocatecholate species (Figure 3). In all cases, the tellurium center adopts a distorted square pyramidal geometry, with the intramolecular donor atom coordinated to the Te center trans to one of the catecholate oxygen atoms. However, the conformations of the substituents differ. In compounds **4** and **6**, the electron-withdrawing 3,5-bis­(trifluoromethyl)­phenyl group (Ar^F^) adopts a planar arrangement, positioned trans and slightly offset from the axis of the other Te–O_cat_ bond (O_cat_ = catecholate oxygen atom). In contrast, the phenyl substituent in compound **5** aligns along the apical axis.^27^ The structural diversity displayed by these compounds suggests that the formation of one isomer vs. the other may not induce large energy differences. This view is supported by a computational survey, which shows very small energy differences between isomers **E** and **A** (Figure 2 and ), pointing to the possibility of lattice forces defining which isomer is observed in the crystal.^28^

**Figure 3:**
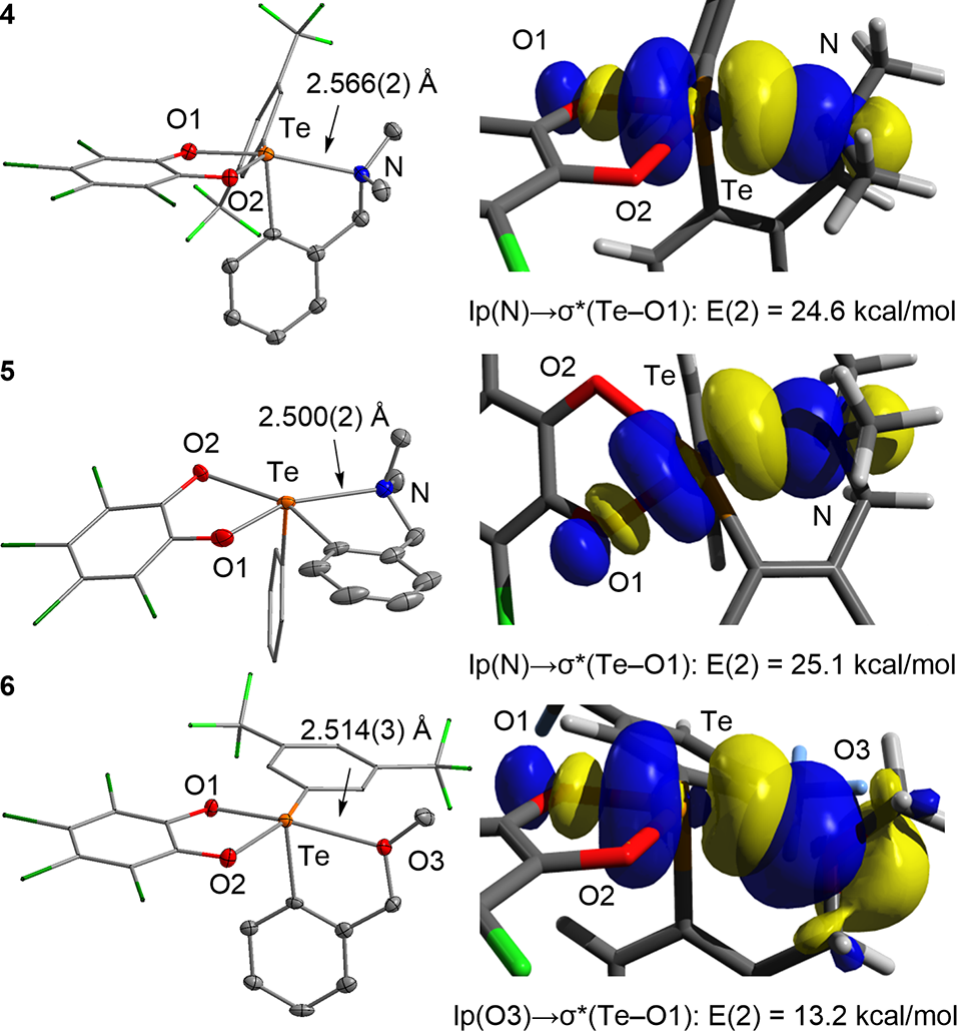
Left: Crystal structures of compounds **4**-**6**. Ellipsoids are drawn as 50% probability level. All the hydrogen atoms are omitted for clarity. Selected bond lengths (Å): **4**: Te–N, 2.566(2), Te–O1, 2.028(2), Te–O2, 2.284(2); **5**: Te–N, 2.500(2), Te–O1, 2.032(2), Te–O2, 2.446(2); **6**: Te–O1, 1.979(3), Te–O2, 2.299(3), Te–O3, 2.514(3). Right: The corresponding optimized structures with the main NBOs associated with the donor–Te interactions in each compound (isovalue = 0.05).

The intramolecular chalcogen bonding interactions present in these compounds are defined by Te–N bond distances of 2.566(2) Å and 2.500(2) Å for **4** and **5**, repectively and a Te–O3 distance of 2.514(3) Å in the case of **6**. These distances are significantly shorter than the sum of the van der Waals radii of the corresponding elements (Σ_vdw(Te–N)_ = 3.61 Å and Σ_vdw(Te–O)_ = 3.58 Å),^29^ consistent with significant intramolecular donor–acceptor bonding.^23,30,31^ Another notable feature in the structure of these compounds is the difference in length of the two Te–O_cat_ bonds connecting the catecholate moiety to the main group center. In each structure, the Te–O1 bond, corresponding to the oxygen atom trans to the donor atom, is close to 2.0 Å, consistent with the Te–O bond length in bis­(catecholate) Te­(IV) complexes.^10–13^ In contrast, the Te–O2 bond is significantly elongated reaching a maximum of 2.446(2) Å in **5**, a compound in which the lengthening may be accentuated by a hydrogen bonding interaction between the O2 atom and an interstitial CHCl_3_ molecule. The observed difference of the Te–O_cat_ bonds in **4**-**6** contrasts with the more symmetrical coordination of the catecholate ligand in compounds of types **I**, **II** and **III**,^10–13,23^ leading us to question whether a semiquinone resonance form should also be considered. To better assess this possibility, we use the metrical oxidation state (MOS) method introduced by Brown to assess the degree of reduction of the catecholate.^32^ The calculated MOS values for **4**-**6** range from −1.68 to −1.87 showing substantial reduction of the *ortho*-quinone (). The magnitude of these values suggest that the ligands are essentially in their dianionic form rather than their semiquinone form.^33^ This argument is also supported by the diamagnetic nature of compounds **4**-**6** ().

These tellurium-based monocatecholates were also computationally investigated. The optimized structures revealed Te-O distances of 2.56 Å and 2.54 Å for **4** and **5**, respectively, **4**, 2.54 Å for compound **5**and a Te-O distance of 2.60 Å for **6**. These distances are in good agreement with those experimentally determined, supporting the suitability of the chosen method. A Natural Bond Orbital (NBO) analysis was next carried out to investigate the intramolecular interactions (Figure 3). A lp­(donor) → σ*­(Te–O) donor–acceptor interaction is found in all three catecholates, consistent with the presence of chalcogen bonding. In compounds **4** and **5**, the strengths of the interactions are comparable, contributing 24.6 and 25.1 kcal/mol, respectively, to the stability of the molecules as derived from the corresponding second-order energy correction term *E* ^(2)^. The similarity of the strength of these interactions indicates that the nature of the substituents and their different electronic properties does not play a significant role on the charge transfer term of the intramolecular chalcogen bond in these compounds. In contrast, compound **6** exhibits a significantly lower *E* ^(2)^ at 13.2 kcal/mol, due to the weaker donating ability of ethers compared to amines,^34^ leading to a diminished chalcogen bonding interaction. The Wiberg bond indices (WBIs) calculated for the Te–O1 bond (1.04, 1.04, and 1.15 for compounds **4**-**6**, respectively) and Te–O2 bond (0.59, 0.51, and 0.64 for compounds **4**-**6**, respectively) are consistent with the observed asymmetry of the Te–O_cat_ bond lengths. The low WBI found for the Te–O2 bond suggests that these compounds may be reactive, a notion supported by further results obtained in this study (*vide infra*).

Building on these synthetic results, we decided to computationally investigate the thermochemistry of the formation of the tellurium-based monocatecholates from *o*-chloranil and the corresponding diaryltelluride. The results of these calculations are presented in Table 1. These calculations yielded negative free-energy values for all three Lewis-base-containing systems at 298 K, indicating that the formation of the monocatecholate species is thermodynamically favorable. It is interesting to note that the formation of **6** is less exergonic than that of its nitrogen-containing counterparts, a result that can be correlated to the weaker chalcogen bonding interaction in the ether vs. amine derivatives.^34^ To further test the influence of chalcogen bonding as a driver of the tellurium oxidation reaction, we also included in our computational survey the reaction of Ph_2_Te and *o*-chloranil for which a positive free energy change of 1.2 kcal/mol was obtained.^34^ This result is consistent with our experimental observations (*vide supra*) and highlights the crucial role of intramolecular chalcogen bonding in promoting the reaction *via* stabilization of the tellurium monocatecholates.

**Table 1:**
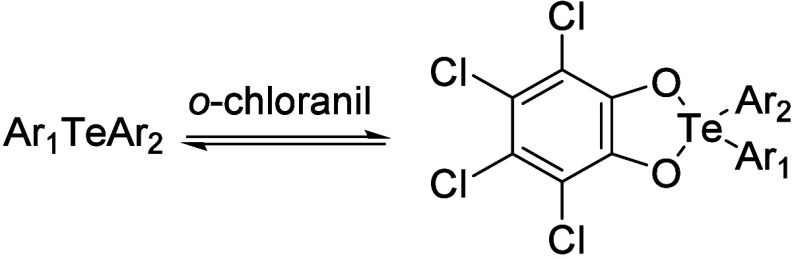
Computed Thermodynamic Parameters for the Reaction of Ph_2_Te and **1**-**3** with *o*-Chloranil

Compounds	ΔH (kcal/mol)	ΔG (kcal/mol)
Ph_2_Te	–14.1	1.2
**1**	–22.2	–5.1
**2**	–24.2	–8.2
**3**	–16.8	–1.9

We noticed that the outcome of the reaction leading to **4**-**6** may be influenced by the presence of excess *ortho*-quinones. To better assess this possibility, compounds **4**–**6** were treated with an excess of *o*-chloranil (Figure 4), resulting in a quick transformation, as evidenced by significant changes in the corresponding ^1^H NMR spectra (). Particularly diagnostic are changes in the methylene proton resonances that lose their multiplicity to collapse into a singlet, indicating complete consumption of the Te­(IV) monocatecholate species. To better control the reaction, compounds **4**–**6** were treated with only one equivalent of *o*-chloranil under ambient conditions in CH_2_Cl_2_ for 24 h. The reaction was accompanied by the progressive formation of a red precipitate, which we unambiguously identified as the known hexachloro-dibenzo­[1,4]­dioxine-2,3-dione (**7**), as confirmed by X-ray analysis (Figure 4) and ^13^C NMR spectroscopy (). This product has been previously observed in reactions involving *o*-chloranil with metal ions or Et_3_SiH.^23,35–37^ In addition, the reaction can also be conveniently carried out by combining two equivalents of *o*-chloranil with **1**–**3,** indicating that isolation of the Te­(IV) monocatecholate intermediates (**4**-**6**) is not a requirement.

**Figure 4:**
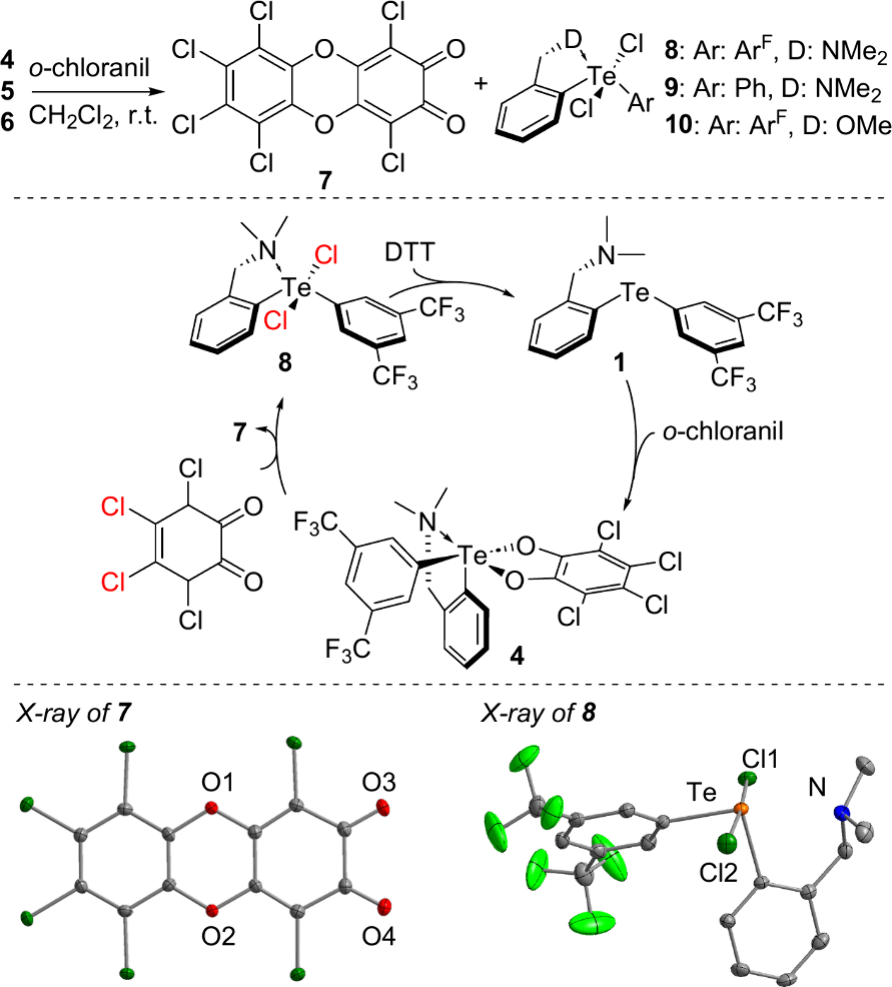
Top: Reactions between the tellurium-based catecholate compounds **4**-**6** with one more equivalent of *o*-chloranil. Middle: Synthetic cycle showing the reduction of dichloride **8** with DTT. Bottom: Structures of **7** and **8** in the crystal. Selected bond lengths for compound **8** (Å): Te–N: 2.722(2), Te–Cl1:2.5038(7), Te–Cl2:2.5002(7).

Analysis of the reaction mixture by mass spectrometry () revealed the concomitant formation of the diaryltellurium dichlorides **8**–**10** (Figure 4). To verify the assignment, compounds **8**–**10** were independently synthesized by oxidation of the parent diaryltellurides **1**–**3** with sulfuryl chloride. The ^1^H NMR spectra of compounds **8**–**10** matched those obtained from analysis of the reaction mixtures (), confirming their formation in the reactions of **4**–**6** with *o*-chloranil.

Further evidence for the formation of these dichlorides was obtained by X-ray analysis of single crystals of compound **8**, grown from the reaction mixture. The structure of **8** is shown in Figure 4. With apicophilicity at play, the two chloride ligands are trans from each other, leaving the amino nitrogen atom trans from the Ar^F^ substituent. The resulting N–Te distance of 2.722(2) Å is longer than in compound **4** (2.566(2) Å), pointing to the favorable role of the tetrachlorocatecholate ligand in predisposing the tellurium center in a more Lewis acidic state. Similar effects have been observed in antimony­(V) catecholates, which are typically better pnictogen-bond donors than their dihalide counterparts.^38–42^ To close the synthetic cycle, we have also investigated the reduction **8** back into its Te­(II) precursor **1**. In agreement with recent results from our group,^31^ we found that dithiothreitol (DTT) is an appropriate reagent for this transformation, which was also carried out successfully for the reduction of **9** and **10** (). Because DTT reduces *o*-chloranil, running this chemistry catalytically has not been realized.^39–41^ The reduction may also be carried out with other reductants, as in the case of **10** which is readily reduced back to **3** by glutathione or Na_2_SO_3_ but which remained untouched in the presence of Hantzsch ester ().

Finally, we found that when **1** is combined with two equivalents of *o*-chloranil in Et_2_O rather than DCM, a new Te­(IV) species (**11**) featuring a polycyclic ligand derived from the condensation of three *o*-chloranil units is obtained (Figure 5). This compound has been identified solely based on X-ray analysis carried out on crystals that grew on the wall of the vial when the reaction mixture was left to stand at room temperature. While we do not know the mechanism leading to this product, its formation must involve the reduction of three *o*-chloranil units prior to condensation. The presence of a terminal oxygen atom also points to the involvement of adventitious water.

**Figure 5:**
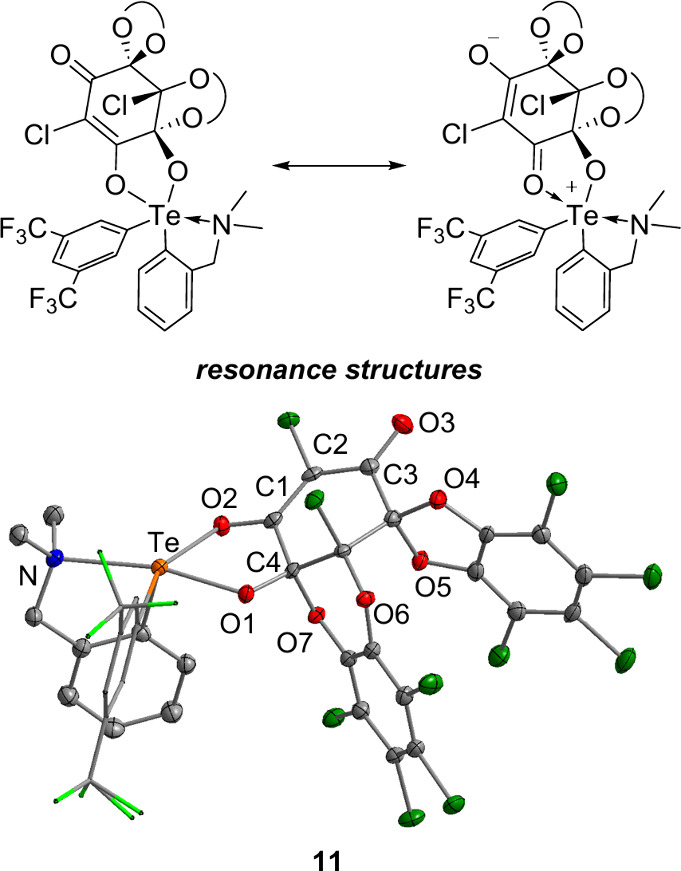
Resonance forms of **11** and its structure in the crystal. Selected bond lengths (Å): Te–N: 2.405(4), Te–O1:2.061(3), Te–O2:2.494(3), O1–C4:1.365(5), O2–C1:1.251(5), C1–C2:1.386(6), C2–C3:1.423(6), C3–O3:1.226(5).

In summary, this study shows that the presence of an adjacent donor functionality can favor oxidative addition of *o*-chloranil to the otherwise reluctant Te­(II) center of diaryltellurides. The exergonic character benefits from a favorable energy term arising from the stability of the electron donor interaction and the Lewis acidic tellurium center. The dative interactions found in these compounds are however quite weak, making their descriptions as chalcogen bonds appropriate.^18^ Another important outcome of this study concerns the reactivity of these compounds and the observed formation of dioxin **7**, which is readily promoted by these systems.

## Supplementary Material





## Notes

Synthetic details; recorded NMR spectra; X-ray diffraction details; computational details, thermochemical analyses in PDF format (PDF)
Atomic coordinates for the optimized structures in XYZ format (XYZ)
