# Supplementary material for: Flash Communication: Intramolecular Chalcogen Bonding as a Driver of Diaryltelluride Oxidation by o‑Chloranil
Source: Organometallics. 2026 Jul 17;45(15):1764–9. doi: 10.1021/acs.organomet.6c00178 (PMC13463677; doi:10.1021/acs.organomet.6c00178)
Supplement: Supplementary file 1 [file om6c00178_si_001.pdf]

Flash Communication : Intramolecular chalcogen bonding as a driver of diaryltelluride oxidation by *o*-chloranil

You Jiang, Chenggang Jiang, and François P. Gabbaï*

Department of Chemistry, Texas A&M University, College Station, TX 77843-3255, United States of America

email: francois@tamu.edu

Supporting Information

Contents

1 Synthesis and Characterization ... S2
1.1 General Considerations ... S2
1.2 Synthetic Procedures ... S2
1.3 NMR Spectra ... S6
2 Reaction of Diphenyl Telluriude and *o*-Chloranil ... S21
3 Metrical Oxidation State (MOS) of the Catecholate ... S23
4 Reaction of Diaryl Tellurium Compounds and *o*-Chloranil ... S24
4.1 NMR Spectra ... S24
4.2 Mass Spectra ... S26
4.3 Large Scale Reaction of Diaryl Tellurium(II) Compounds and *o*-Chloranil .. S27
5 Reduction of diaryl tellurium dichlorides ... S29
6 X-ray Crystallographic Data ... S33
7 Computational Methods ... S34
7.1 Geometry optimization and energy calculations ... S34
7.2 Comparison of Isomer Energies ... S34
7.3 Energy Summary ... S35
References ... S36

1 Synthesis and Characterization

1.1 General Considerations

All experiments were carried out in an ambient environment unless otherwise noted. Chemicals were purchased from commercial suppliers and used without further purification. Diphenyl telluride, bis(3,5-bis(trifluoromethyl)phenyl) ditelluride and compound **2** were prepared according to literature procedures.[1-3] Diethyl ether (Et_2O) was dried by refluxing over Na and tetrahydrofuran (THF) over Na/K. All other solvents were ACS reagent grade and used as received. ^{1}H NMR and $^{13}C\{^{1}H\}$ NMR spectra were recorded at room temperature using a Bruker Avance 500 NMR spectrometer, or a Bruker Ascend 400 NMR spectrometer. ^{19}F and $^{125}Te\{^{1}H\}$ NMR spectra were recorded at room temperature using a Bruker Ascend 400 NMR spectrometer. Chemical shifts are given in ppm. ^{1}H and ^{13}C signals were referenced to residual ^{1}H or ^{13}C solvent signals. Elemental analyses were performed by Atlantic Microlab (Norcross, GA).

1.2 Synthetic Procedures

Synthesis of compound 1

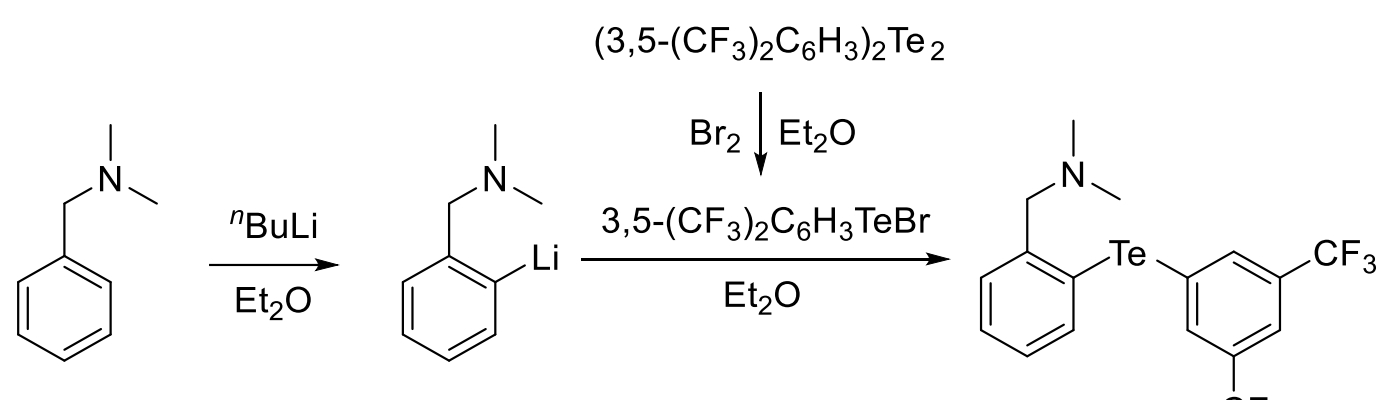

Under a nitrogen atmosphere, ^{n}BuLi (10 mL, 16.1 mmol, 2.2 equiv., 1.6 M in hexanes) was added dropwise to a solution of *N,N*-dimethylbenzylamine (2.1 g, 15.4 mmol, 2.1 equiv.) in anhydrous Et_2O (20 mL) at room temperature to afford a solution that was stirred for 6 h. In a separate Schlenk flask, bromine (0.38 mL, 7.3 mmol, 1.0 equiv.) was added to a solution of bis(3,5-bis(trifluoromethyl)phenyl) ditelluride (5.0 g, 7.3 mmol, 1.0 equiv.) in Et_2O (20 mL). The resulting mixture was stirred for 1 h to afford $(CF_3)_2C_6H_3TeBr$, which was slowly transferred to the organolithium solution. The combined mixture was stirred overnight and then quenched with saturated aqueous NH_4Cl (80 mL). The aqueous layer was extracted with Et_2O (3 × 50 mL). The combined organic phases were dried over $MgSO_4$, filtered, and brought to dryness under vacuum to afford a dark red residue. The crude product was purified by 100–200 mesh silica gel flash chromatography (hexanes/CH_2Cl_2 = 4:1 as the eluent) to give compound **1** as a brown solid (2.1 g, 4.4 mmol, 60% yield). **^{1}H NMR** (500 MHz, CD_2Cl_2) δ 8.30 (s, 2H, Ar-*H*), 7.82 (s, 1H, Ar-*H*), 7.20–7.09 (m, 2H, Ar-*H*), 7.09–7.03 (m, 1H, Ar-*H*), 7.01–6.93 (m, 1H, Ar-*H*), 3.59 (s, 2H, C*H₂*), 2.30 (s, 6H, CH_3). **^{13}C{^{1}H} NMR** (126 MHz, CD_2Cl_2) δ 140.5, 139.7 (d, *J* = 3.9 Hz), 134.3, 131.6 (q, *J* = 32.9 Hz), 128.7, 128.3, 126.5, 125.0, 123.2 (q, *J* = 273.0 Hz), 121.8, 121.6 (m), 65.8, 43.6. **^{19}F NMR** (470 MHz, CD_2Cl_2) δ -62.76 (s). **^{125}Te{^{1}H} NMR** (126 MHz,

CD_2Cl_2) δ 675.4 (s). **Elemental Analysis** $C_{17}H_{15}F_6NTe$ calculated: C: 43.00; H: 3.18. Found: C: 42.91; H: 3.07.

Synthesis of compound 3

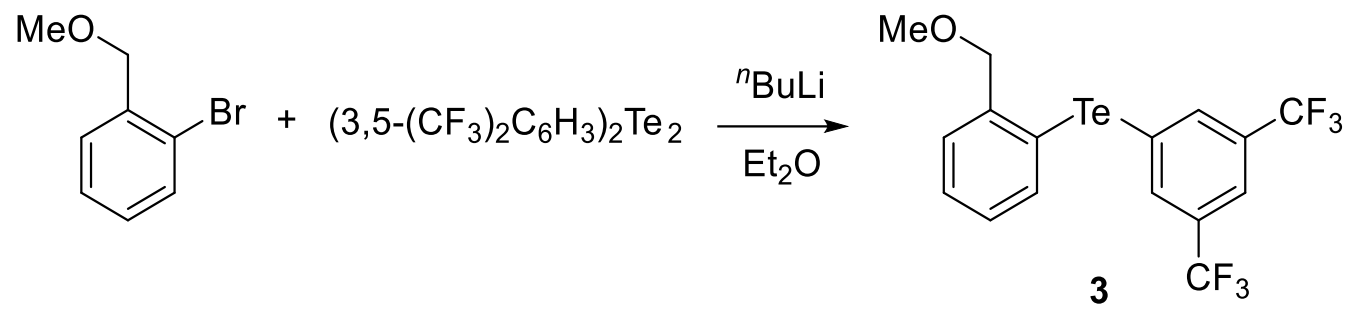

3

Under a nitrogen atmosphere, ^{n}BuLi (1.0 mL, 2.5 mmol, 1.1 equiv., 2.5 M in hexanes) was added dropwise to a solution of 1-bromo-2-(methoxymethyl)benzene (0.5 g, 2.5 mmol, 1.1 equiv.) in anhydrous Et_2O (15 mL) cooled to -78 ℃. After stirring for 1 h at this temperature, the resulting solution was treated with bis(3,5-bis(trifluoromethyl)phenyl) ditelluride (1.54 g, 2.3 mmol, 1.0 equiv.) and then warmed up to room temperature gradually over 2 h. The resulting mixture was stirred overnight and then quenched with saturated aqueous NH_4Cl (30 mL). The aqueous phase was extracted with Et_2O (3 × 20 mL). The combined organic phases were dried over $MgSO_4$, filtered and brought to dryness under vacuum to afford a dark red residue. The crude product was purified by 100–200 mesh silica gel flash chromatography (hexanes/CH_2Cl_2 4:1 as the eluent) to give compound **3** as a yellow liquid (0.41 g, 0.9 mmol, 39% yield). **^{1}H NMR** (500 MHz, CD_2Cl_2) δ 8.20 (s, 2H, Ar-*H*), 7.82 (s, 1H, Ar-*H*), 7.38 (dd, *J* = 7.8, 1.3 Hz, 1H, Ar-*H*), 7.35 (dd, *J* = 7.6, 1.6 Hz, 1H, Ar-*H*), 7.27 (td, *J* = 7.5, 1.3 Hz, 1H, Ar-*H*), 4.54 (s, 2H, C*H₂*), 3.37 (s, 3H, C*H₃*). **^{13}C{^{1}H} NMR** (126 MHz, CD_2Cl_2) δ 141.0, 138.9 (d, *J* = 3.9 Hz), 136.9, 132.2 (q, *J* = 33.2 Hz), 129.5, 129.2, 128.1, 123.1 (q, *J* = 273.1 Hz), 122.1 (m), 119.1, 118.7, 77.6, 57.6. **^{19}F NMR** (470 MHz, CD_2Cl_2) δ -62.91 (s). **^{125}Te{^{1}H} NMR** (126 MHz, CD_2Cl_2) δ 671.0 (s). **Elemental Analysis** for $C_{16}H_{12}F_6OTe$, calculated: C: 41.61; H: 2.62. Found: C: 41.85; H: 2.51.

General procedure of synthesizing diaryl tellurium(IV) monocatecholate compounds

o-Chloranil (100 mg, 0.4 mmol, 1.0 equiv.) was added to a solution of the diaryl tellurium(II) compound (0.4 mmol, 1.0 equiv.) in CH_2Cl_2 (5 mL). The mixture was stirred for 5 min and then brought to dryness under vacuum. The crude product was recrystallized from hexanes to afford the corresponding diaryl tellurium(IV) monocatecholate product.

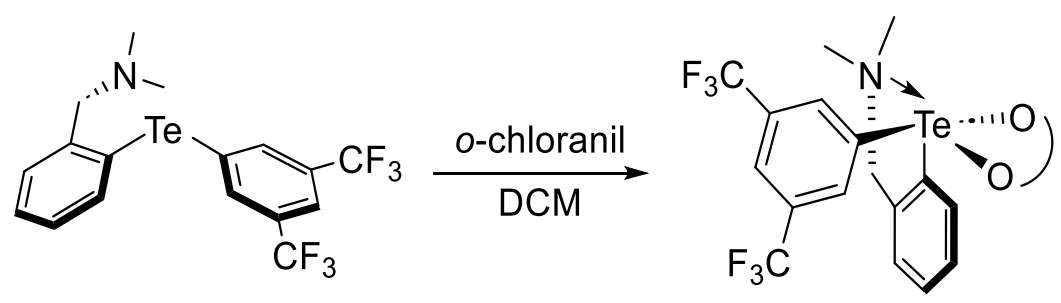

1 **4**

4: Yellow powder (255 mg, 0.35 mmol, 87% yield). **^{1}H NMR** (500 MHz, CD_2Cl_2) δ. 8.07–7.96 (m, 4H, Ar-*H*), 7.62 (m, 2H, Ar-*H*), 7.38 (d, *J* = 6.7 Hz, 1H, Ar-*H*), 3.88 (d, *J* = 14.9 Hz, 1H, C*H*₂), 3.36 (d, *J* = 14.9 Hz, 1H, C*H*₂), 2.65 (s, 3H, C*H₃*), 2.07 (s, 3H, C*H₃*). **^{13}C{^{1}H} NMR** (126 MHz, CD_2Cl_2) δ 148.5, 145.2, 140.4, 138.3, 133.6, 133.1 (d, *J* = 3.6 Hz), 132.8,

132.8 (q, *J* = 33.7 Hz), 131.2, 128.9, 125.6–125.4 (m), 123.5 (q, *J* = 273.3 Hz), 119.3, 117.5, 64.0, 45.6, 45.4. **^{19}F NMR** (470 MHz, CD_2Cl_2) δ -63.30 (s). **^{125}Te{^{1}H} NMR** (126 MHz, CD_2Cl_2) δ 1221.92 (s). **Elemental Analysis** $C_{23}H_{15}Cl_4F_6NO_2Te$ calculated: C: 38.33; H: 2.10. Found: C: 38.60; H: 2.05.

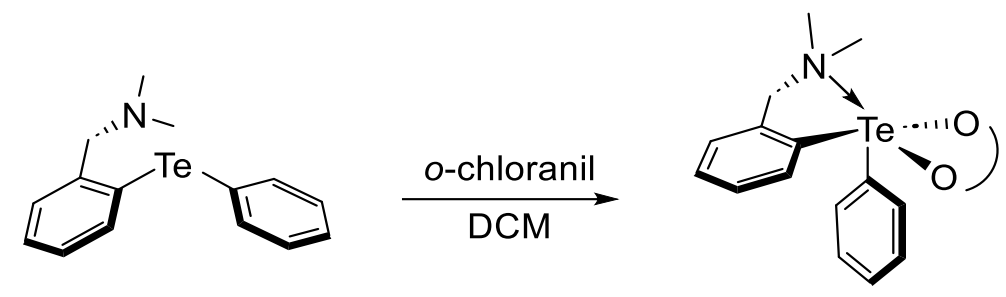

2 **5**

5: Yellow powder (201 mg, 0.34 mmol, 85% yield). **^{1}H NMR** (500 MHz, CD_2Cl_2) δ. 8.39 (d, *J* = 7.8 Hz, 1H, Ar-*H*), 7.67 – 7.62 (m, 3H, Ar-*H*), 7.56 (t, *J* = 7.6 Hz, 1H, Ar-*H*), 7.51–7.46 (m, 1H, Ar-*H*), 7.43 (t, *J* = 7.5 Hz, 2H, Ar-*H*), 7.34 (d, *J* = 7.5 Hz, 1H, Ar-*H*), 3.76 (d, *J* = 14.7 Hz, 1H, C*H₂*), 3.43 (d, *J* = 14.7 Hz, 1H, C*H₂*), 2.57 (s, 3H, C*H₃*), 1.97 (s, 3H, C*H₃*). **^{13}C{^{1}H} NMR** (126 MHz, CD_2Cl_2) δ 149.2, 141.9, 139.9, 138.4, 134.9, 133.0, 132.2, 131.9, 130.3, 130.2, 128.5, 118.2, 117.4, 64.7, 45.4, 45.2. **^{125}Te{^{1}H}** NMR (126 MHz, CD_2Cl_2) δ 1234.31 (s).

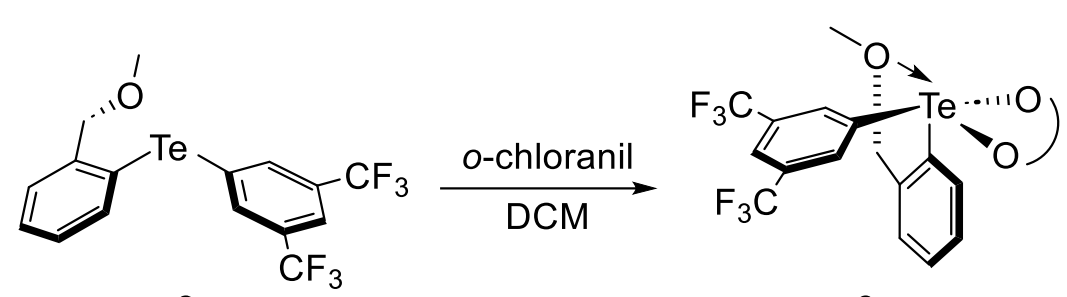

3 **6**

6: Yellow powder (230 mg, 0.32 mmol, 80% yield). **^{1}H NMR** (500 MHz, CD_2Cl_2) δ. 8.04 (s, 2H, Ar-*H*), 7.93 (s, 1H, Ar-*H*), 7.75 – 7.69 (m, 1H, Ar-*H*), 7.58 – 7.51 (m, 2H, Ar-*H*), 7.33 – 7.27 (m, 1H, Ar-*H*), 4.86 (d, *J* = 13.0 Hz, 1H, C*H₂*), 4.50 (d, *J* = 12.9 Hz, 1H, C*H₂*), 3.61 (s, 3H, C*H₃*). **^{13}C{^{1}H} NMR** (126 MHz, CD_2Cl_2) δ 149.2, 145.8, 138.2, 136.6, 132.0, 131.74, 131.67, 130.50, 130.48, 126.4, 124.2, 123.1 (q, *J* = 273.3 Hz), 120.0, 116.8, 74.3, 58.7. **^{19}F NMR** (470 MHz, CD_2Cl_2) δ = -63.30 (s). **^{125}Te{^{1}H} NMR** (126 MHz, CD_2Cl_2) δ 1284.42 (s). **Elemental Analysis** $C_{22}H_{12}Cl_4F_6O_3Te$ calculated: C: 37.34; H: 1.71. Found: C:37.60; H: 1.58.

Synthesis of diaryl tellurium(IV) dichloride compounds

SO_2Cl_2 (34 mg, 20 μL, 0.25 mmol, 2.0 equiv.) was added to a solution of corresponding diaryl tellurium compound (0.13 mmol, 1.0 equiv.) in CH_2Cl_2 (3 mL). The mixture was stirred for 5 min and then brought to dryness under vacuum. The crude product was washed with hexanes (3 × 5 mL) to afford the corresponding diaryl tellurium dichloride product.

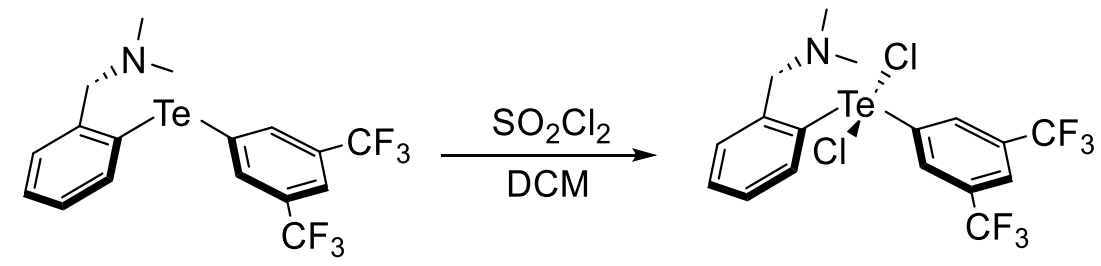

1 **8**

8: Yellow powder (64 mg, 0.12 mmol, 95% yield). **^{1}H NMR** (500 MHz, CD_2Cl_2) δ 8.77 (s, 2H, Ar-*H*), 8.06 (s, 1H, Ar-*H*), 7.51 (m, 1H, Ar-*H*), 7.39–7.30 (m, 3H, Ar-*H*), 4.11 (s, 2H, C*H₂*), 2.59 (s, 6H, C*H₃*). **^{13}C{^{1}H} NMR** (126 MHz, CD_2Cl_2) δ 142.9, 140.0, 139.1,

136.3 (d, J = 4.0 Hz), 134.2, 132.4 (q, J = 33.6 Hz), 132.2, 130.2, 129.9, 125.2–125.0 (m), 123.8 (q, J = 273.4 Hz), 62.3, 44.8. ^{19}**F NMR** (377 MHz, CD_2Cl_2) δ -63.04 (s). ^{125}**Te{^{1}H} NMR** (126 MHz, CD_2Cl_2) δ 956.3 (s).

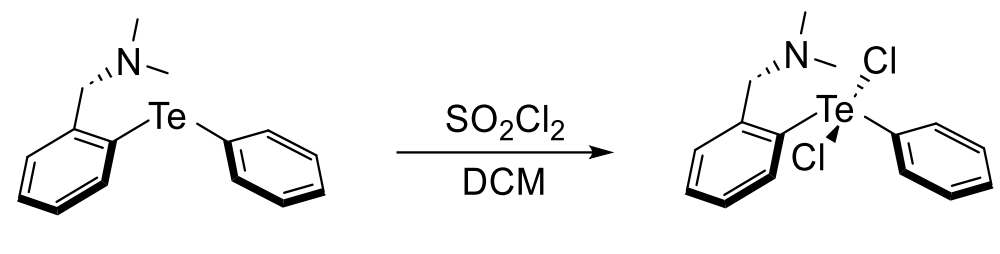

2 **9**

9: Yellow powder (45 mg, 0.11 mmol, 89% yield). ^{1}**H NMR** (500 MHz, CD_2Cl_2) δ 8.24–8.19 (m, 2H, Ar-*H*), 7.57–7.52 (m, 4H, Ar-*H*), 7.49-7.44 (m, Ar-*H*), 7.32 (d, J = 7.8 Hz, 2H, Ar-*H*), 4.05 (s, 2H, C*H*$_2$), 2.53 (s, 6H, C*H*$_3$). ^{13}**C{^{1}H} NMR** (126 MHz, $CDCl_3$) δ 139.7, 138.6, 138.2, 135.6, 134.4, 131.3, 130.9, 129.5, 129.2, 62.4, 44.6. ^{125}**Te{^{1}H} NMR** (126 MHz, $CDCl_3$) δ 975.1(s).

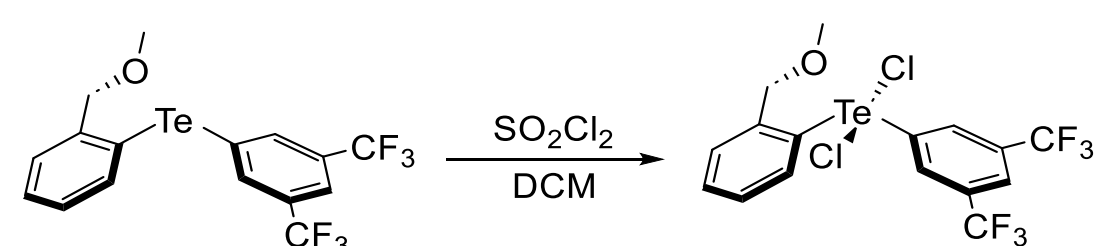

3 **10**

10: Yellow powder (61 mg, 0.12 mmol, 93% yield). ^{1}**H NMR** (400 MHz, CD_2Cl_2) δ 8.81 (s, 2H, Ar-*H*), 8.12 (s, 1H, Ar-*H*), 7.56–7.51 (m, 1H, Ar-*H*), 7.42–7.34 (m, 3H, Ar-*H*), 5.01 (s, 2H, C*H*$_2$), 3.70 (s, 3H, C*H*$_3$). ^{13}**C{^{1}H} NMR** (126 MHz, $CDCl_3$) δ 139.3, 138.2, 136.8, 135.9 (d, J = 3.9 Hz), 133.6, 132.7 (q, J = 34.0 Hz), 131.8, 130.2, 128.2, 125.4 (m), 122.9 (q, J = 273.5 Hz), 73.7, 58.7. ^{19}**F NMR** (377 MHz, $CDCl_3$) δ -62.71 (s). ^{125}**Te{^{1}H} MR** (126 MHz, $CDCl_3$) δ 943.0 (s).

1.3 NMR Spectra

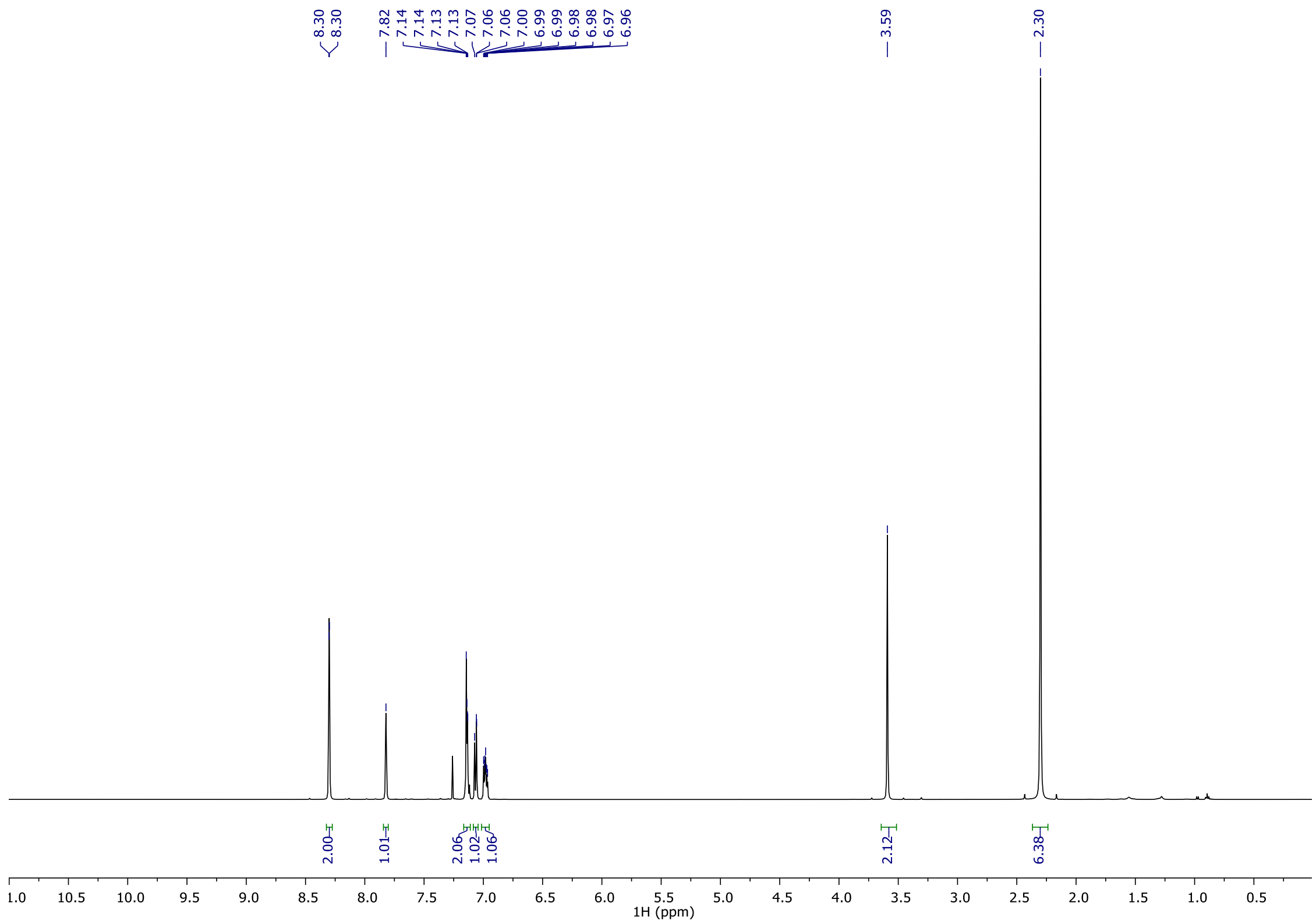

Figure S1. ^{1}H NMR spectrum of **1** in CD_2Cl_2.

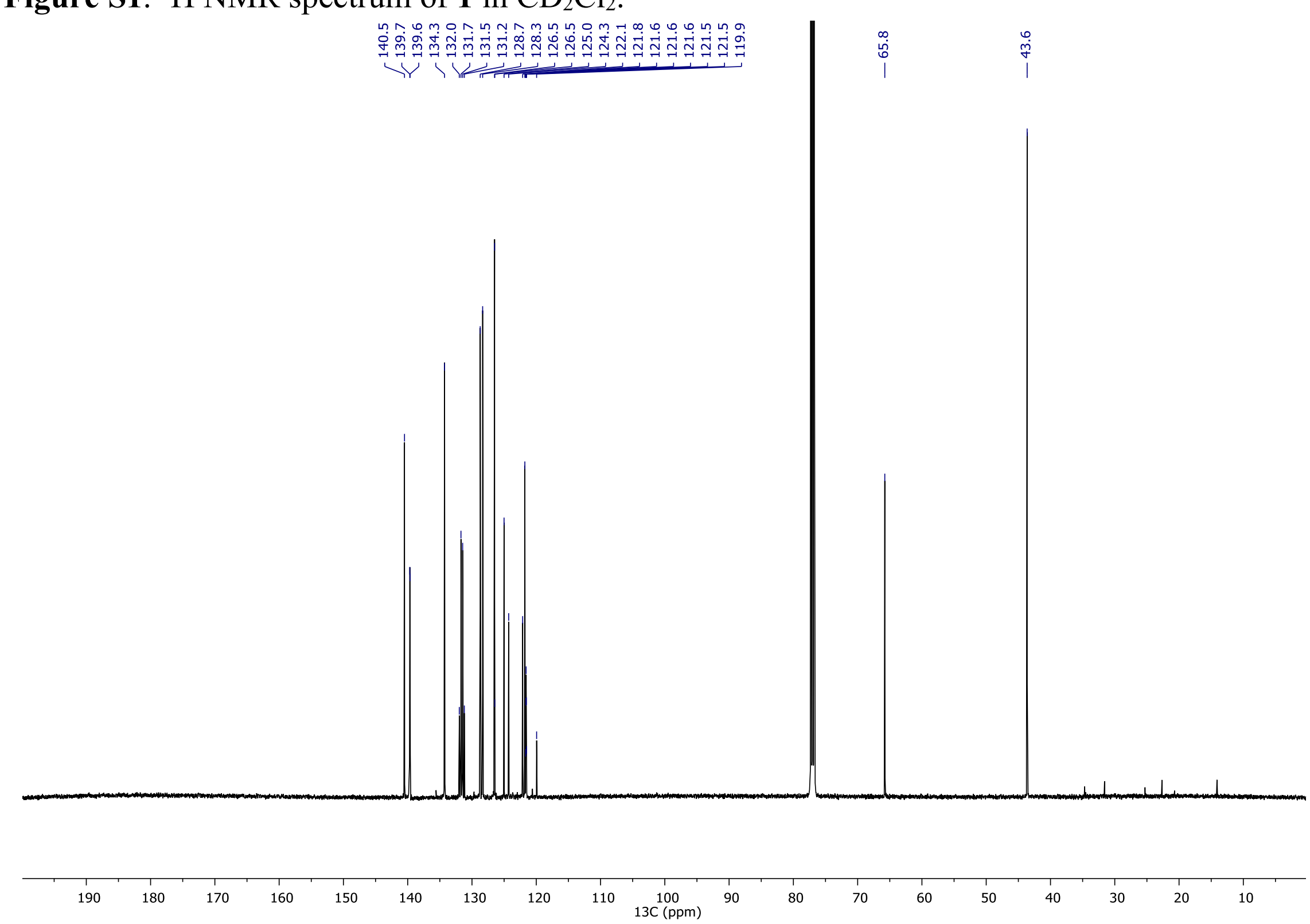

Figure S2. $^{13}C\{^1H\}$ NMR spectrum of **1** in CD_2Cl_2.

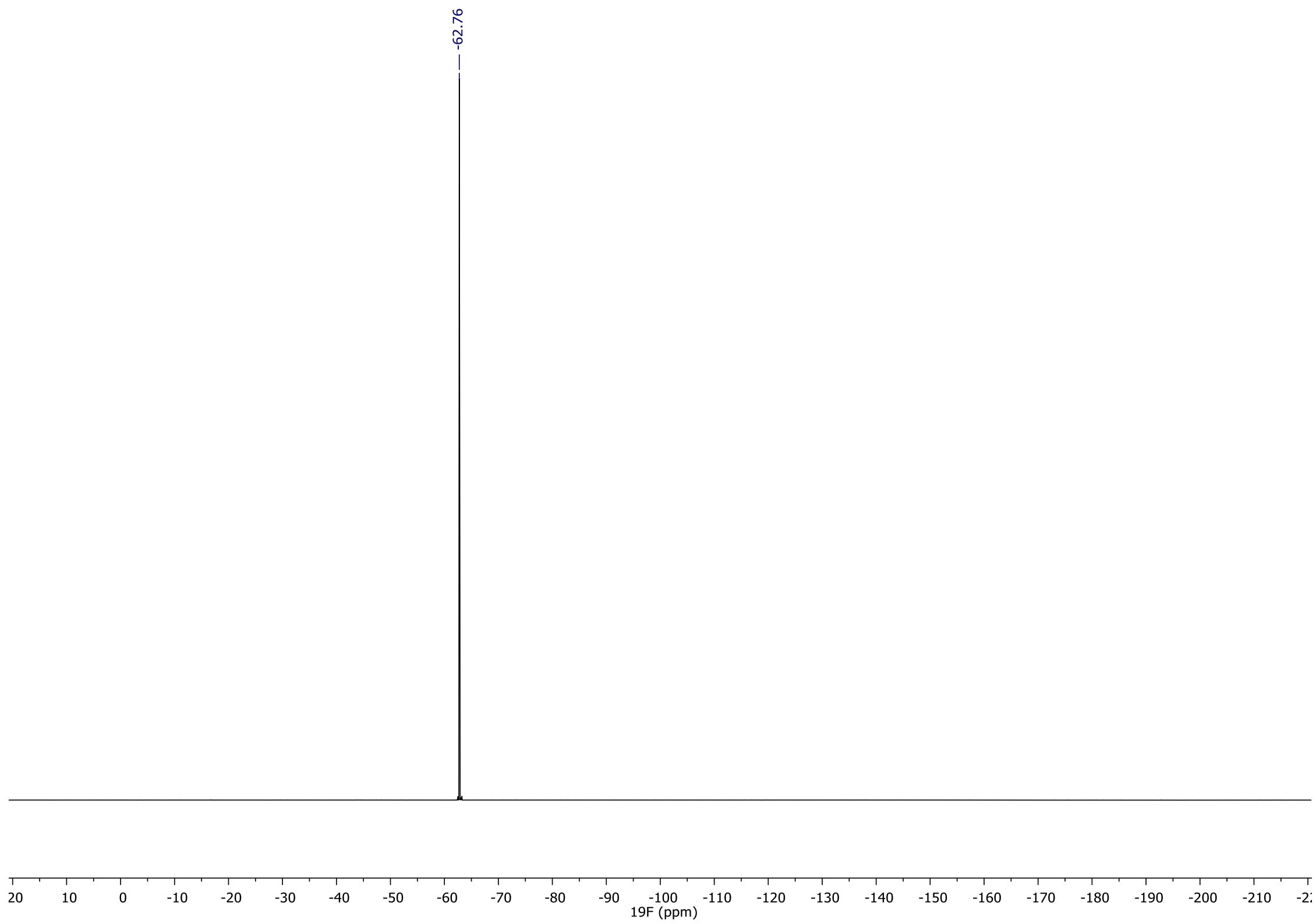

Figure S3. ^{19}F NMR spectrum of **1** in CD_2Cl_2.

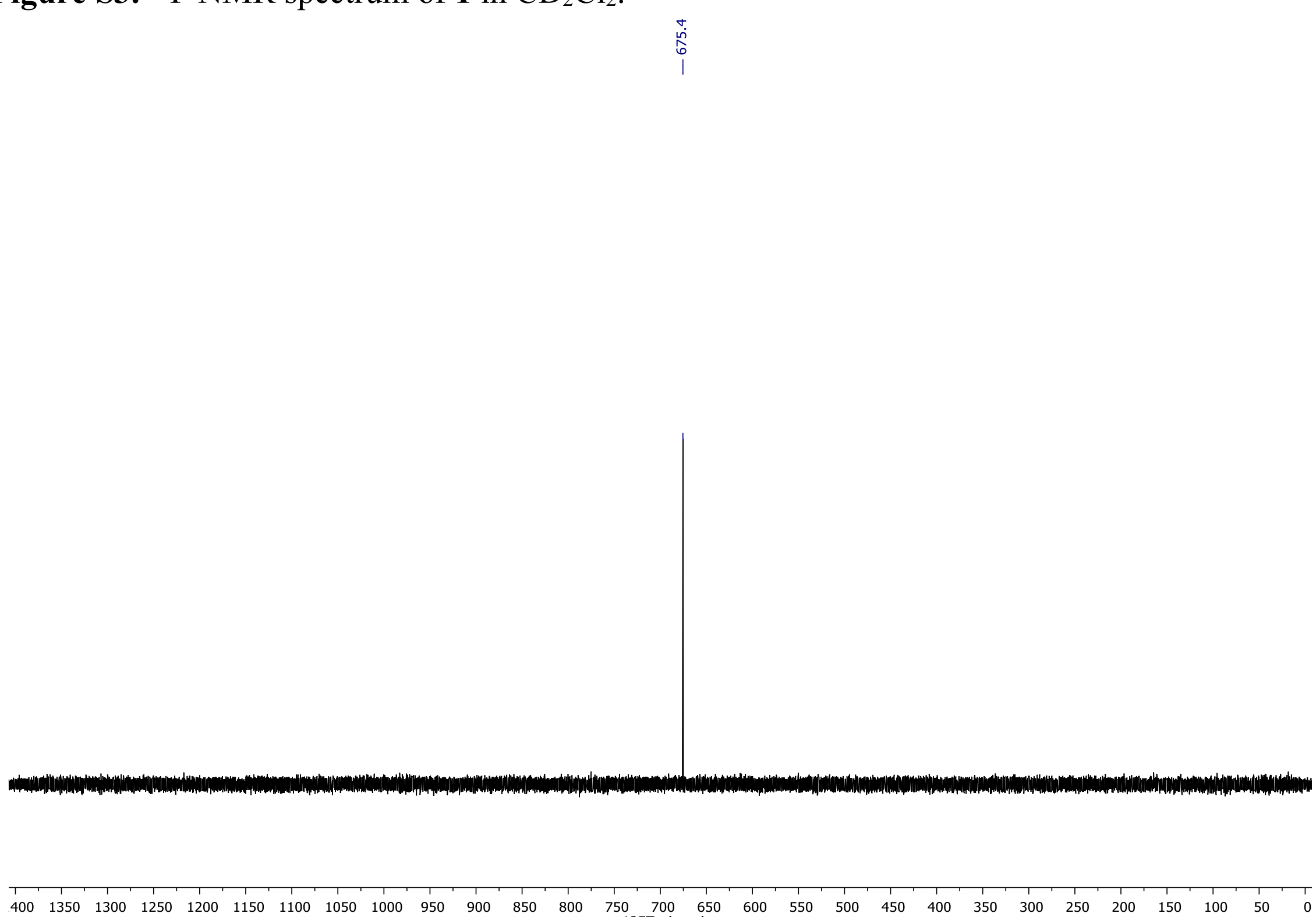

Figure S4. $^{125}Te\{^1H\}$ NMR spectrum of **1** in CD_2Cl_2.

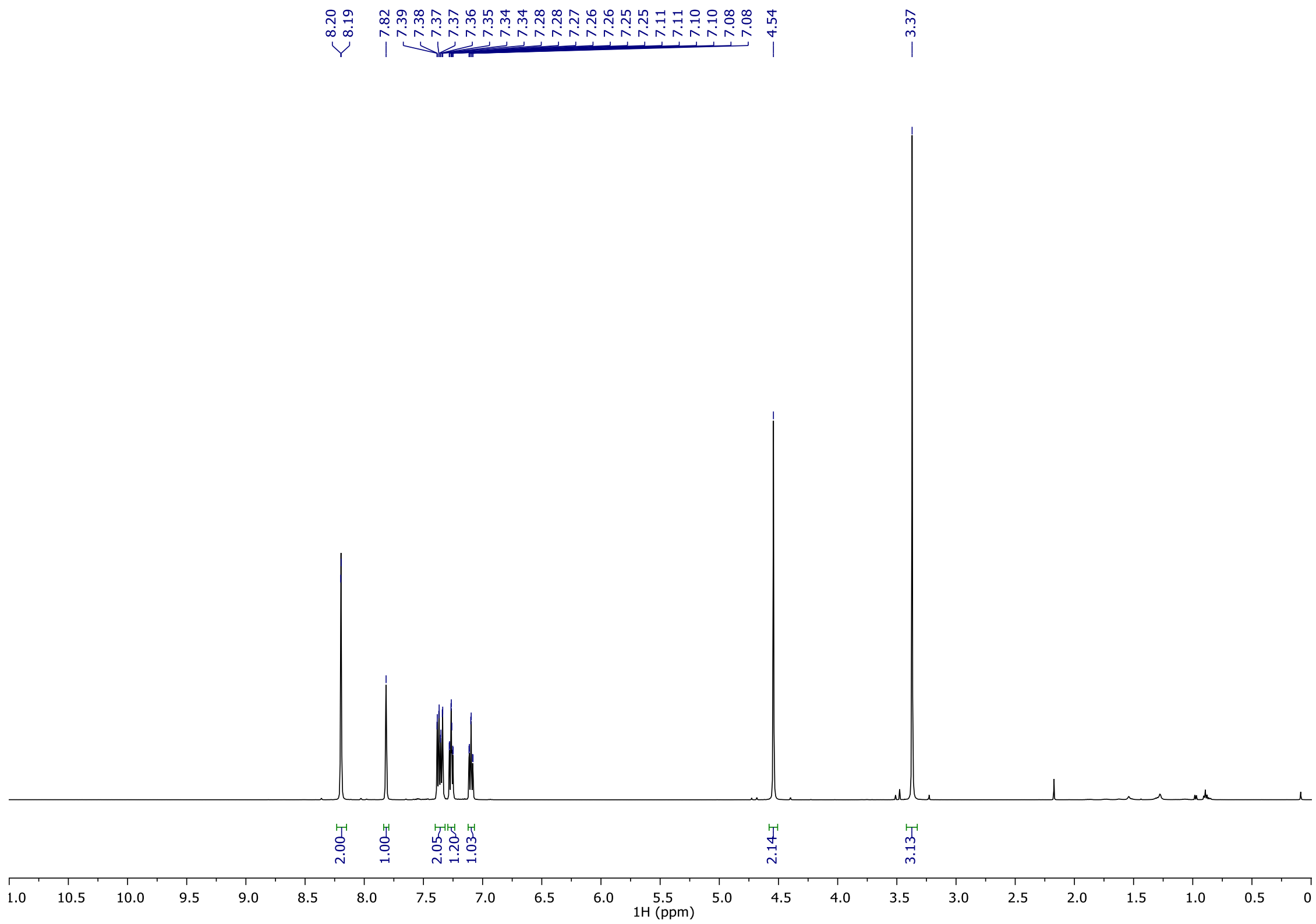

Figure S5. ^{1}H NMR spectrum of **3** in CD_2Cl_2.

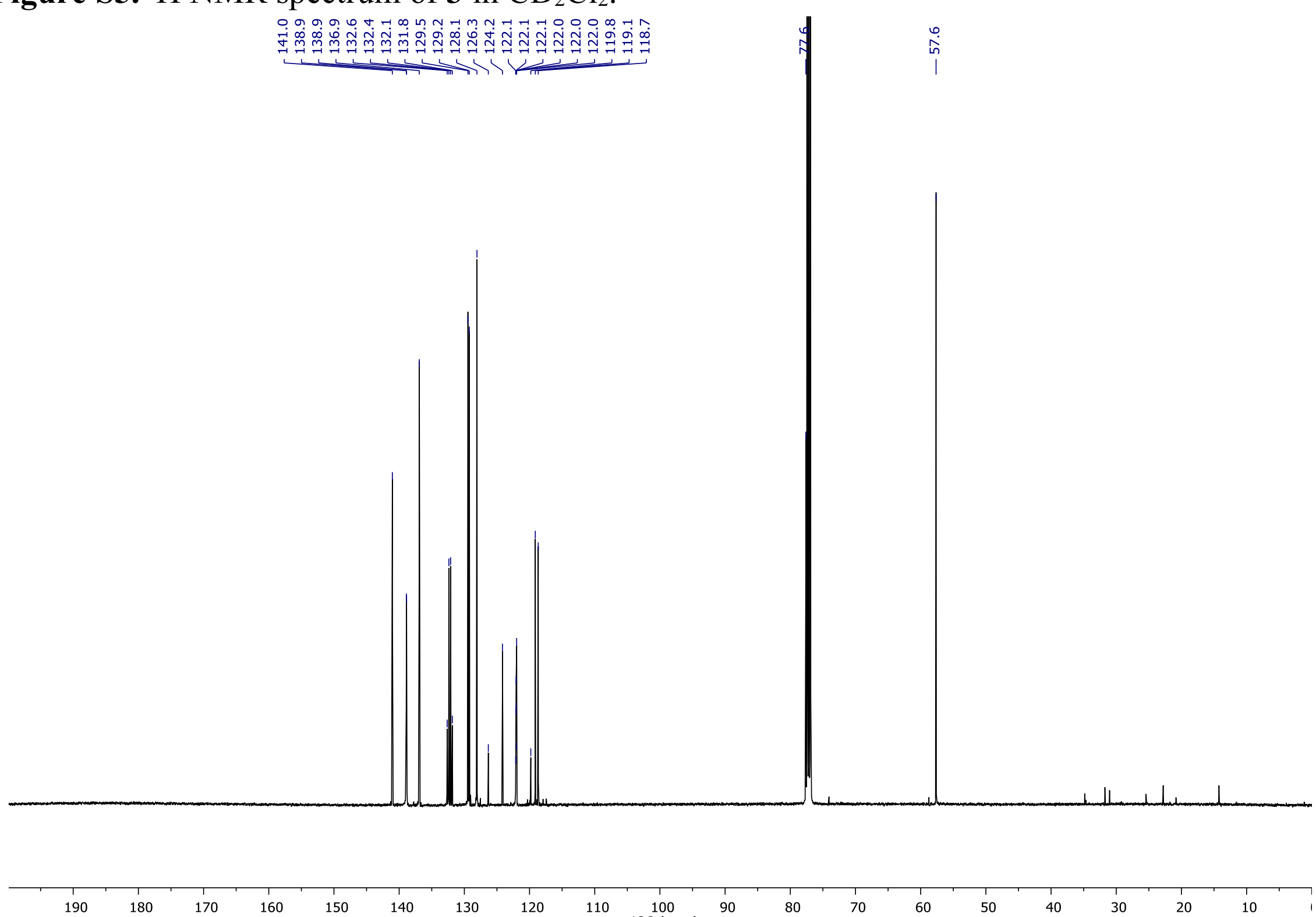

Figure S6 $^{13}C\{^{1}H\}$ NMR spectrum of **3** in CD_2Cl_2.

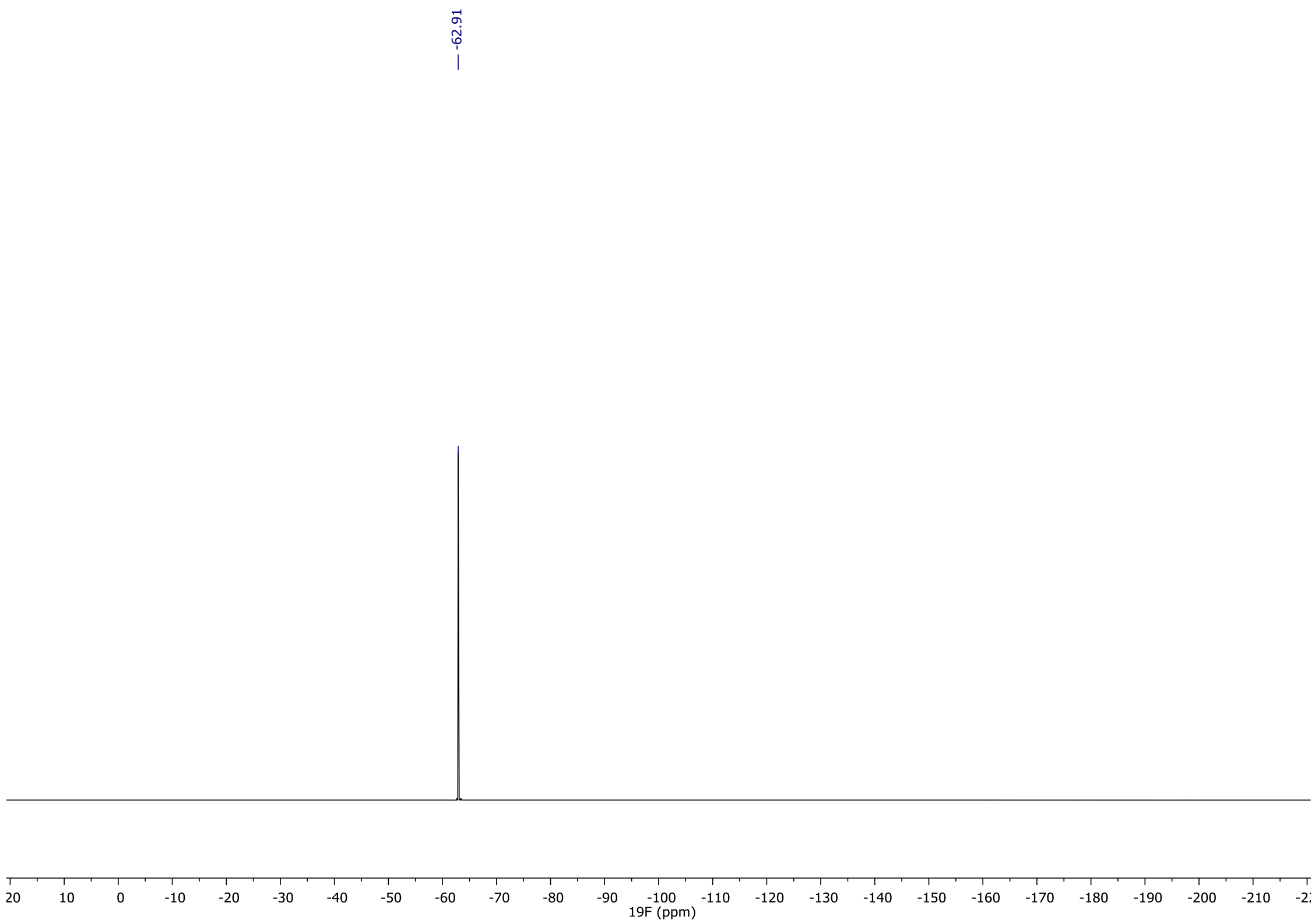

Figure S7. ^{19}F NMR spectrum of **3** in CD_2Cl_2.

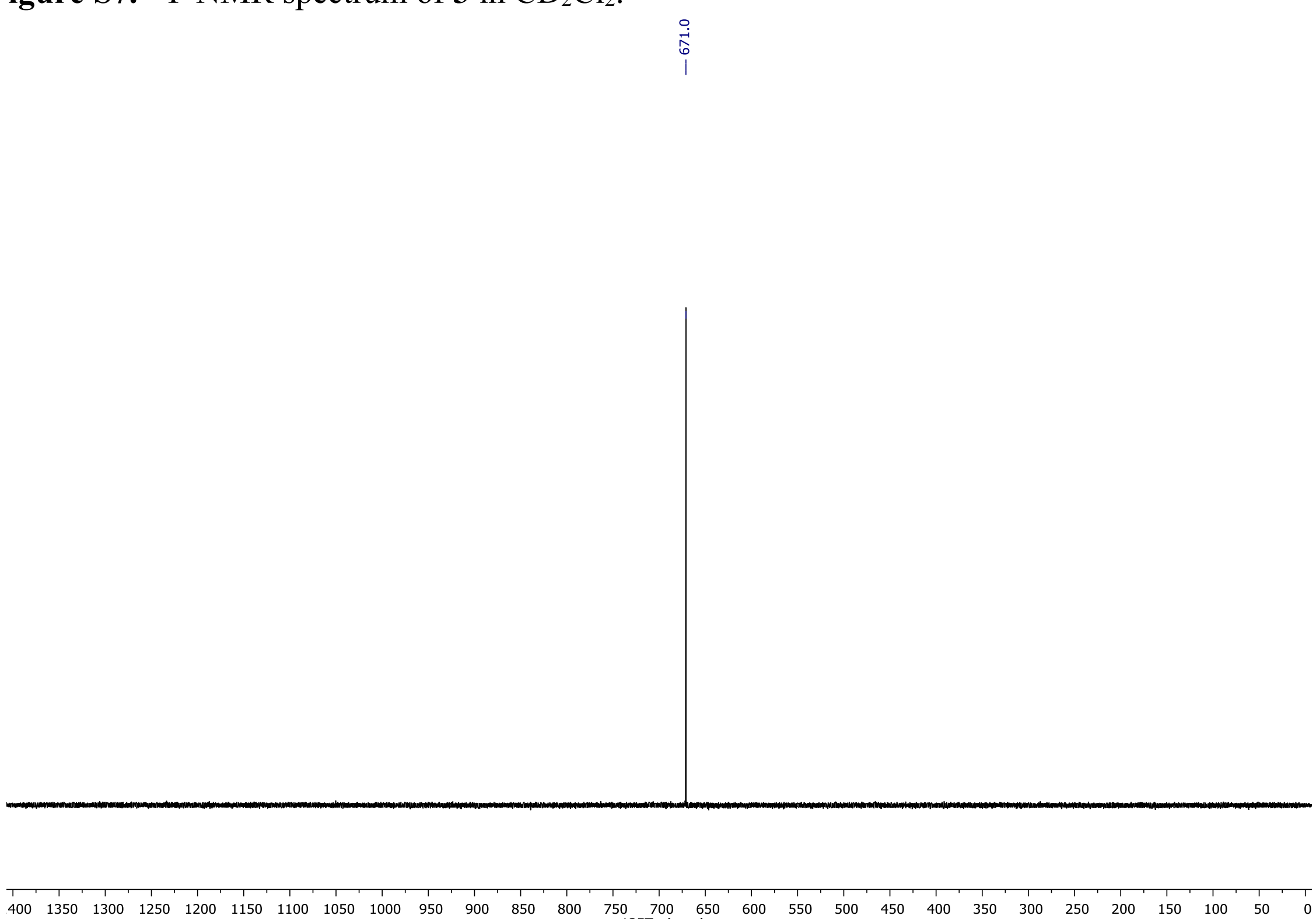

Figure S8. $^{125}Te\{^1H\}$ NMR spectrum of **3** in CD_2Cl_2.

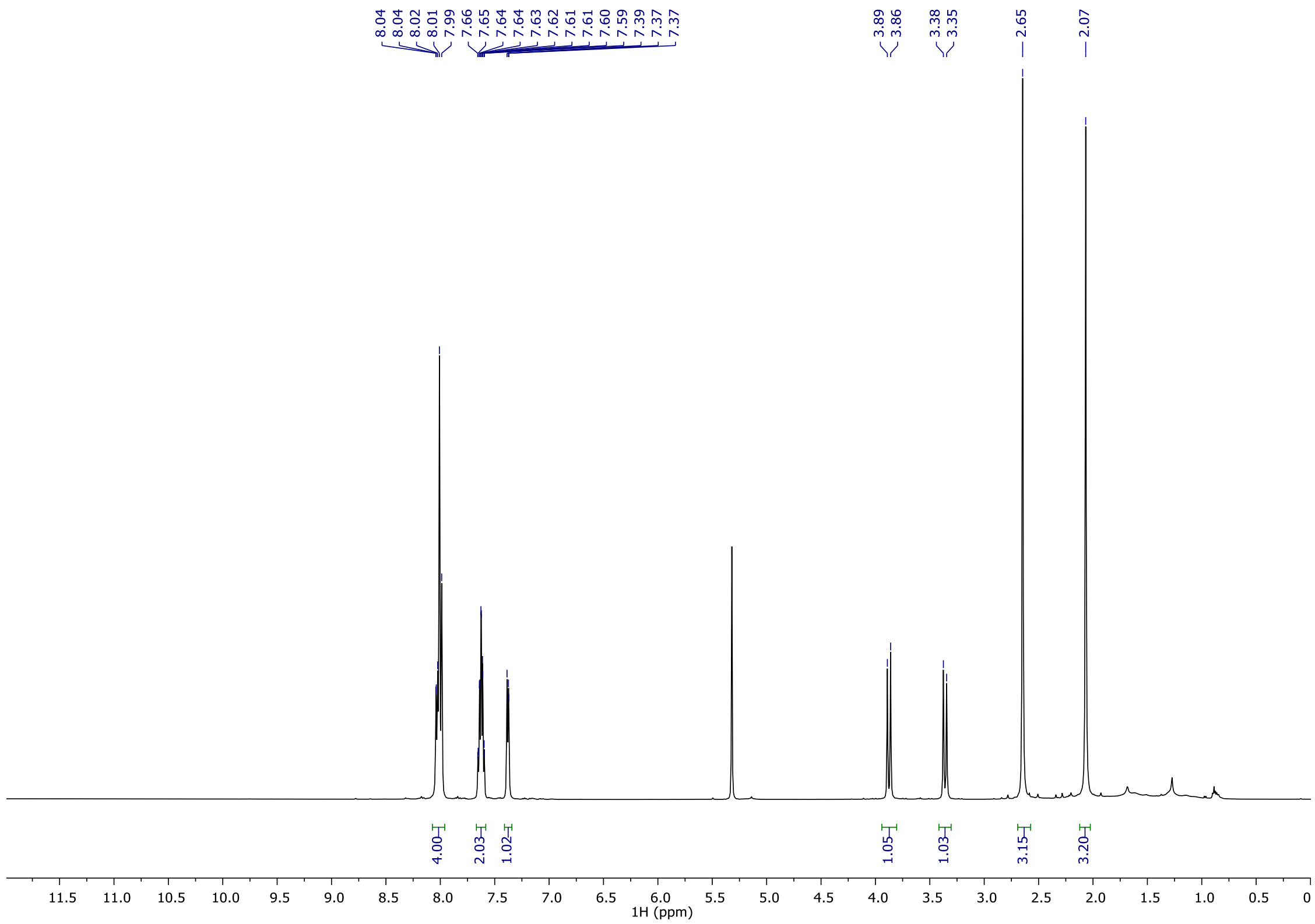

Figure S9. ^{1}H NMR spectrum of **4** in CD_2Cl_2.

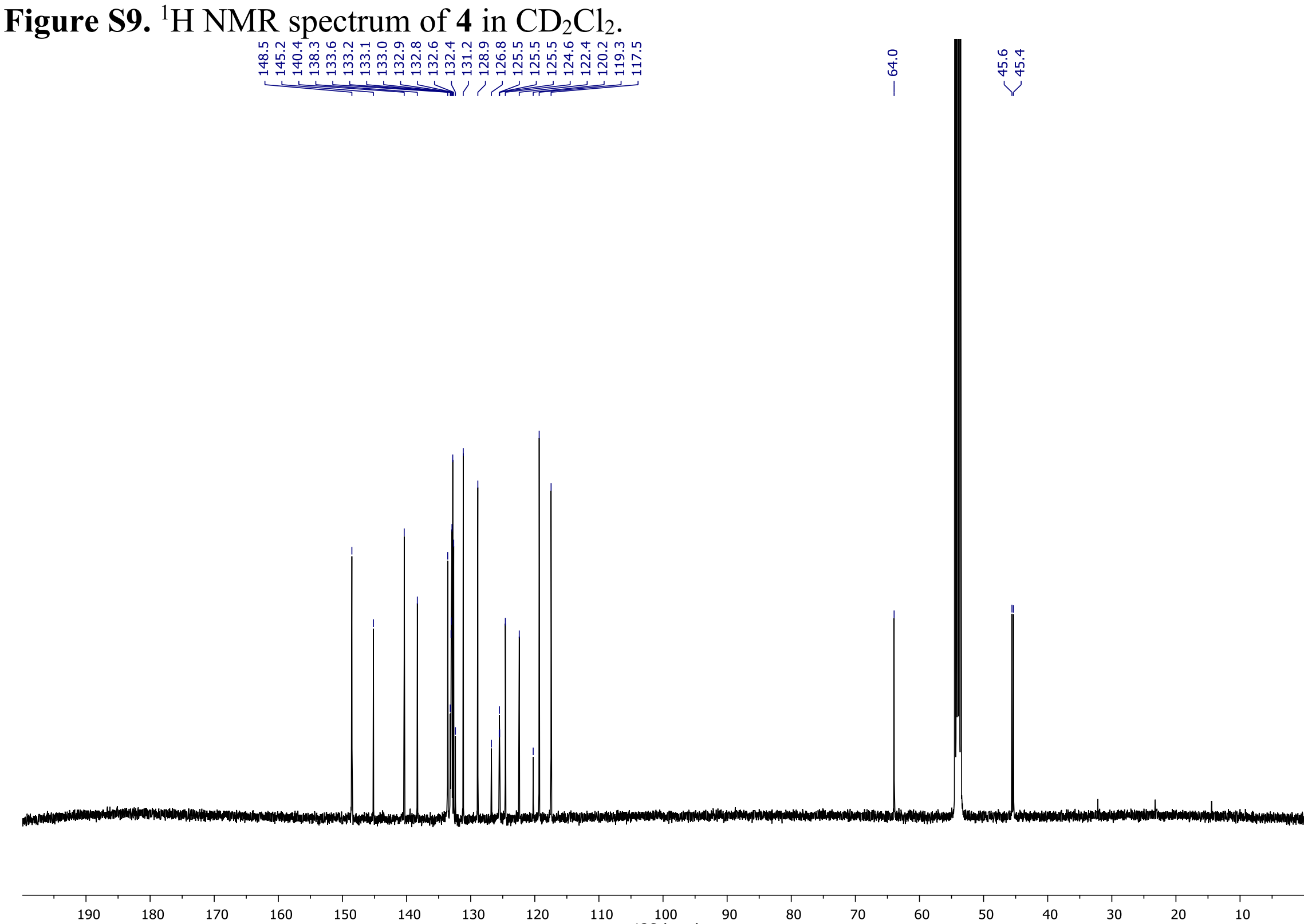

Figure S10. $^{13}C\{^{1}H\}$ NMR spectrum of **4** in CD_2Cl_2.

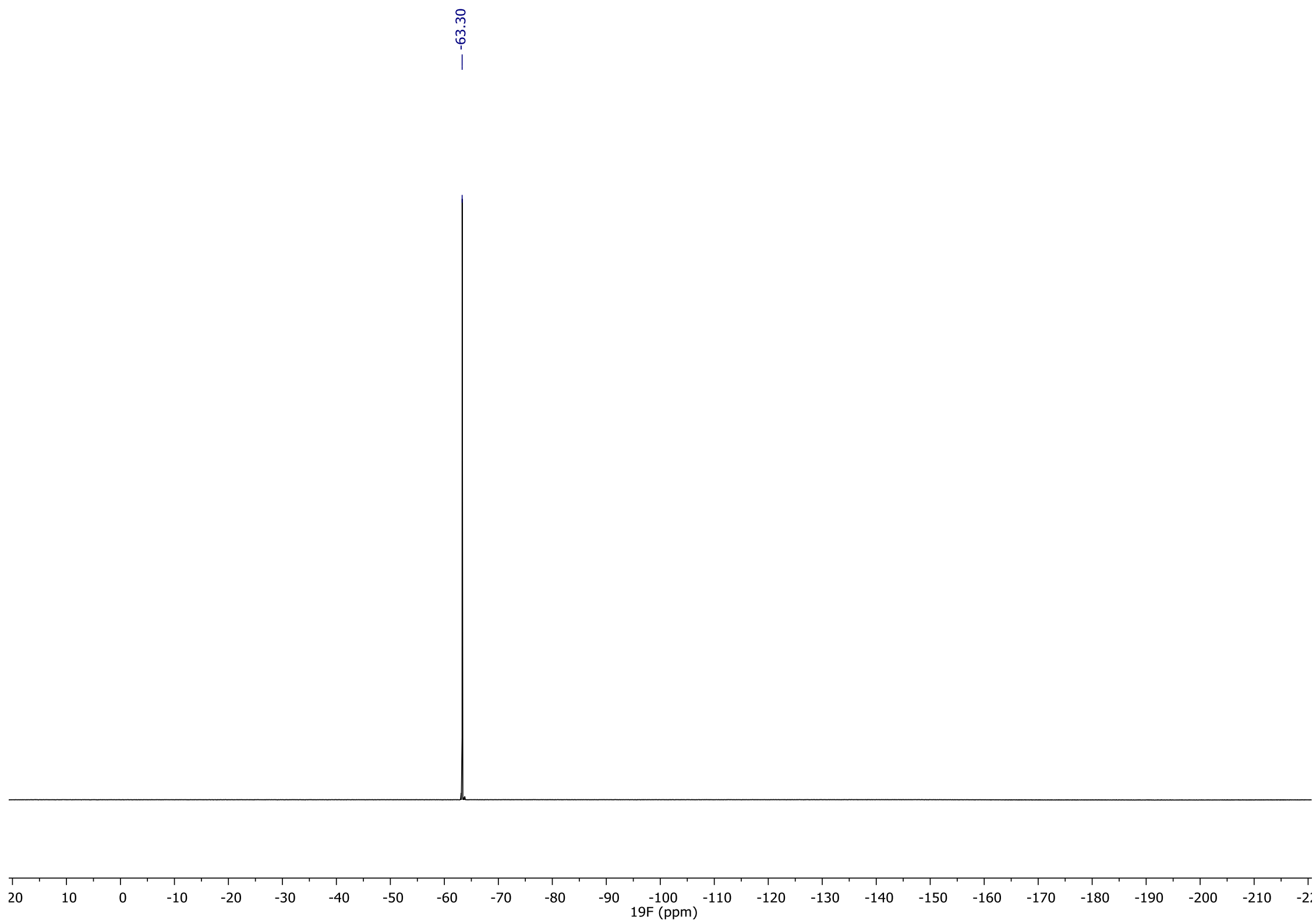

Figure S11. ^{19}F NMR spectrum of **4** in CD_2Cl_2.

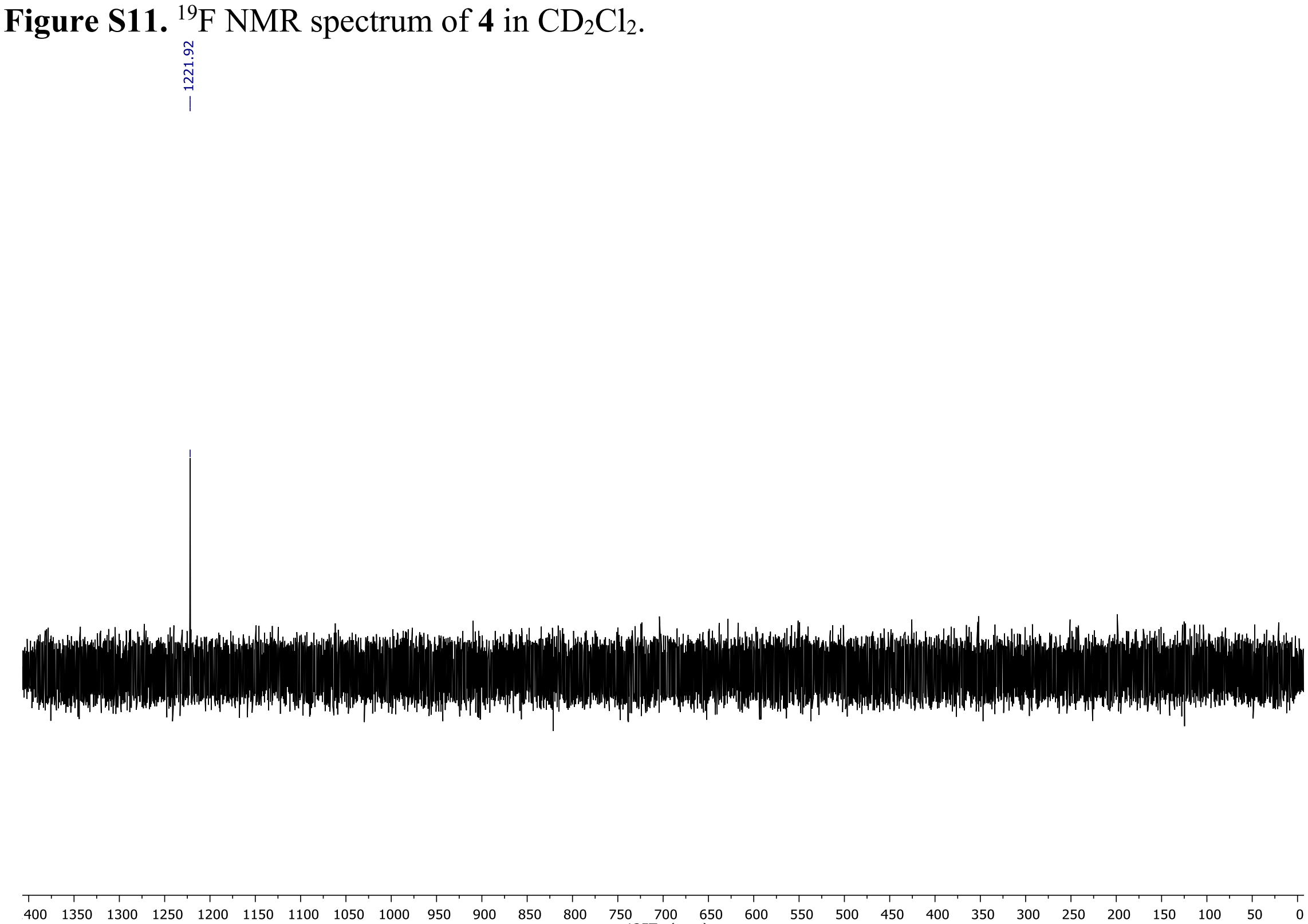

Figure S12. $^{125}Te\{^{1}H\}$ NMR spectrum of **4** in CD_2Cl_2.

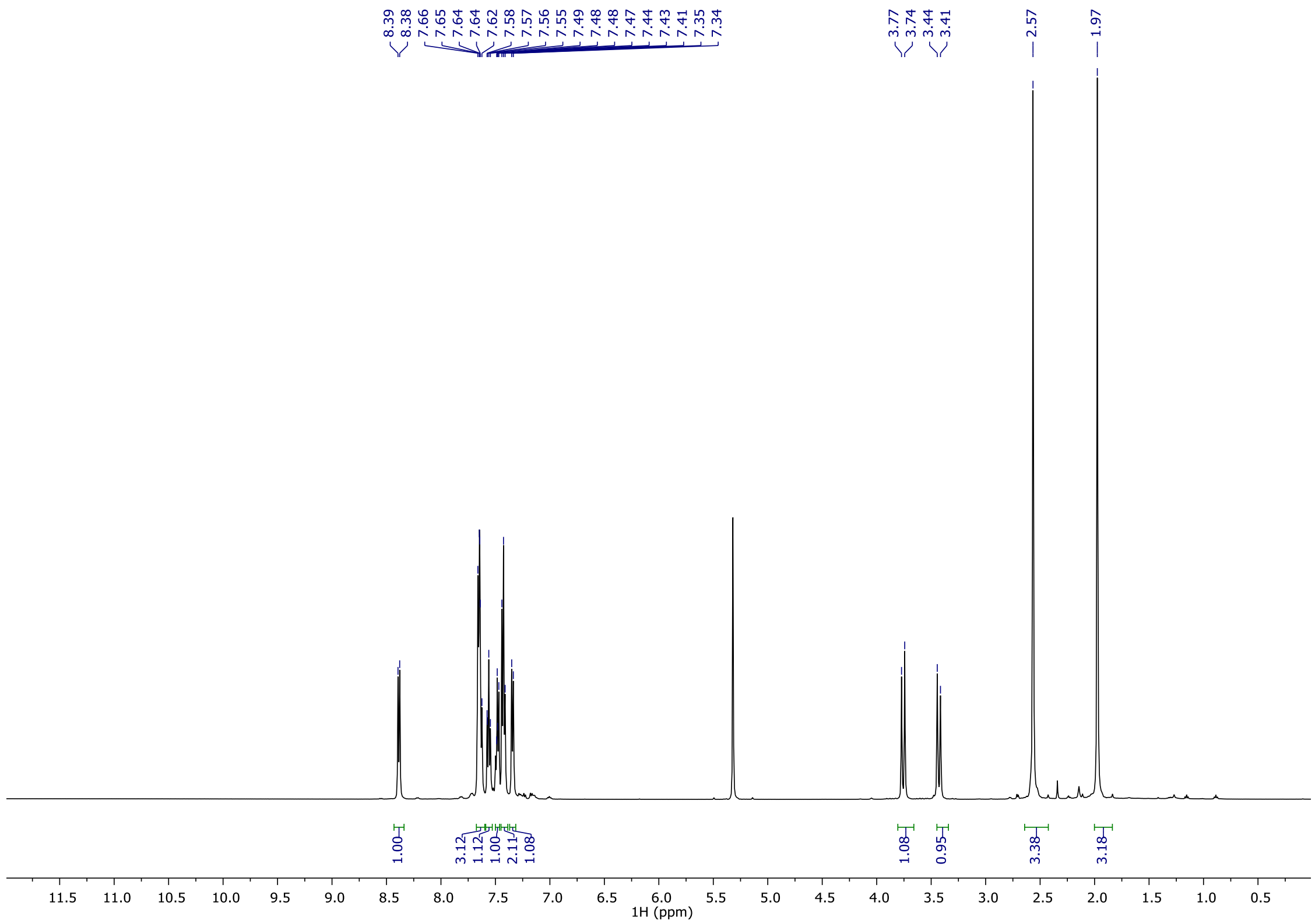

Figure S13. ^{1}H NMR spectrum of **5** in CD_2Cl_2.

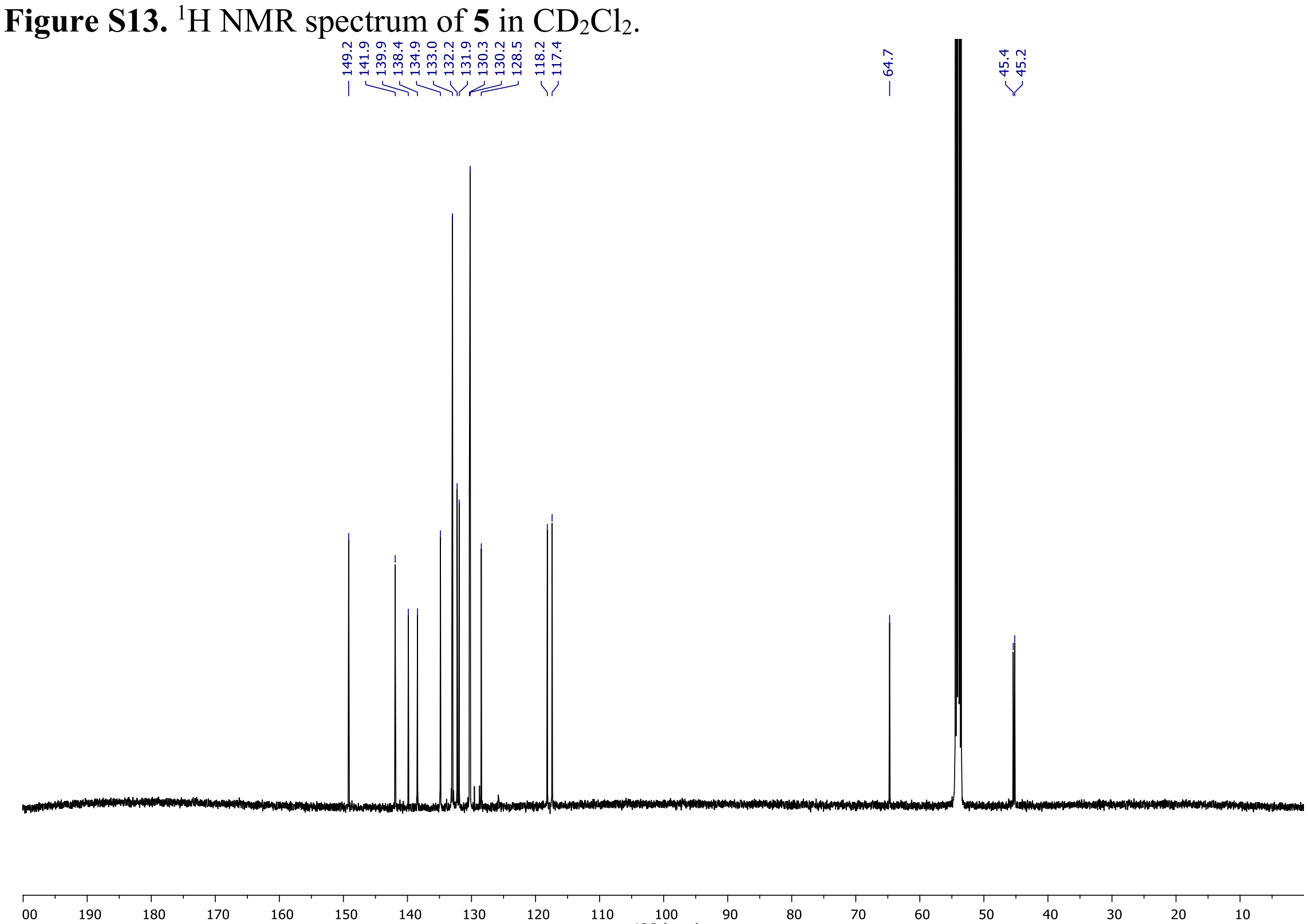

Figure S14. $^{13}C\{^{1}H\}$ NMR spectrum of **5** in CD_2Cl_2.

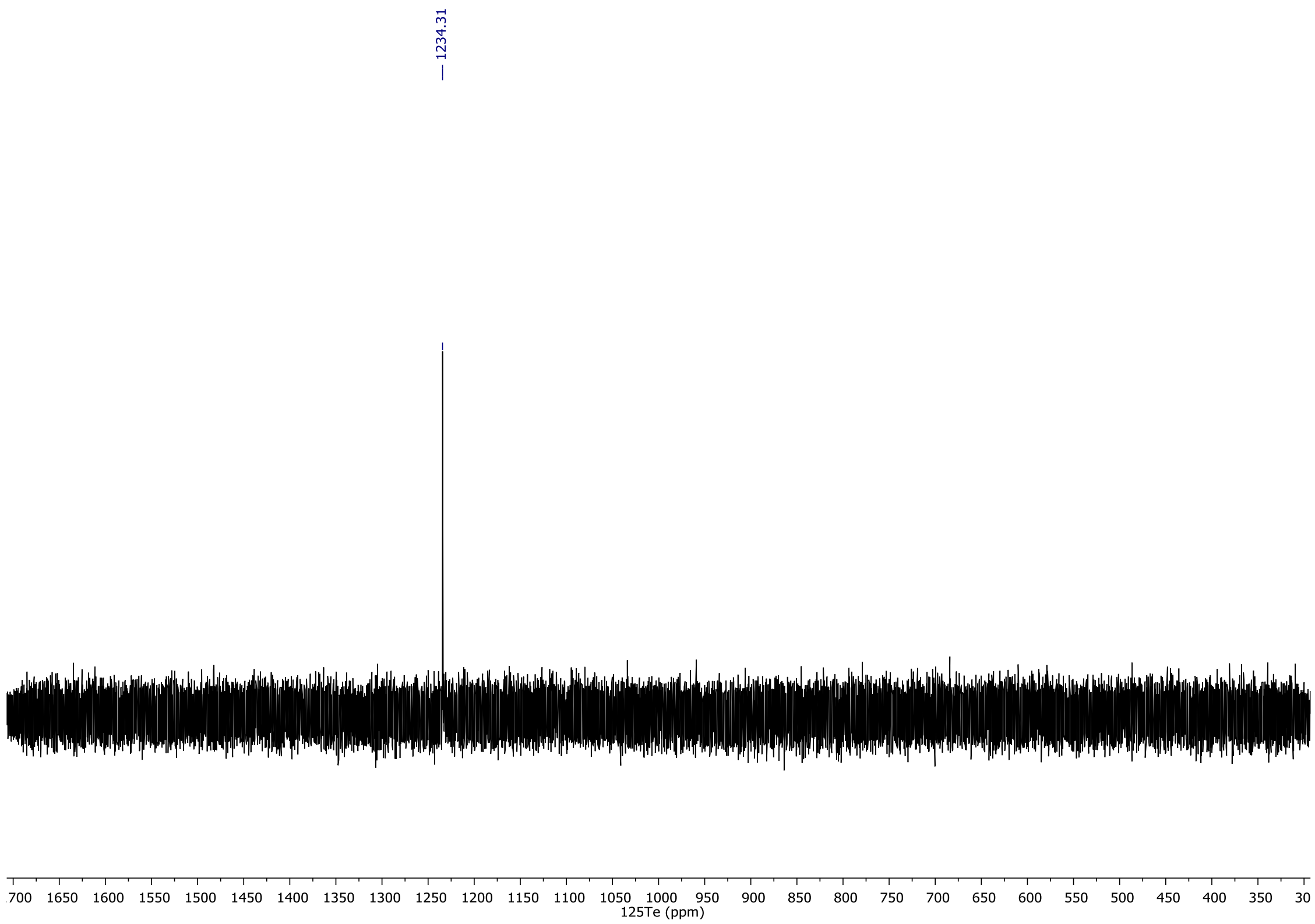

Figure S15. $^{125}Te\{^{1}H\}$ NMR spectrum of **5** in CD_2Cl_2.

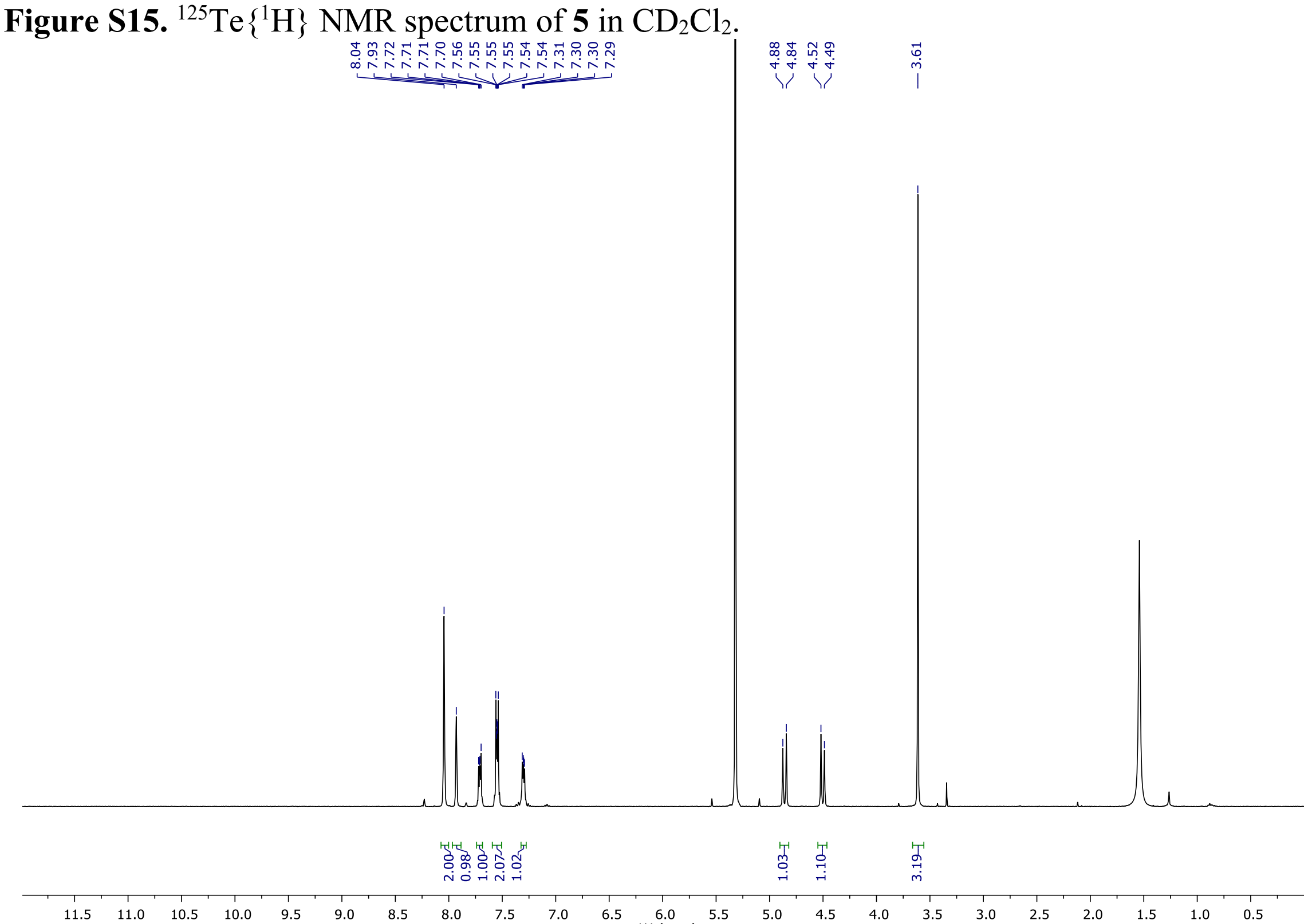

Figure S16. ^{1}H NMR spectrum of **6** in CD_2Cl_2.

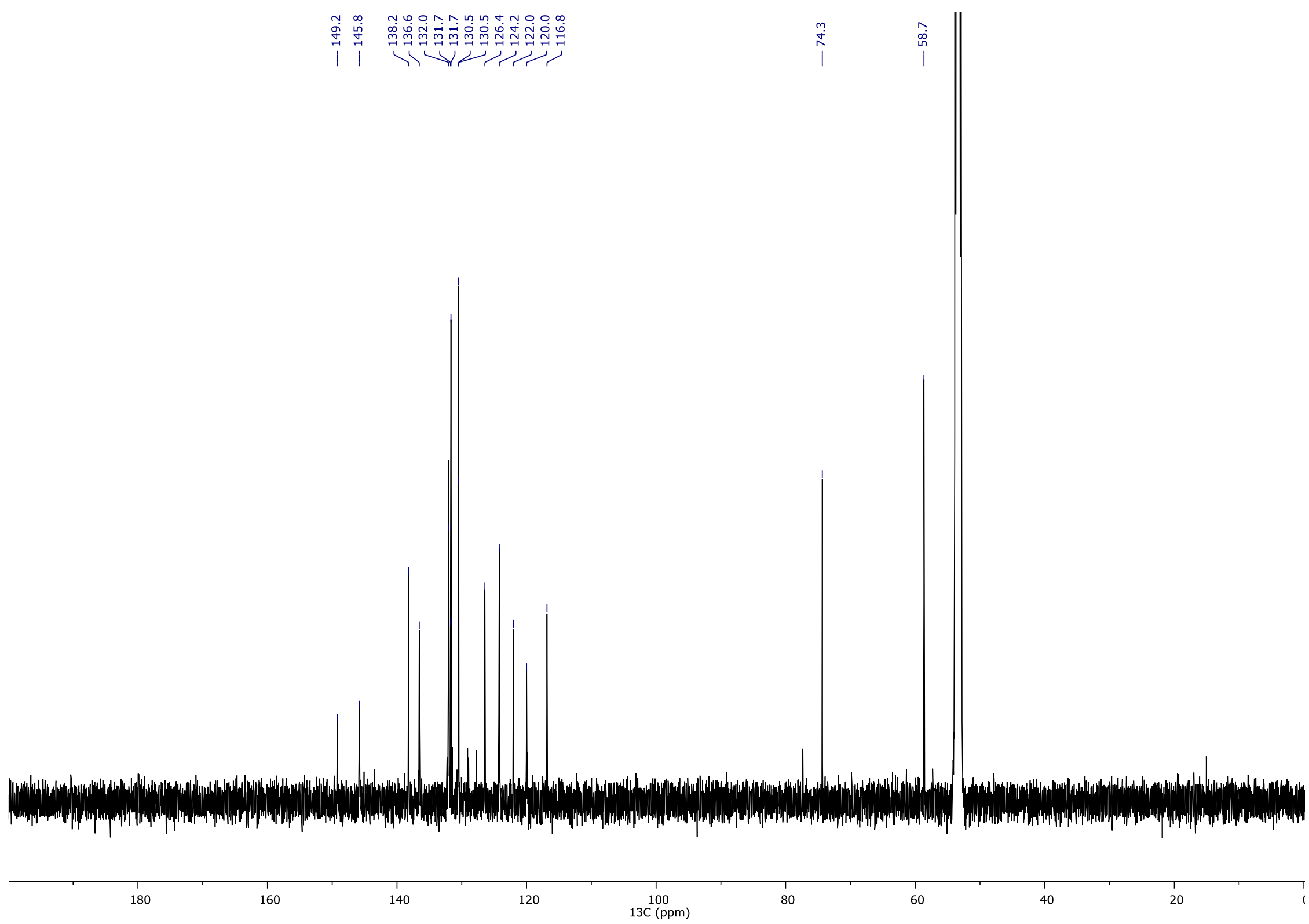

Figure S17. $^{13}C\{^{1}H\}$ NMR spectrum of **6** in CD_2Cl_2.

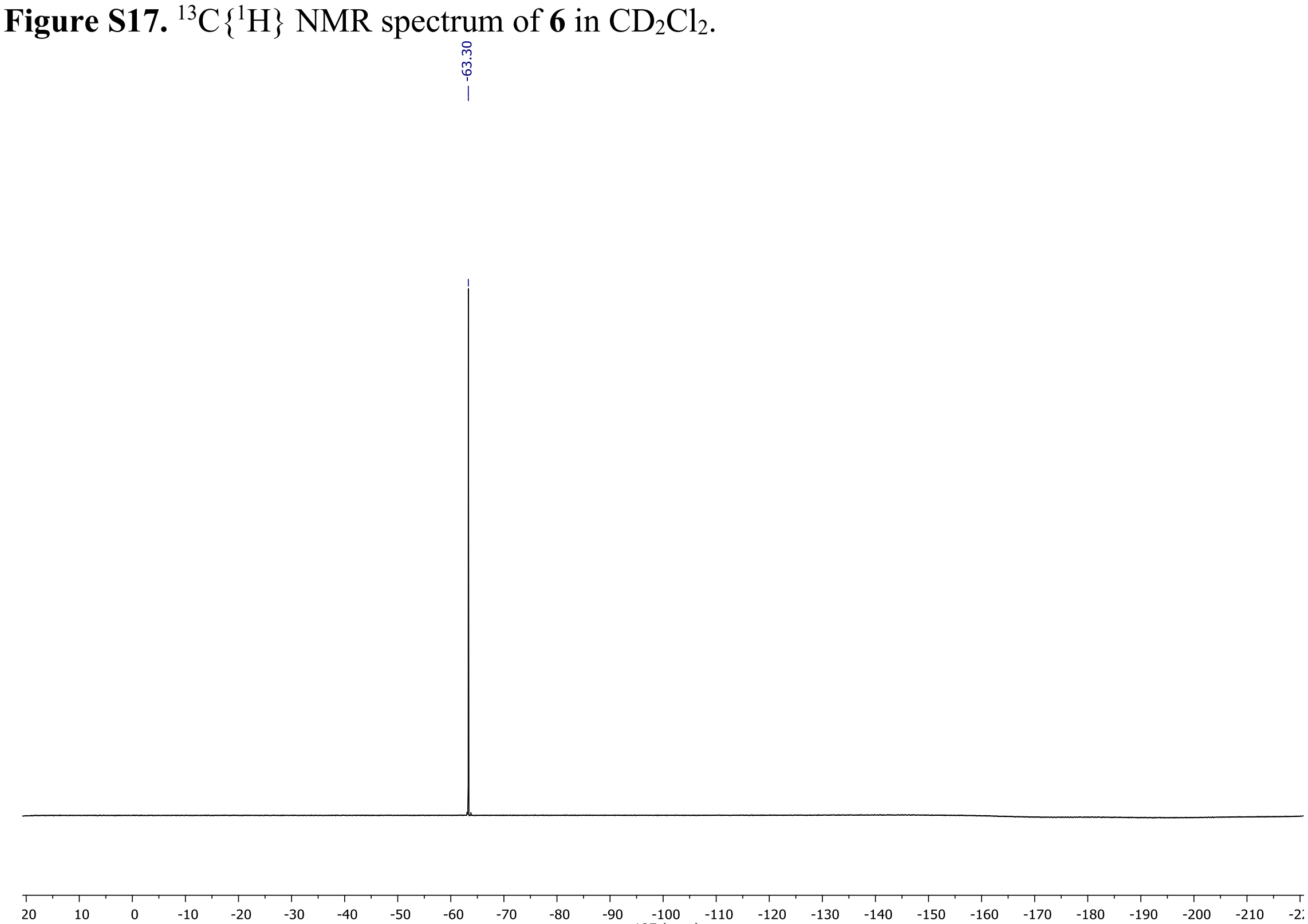

Figure S18. ^{19}F NMR spectrum of **6** in CD_2Cl_2.

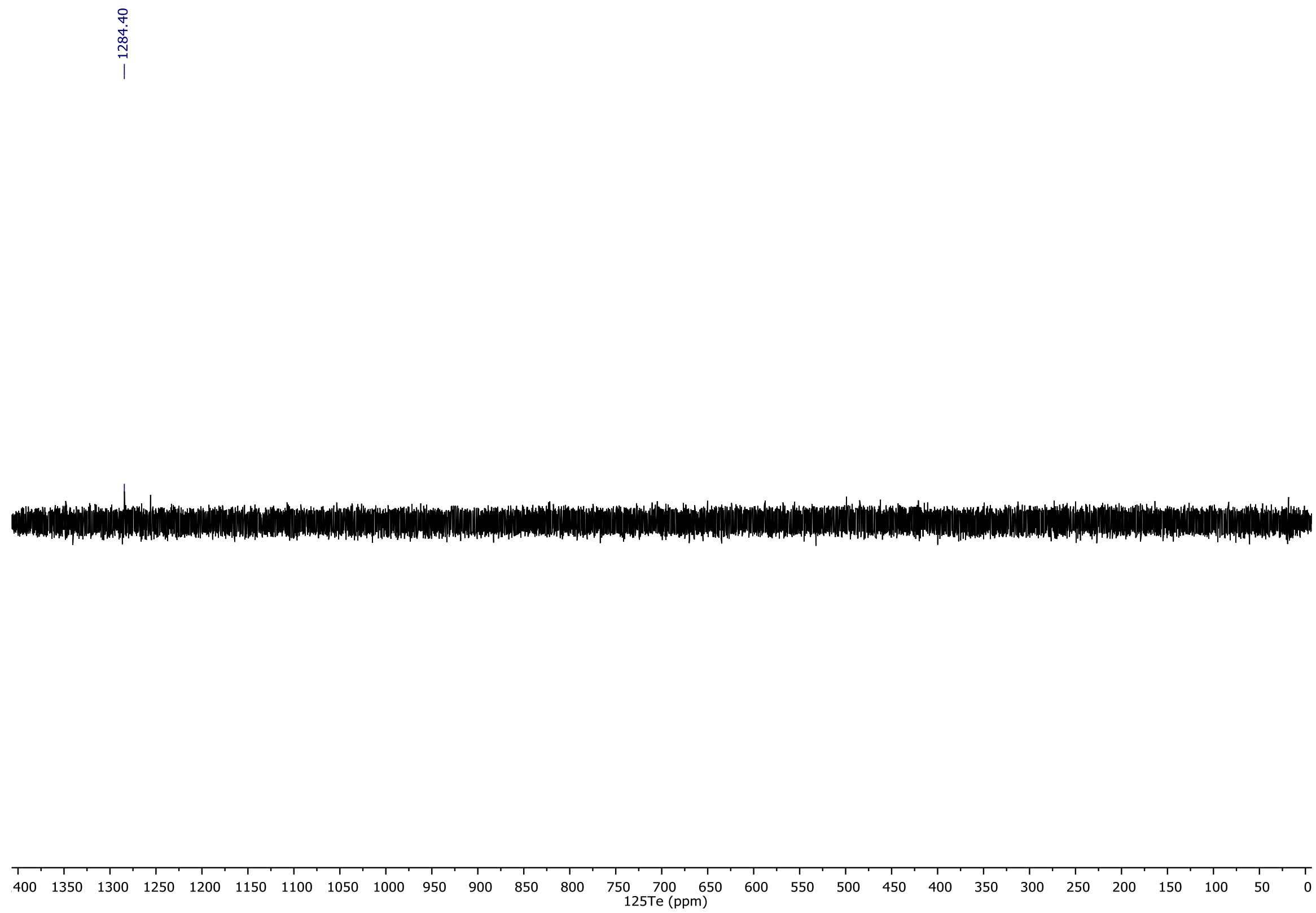

Figure S19. $^{125}Te\{^{1}H\}$ NMR spectrum of **6** in CD_2Cl_2.

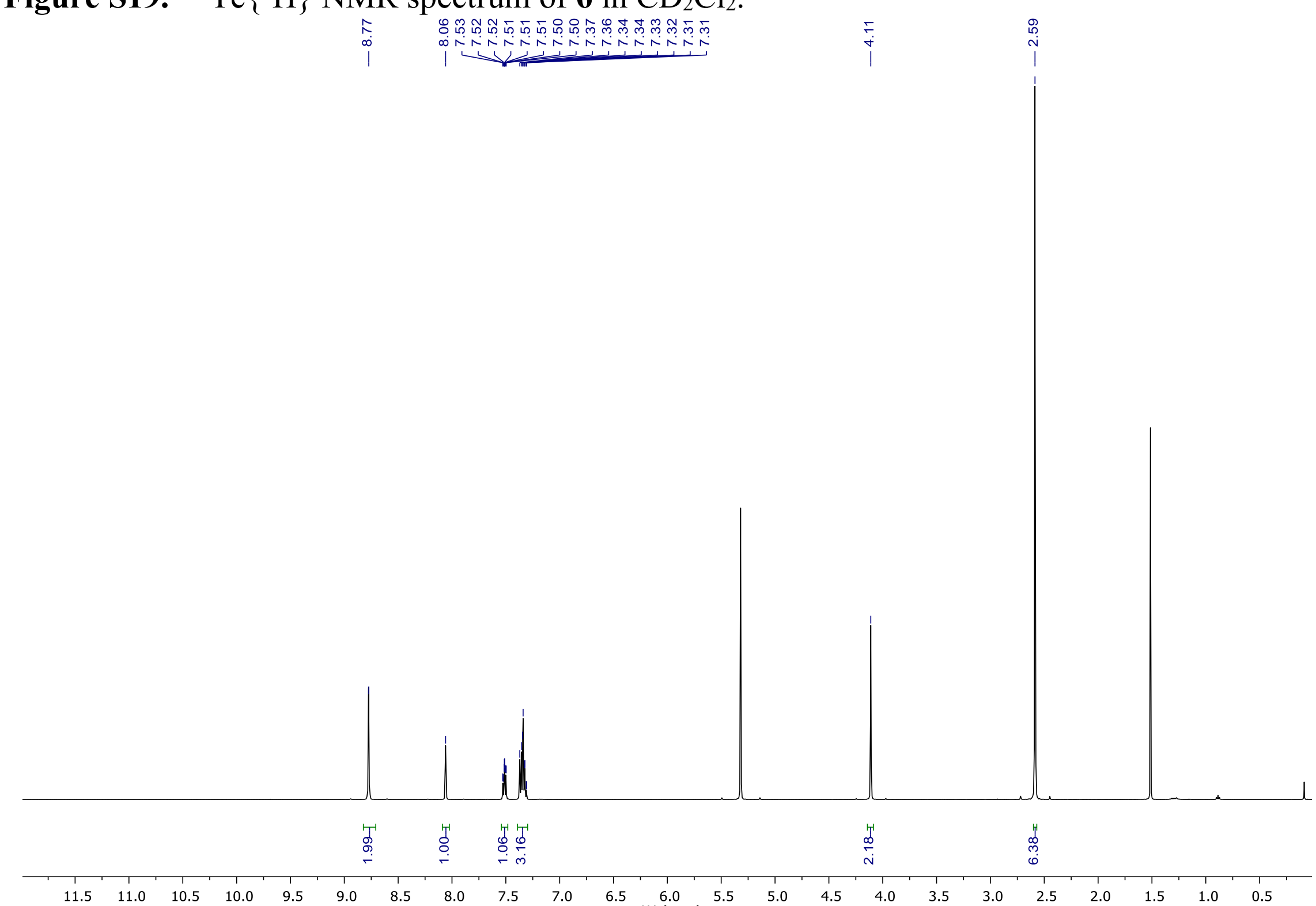

Figure S20. ^{1}H NMR spectrum of **8** in CD_2Cl_2.

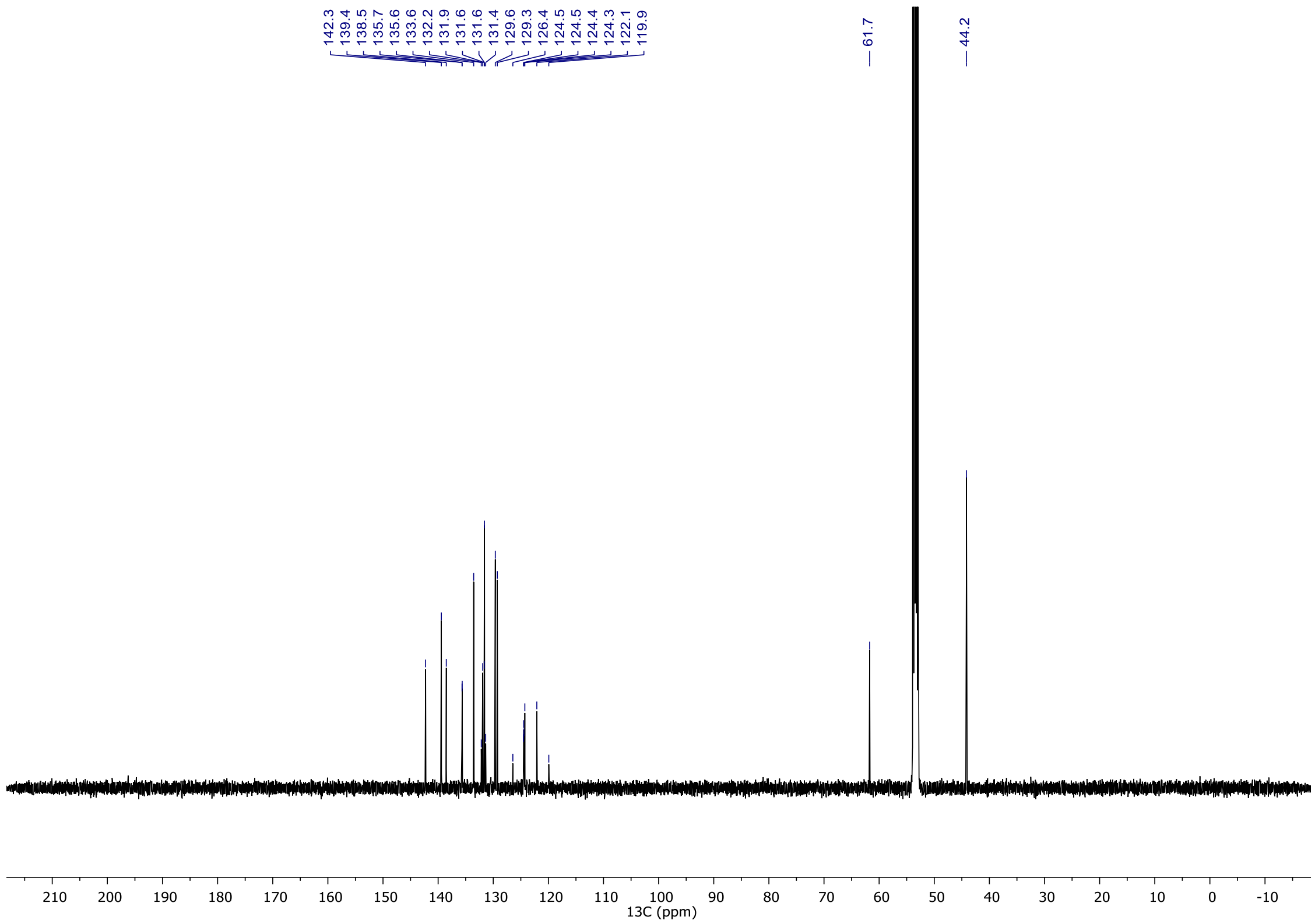

Figure S21. $^{13}C\{^1H\}$ NMR spectrum of **8** in CD_2Cl_2.

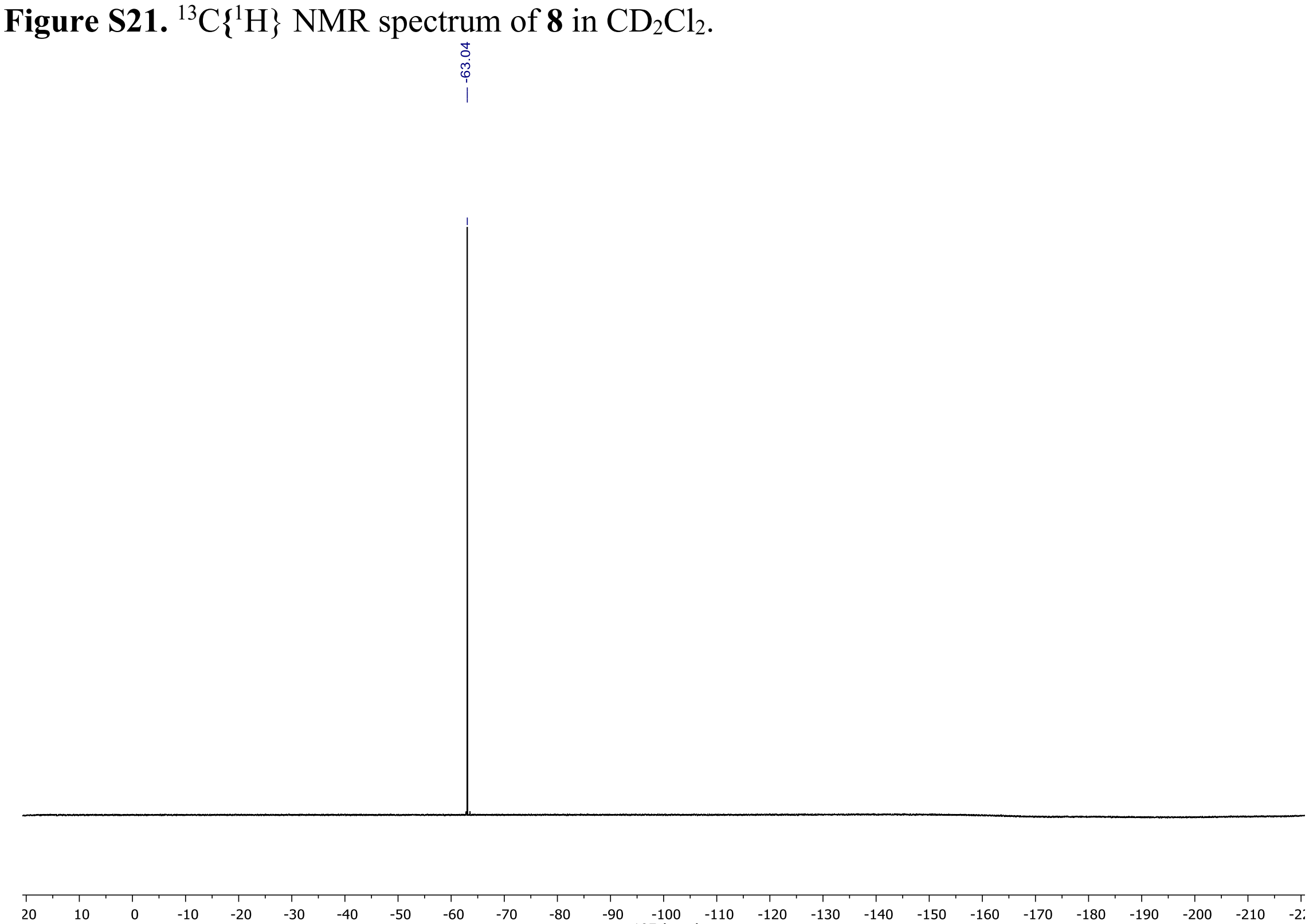

Figure S22. ^{19}F NMR spectrum of **8** in CD_2Cl_2.

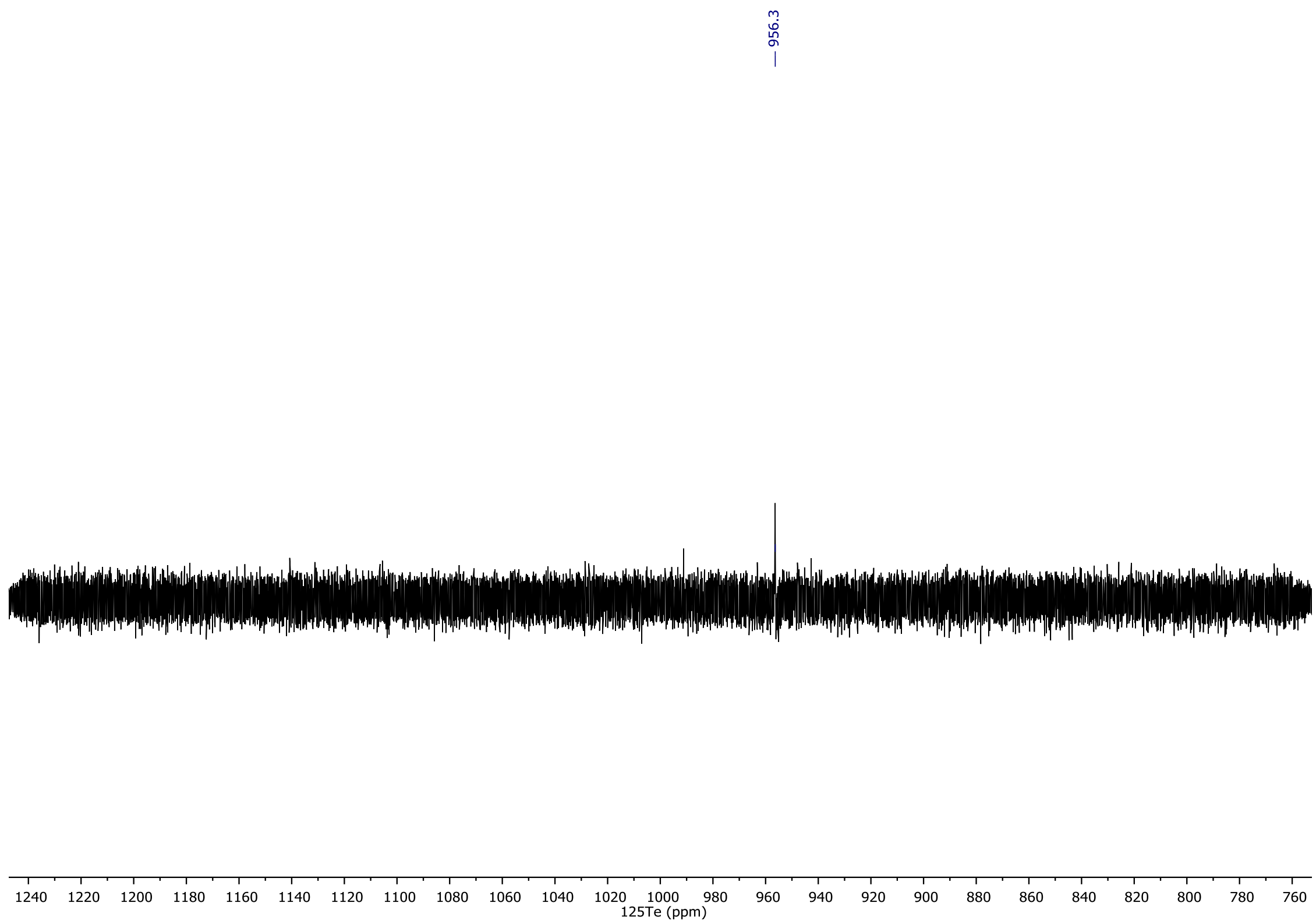

Figure S23. $^{125}Te\{^{1}H\}$ NMR spectrum of **8** in CD_2Cl_2.

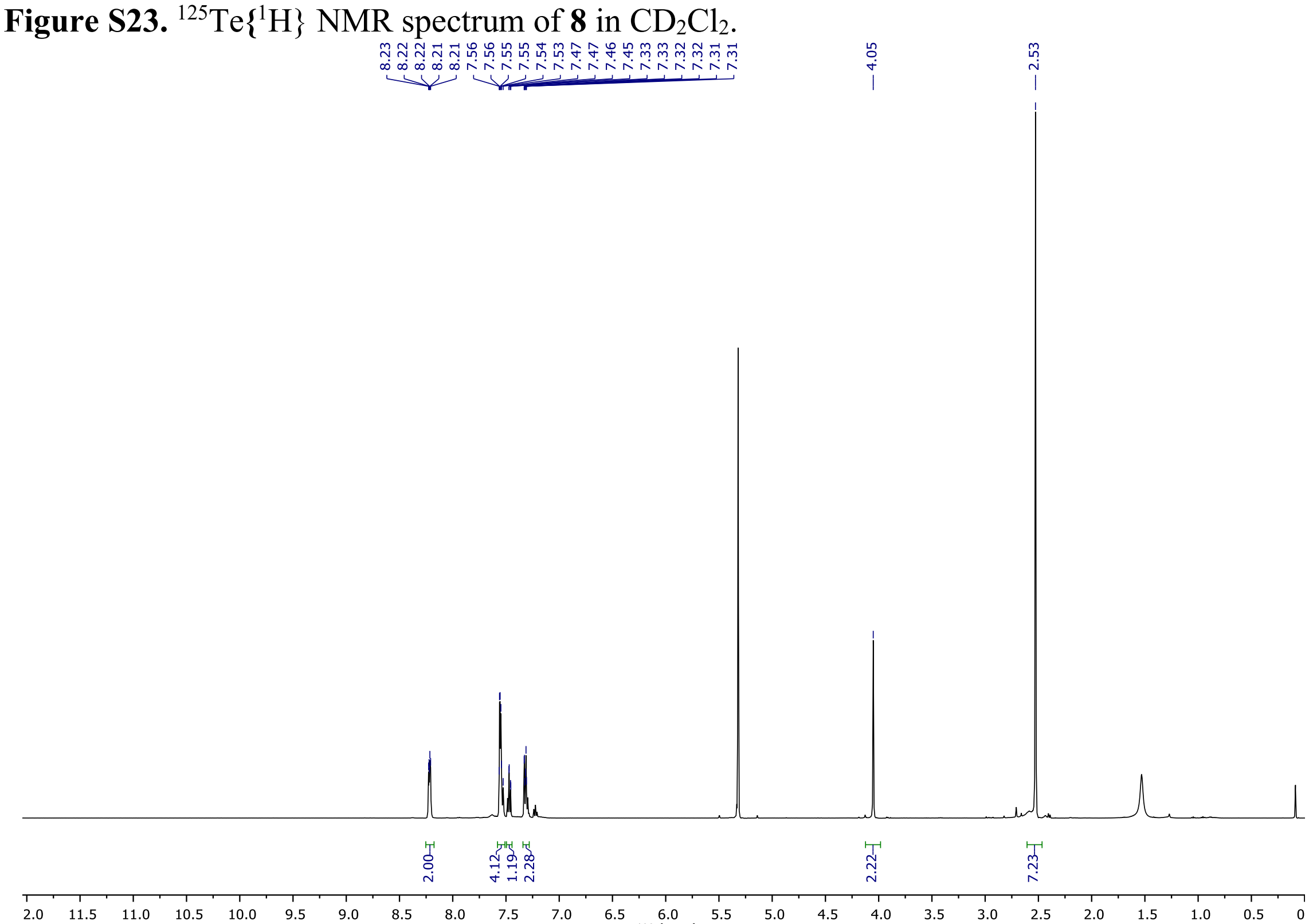

Figure S24. ^{1}H NMR spectrum of **9** in CD_2Cl_2.

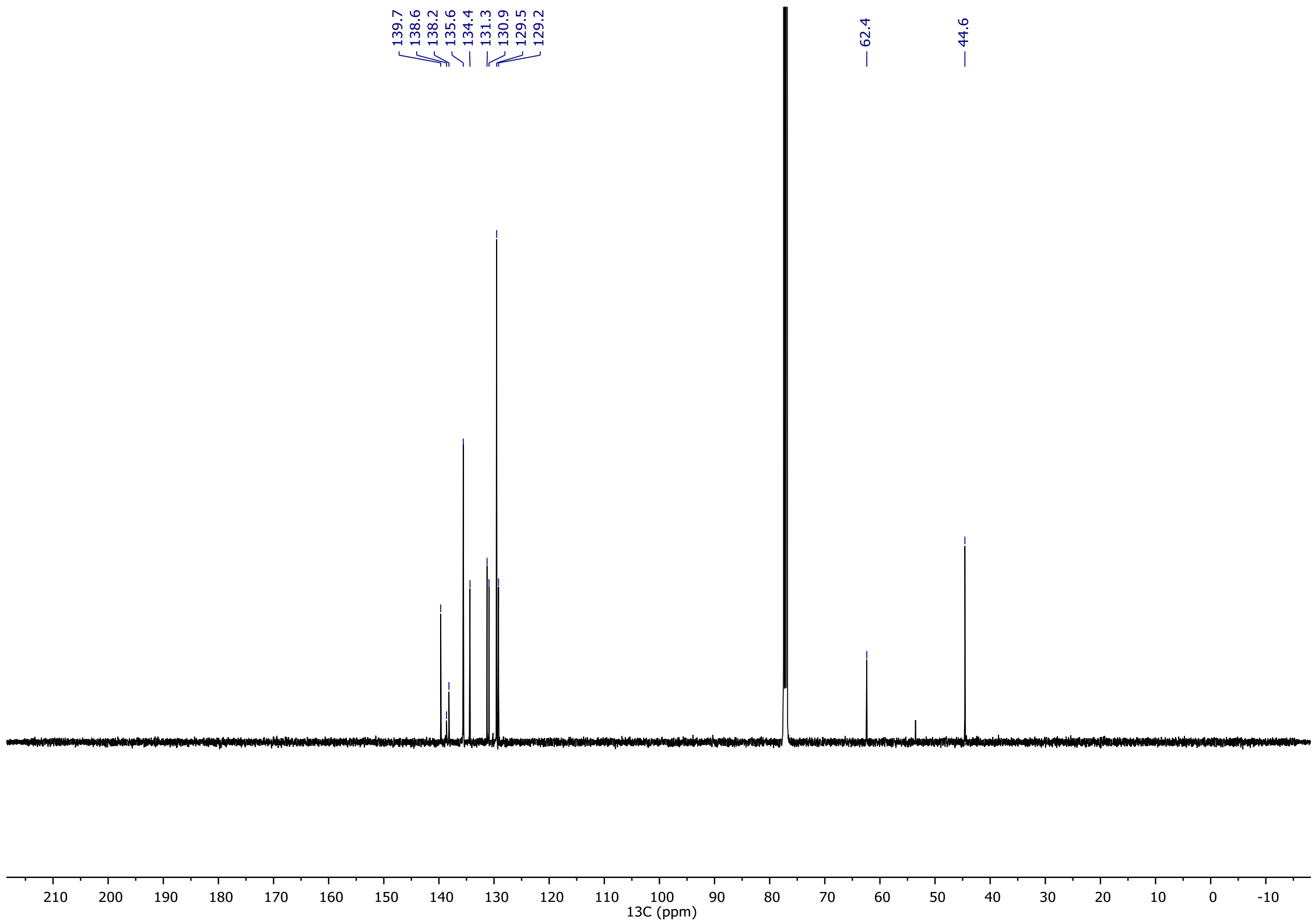

Figure S25. $^{13}C\{^{1}H\}$ NMR spectrum of **9** in $CDCl_3$.

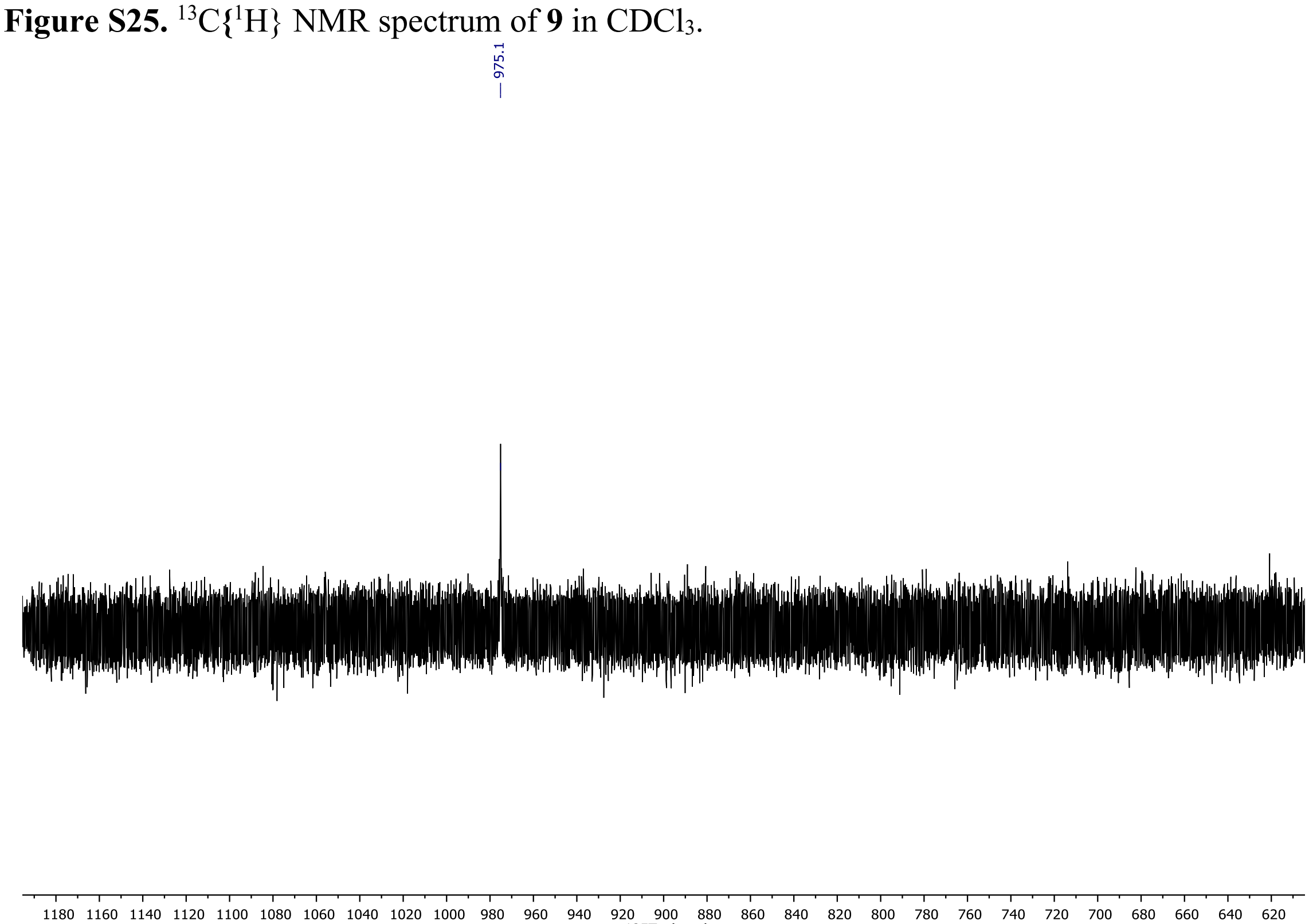

Figure S26. $^{125}Te\{^{1}H\}$ NMR spectrum of **9** in $CDCl_3$.

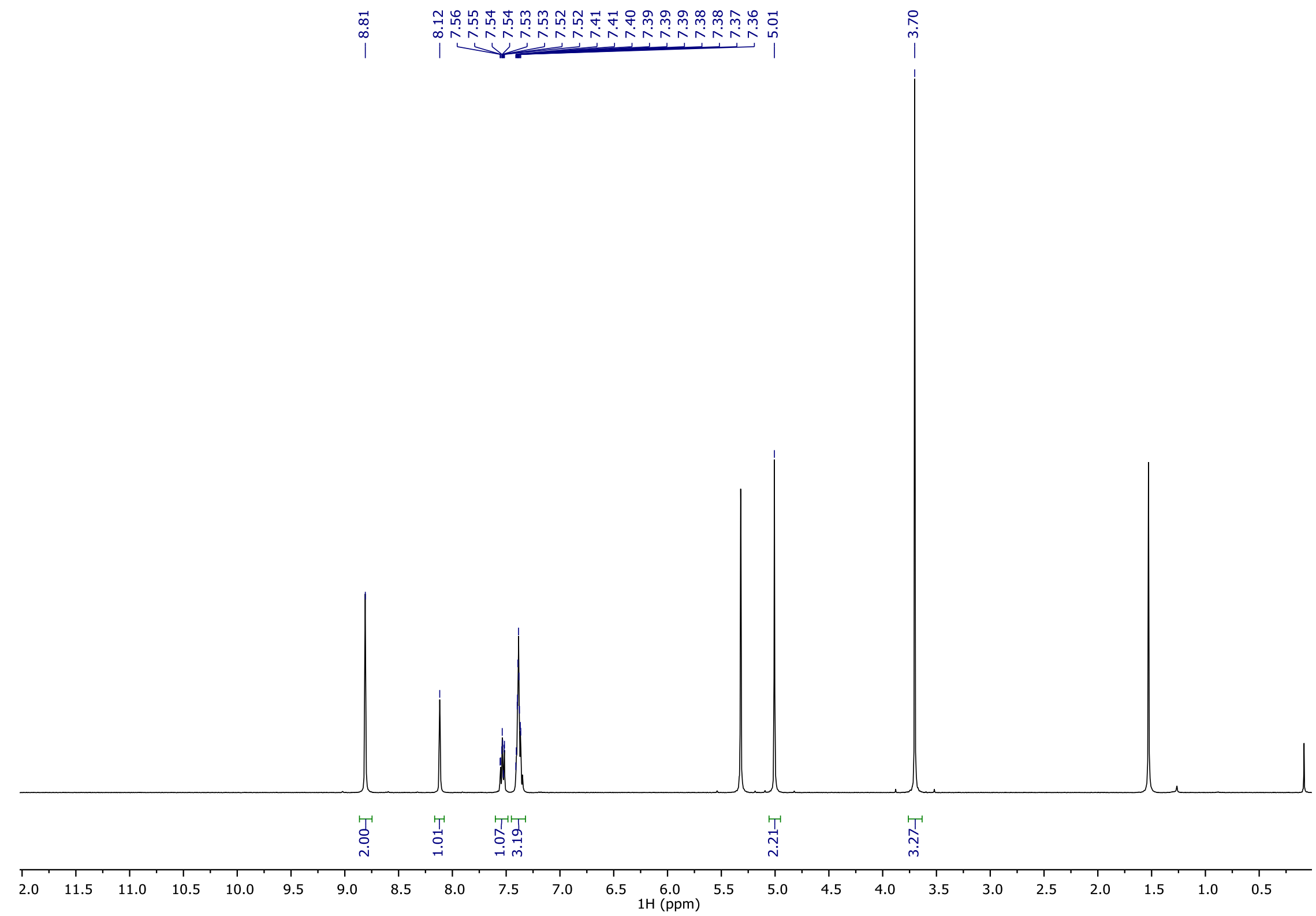

Figure S27. ^{1}H NMR spectrum of **10** in CD_2Cl_2.

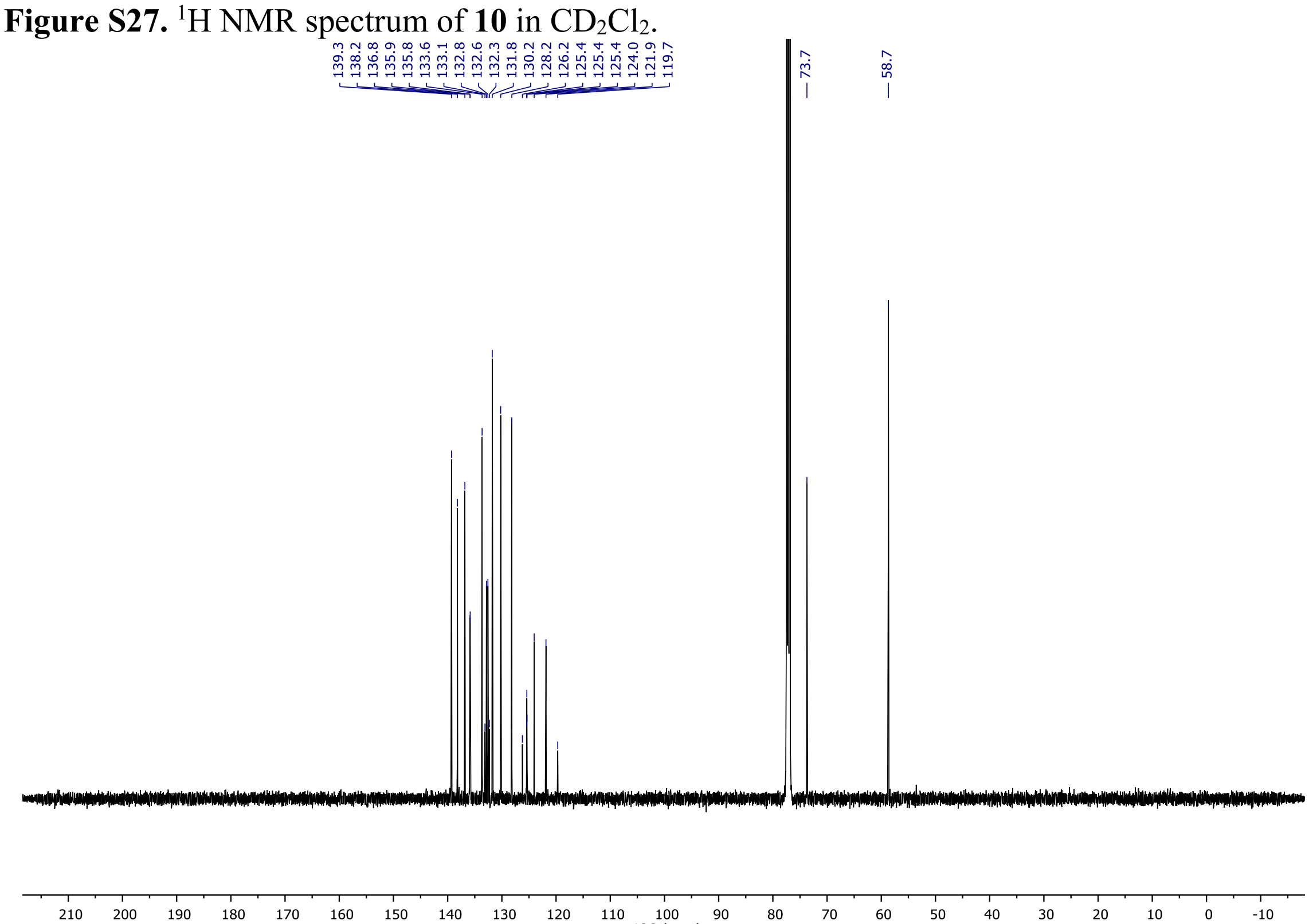

Figure S28. $^{13}C\{^{1}H\}$ NMR spectrum of **10** in $CDCl_3$.

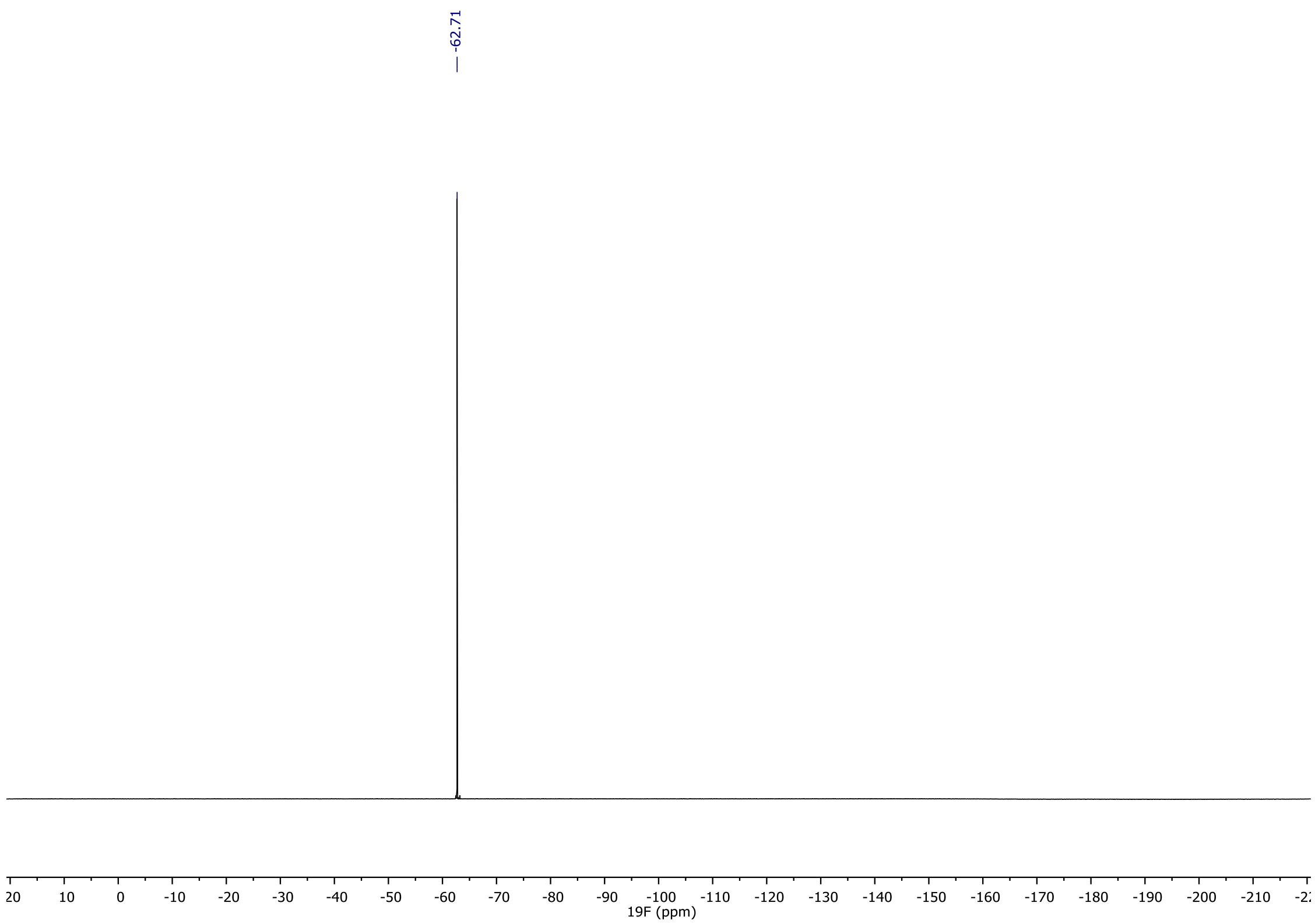

Figure S29. ^{19}F NMR spectrum of **10** in $CDCl_3$.

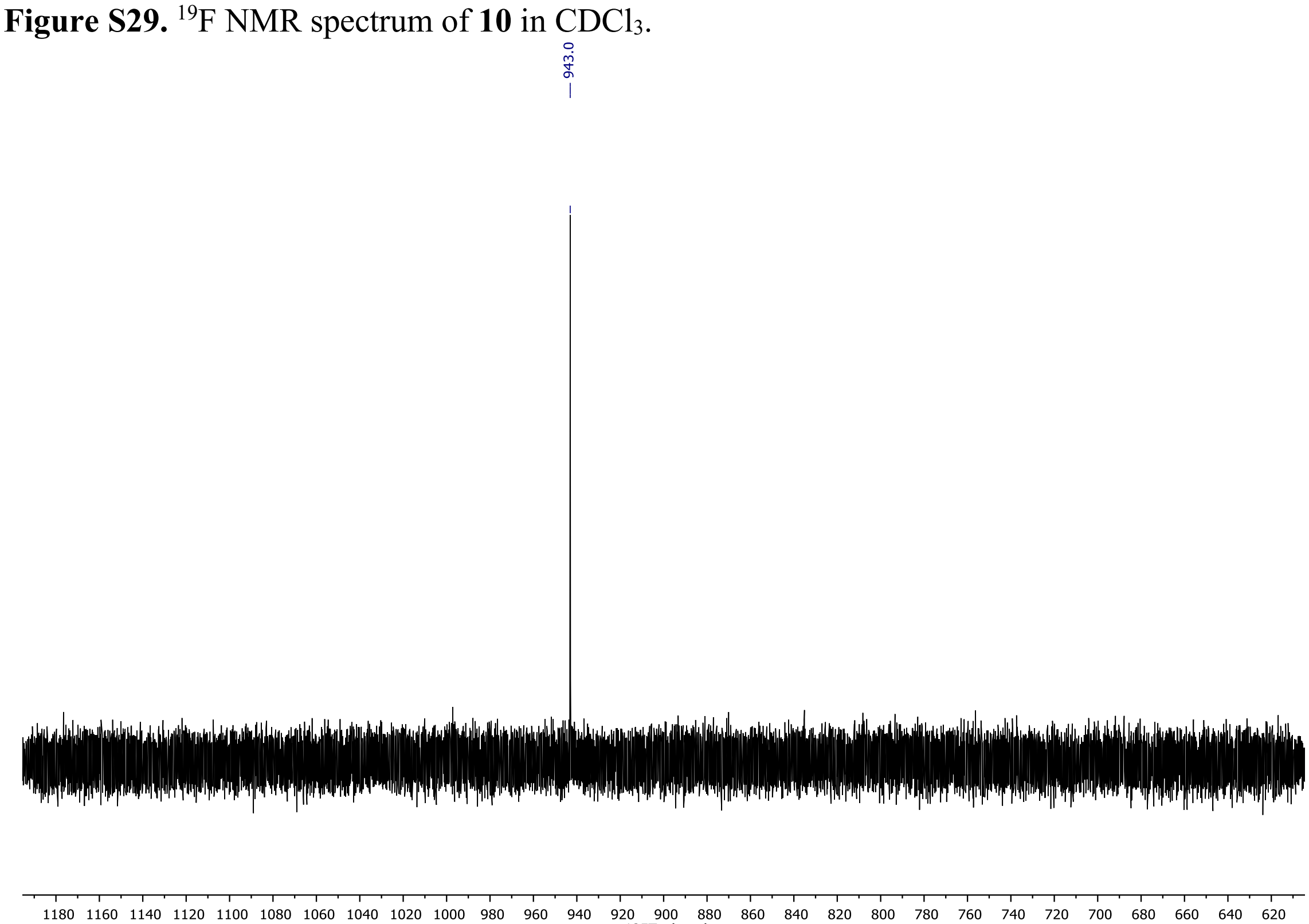

Figure S30. $^{125}Te\{^{1}H\}$ NMR spectrum of **10** in $CDCl_3$.

2 Reaction of Diphenyl Telluriude and *o*-Chloranil

o-Chloranil (1.0 equiv.) was added to a solution of diphenyl telluride (1.0 equiv.) in CH_2Cl_2. The resulting solution was stirred for 5 min. The solvent was then removed, and the residue was analyzed by NMR spectroscopy in $CDCl_3$. The effect of exogenous bases was also tested.

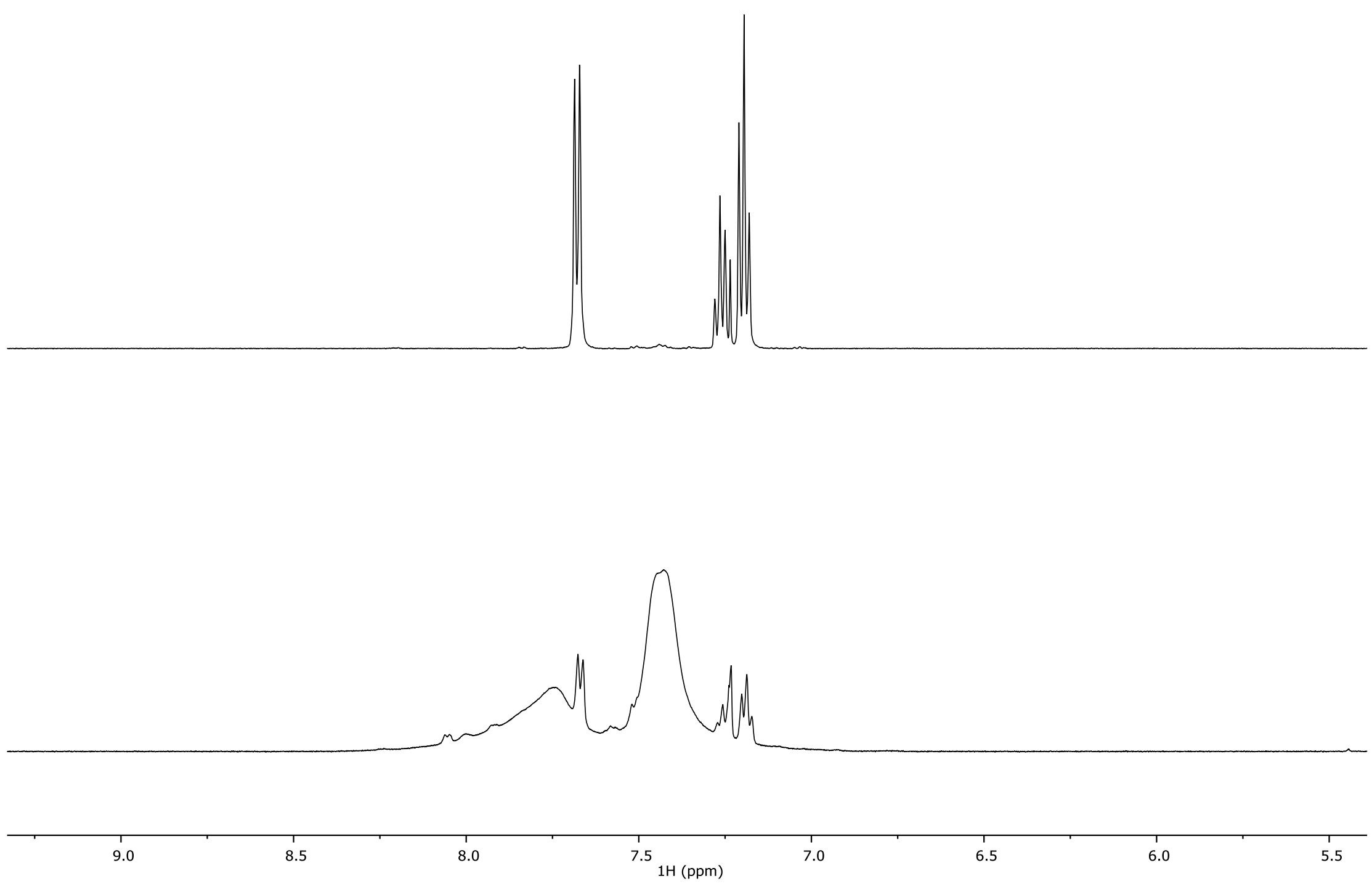

Figure S31. Top: ^{1}H NMR spectrum of diphenyl telluride in $CDCl_3$. Bottom: Reaction of diphenyl telluride and 1.0 equiv. *o*-chloranil in $CDCl_3$ at room temperature.

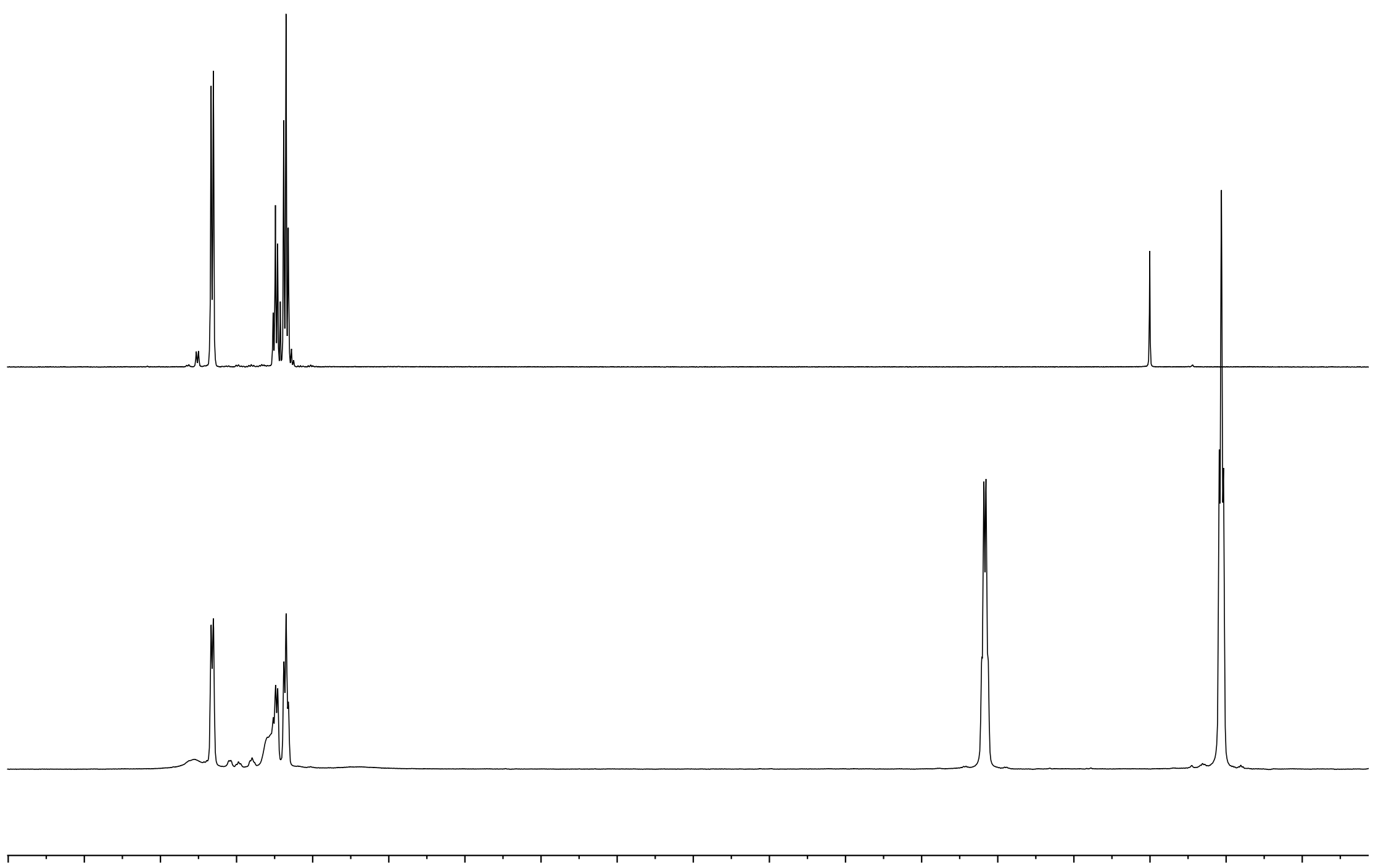

Figure S32. Top: ^{1}H NMR spectrum of diphenyl telluride in $CDCl_3$. Bottom: Addition of excess Et_3N into the reaction of diphenyl telluride and 1.0 equiv. *o*-chloranil in $CDCl_3$ at room temperature.

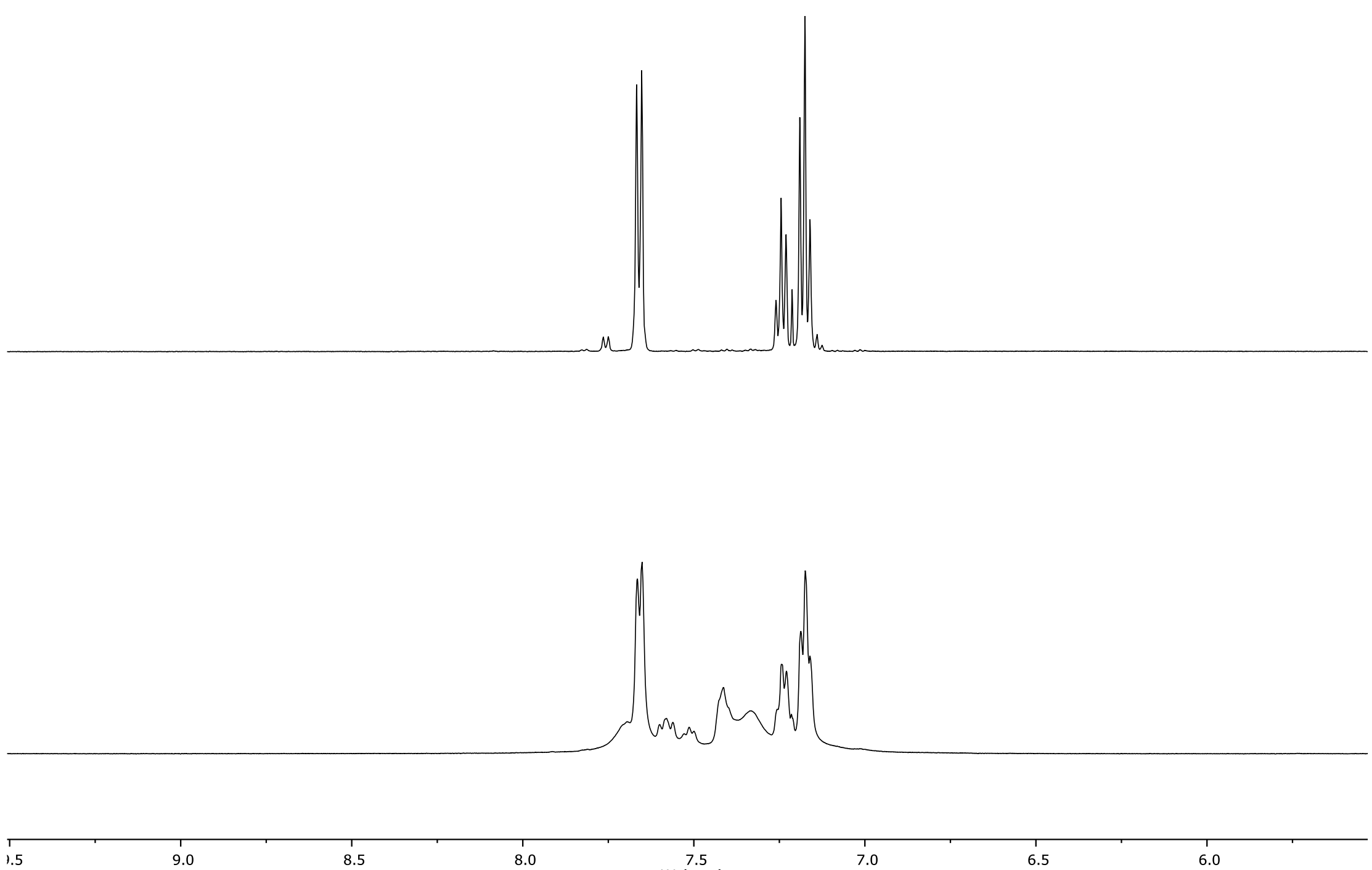

Figure S33. Top: ^{1}H NMR spectrum of diphenyl telluride in $CDCl_3$. Bottom: Addition of excess Ph_3PO into the reaction of diphenyl telluride and 1.0 equiv. *o*-chloranil in $CDCl_3$ at room temperature.

3 Metrical Oxidation State (MOS) of the Catecholate

The Metrical Oxidation State (MOS) values of the catecholate moiety in compounds **4-6** were calculated using the MOS calculator Excel spreadsheet developed by Brown.[4] The table S1 summarizes the C–O and C–C bond lengths within the catecholate framework obtained from the solid-state structures and used for the calculations, along with the corresponding MOS values. Due to the disorder on the catecholate moiety in the solid-state structure of compound **5**, two MOS values were calculated representing partially occupied components, yielding an average value = of -1.76.

Table S1. Summary of bond lengths of catecholate moiety and the calculated MOS values.

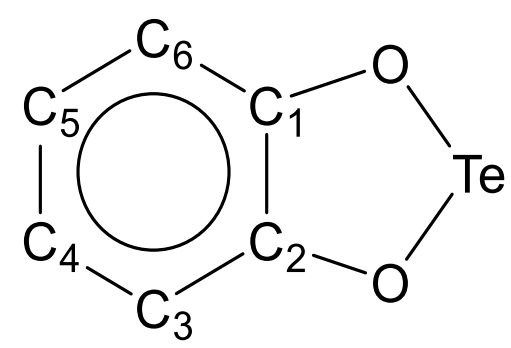

Bond length (Å)	**4**	**5** (Part 1)	**5** (Part 2)	**6**
Avg. of C-O	1.330	1.274	1.387	1.338
C1-C2	1.416	1.431	1.411	1.415
Avg. of C2-C3 & C1-C6	1.388	1.381	1.408	1.382
Avg. of C3-C4 & C6-C5	1.396	1.408	1.385	1.393
C4-C5	1.390	1.387	1.381	1.385
MOS	-1.78(08)	-1.37(29)	-2.14(17)	-1.87(08)

4 Reaction of Diaryl Tellurium Compounds and *o*-Chloranil

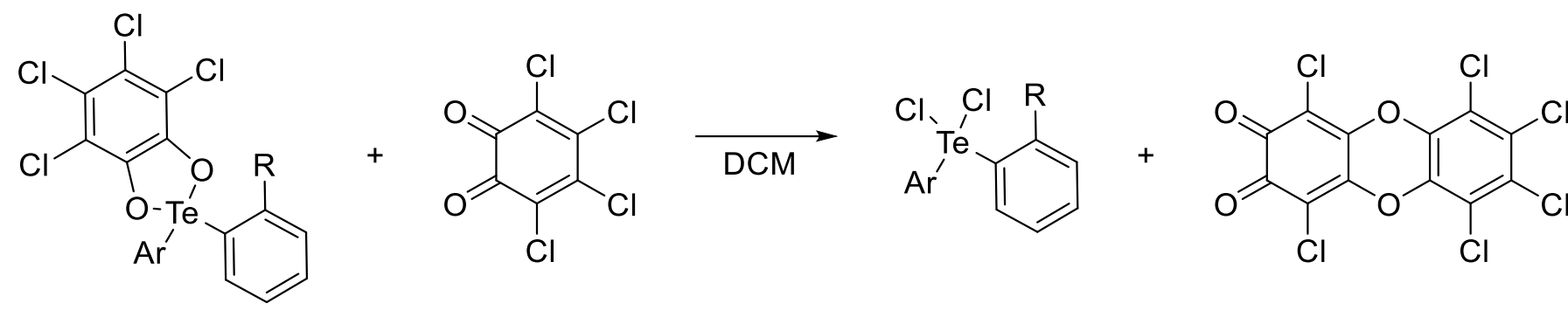

o-Chloranil (1.0 equiv.) was added to a solution of the diaryl tellurium catecholate compound (1.0 equiv.) in CD_2Cl_2. The resulting solution was stirred for 5 min to yield the corresponding diaryl tellurium dichloride compound and dioxin **7**. The formation of diaryl tellurium dichloride compounds was verified by NMR spectroscopy and +ESI mass spectrometry. The formation of dioxin **7** was verified by NMR spectroscopy and X-ray solid state structure.

4.1 NMR Spectra

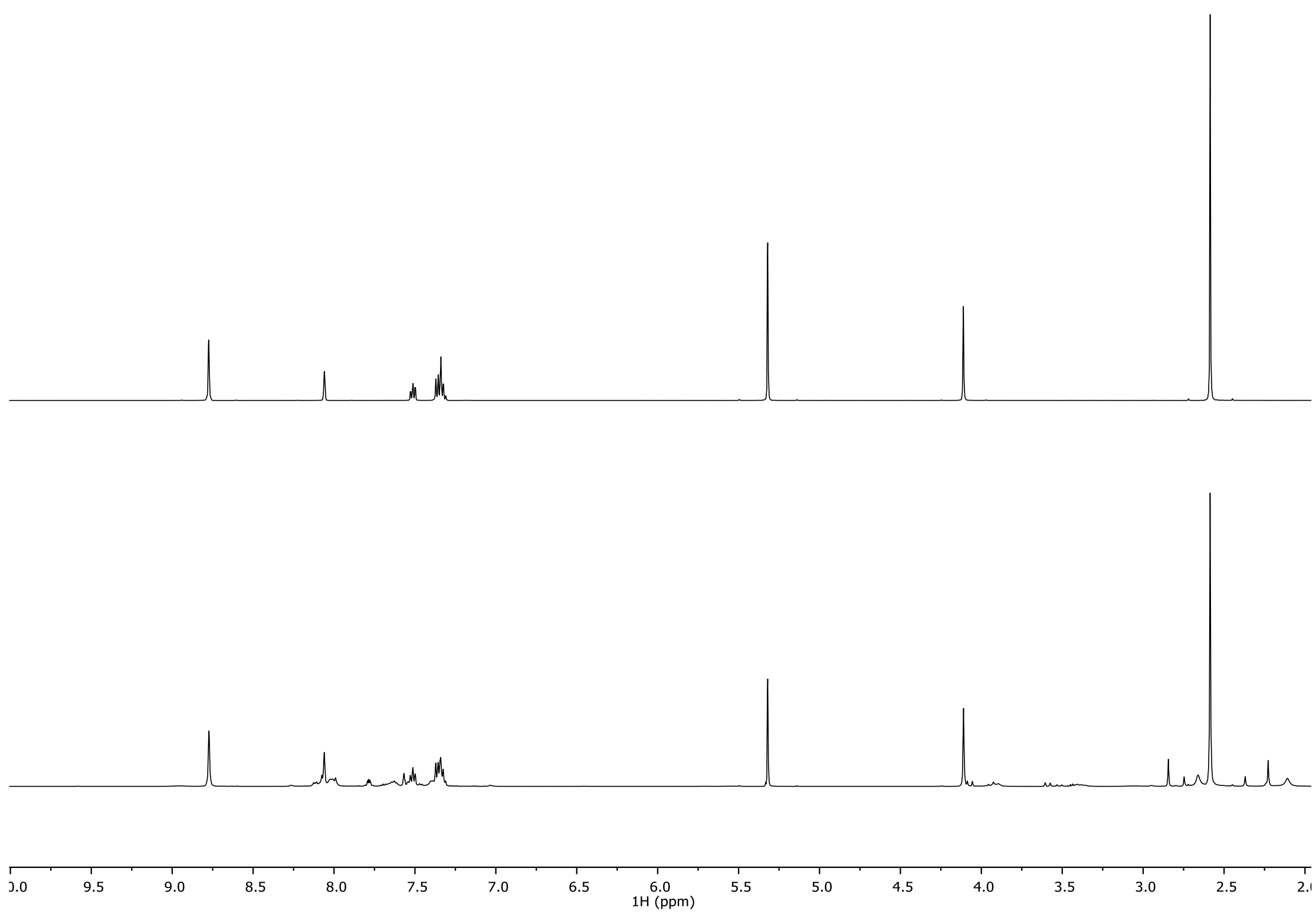

Figure S34. Top: ^{1}H NMR spectrum of compound **8** in CD_2Cl_2. Bottom: Reaction of compound **4** and 1.0 equiv. *o*-chloranil in CD_2Cl_2 at room temperature.

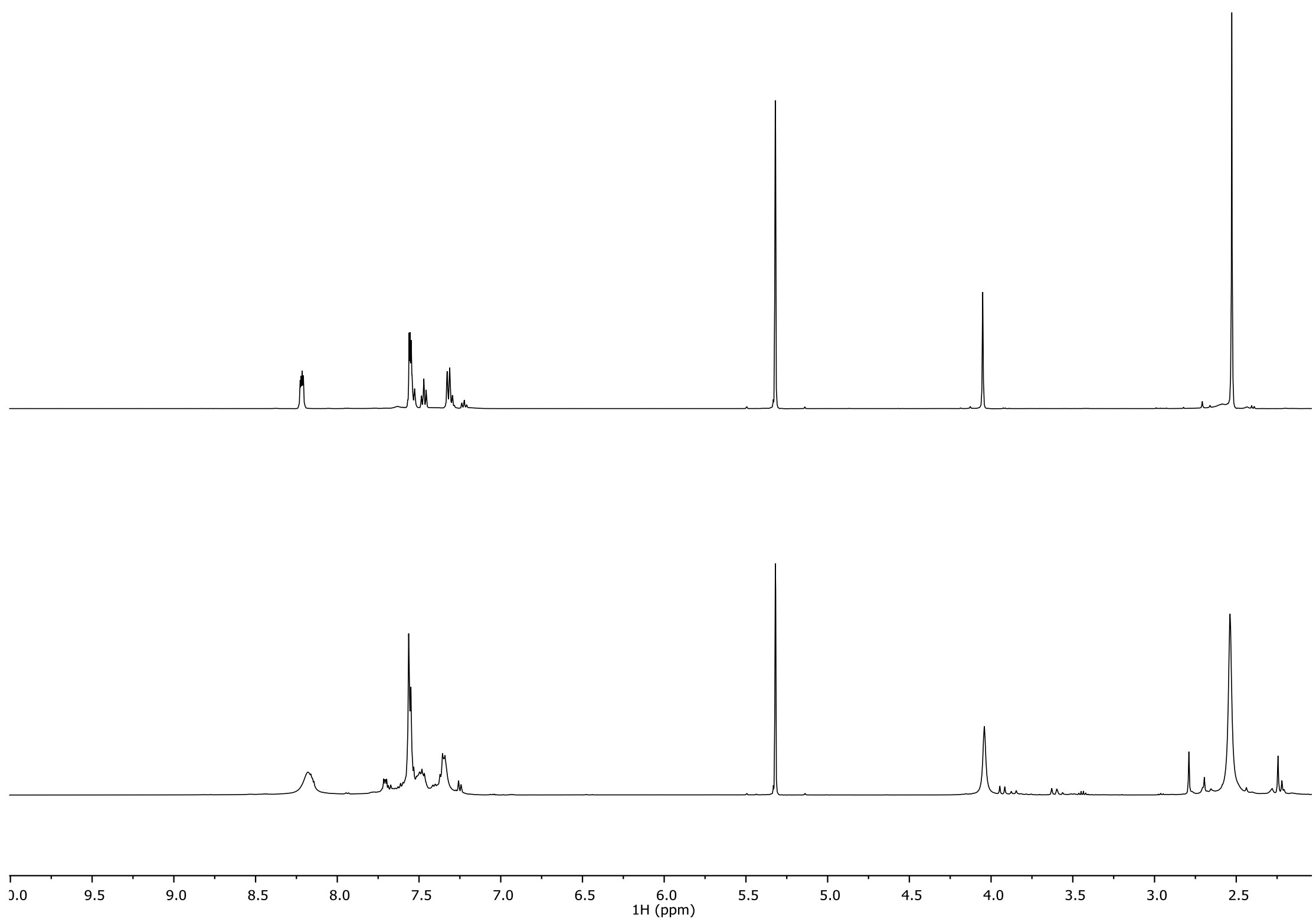

Figure S35. Top: ^{1}H NMR spectrum of compound **9** in CD_2Cl_2. Bottom: Reaction of compound **5** and 1.0 equiv. *o*-chloranil in CD_2Cl_2 at room temperature.

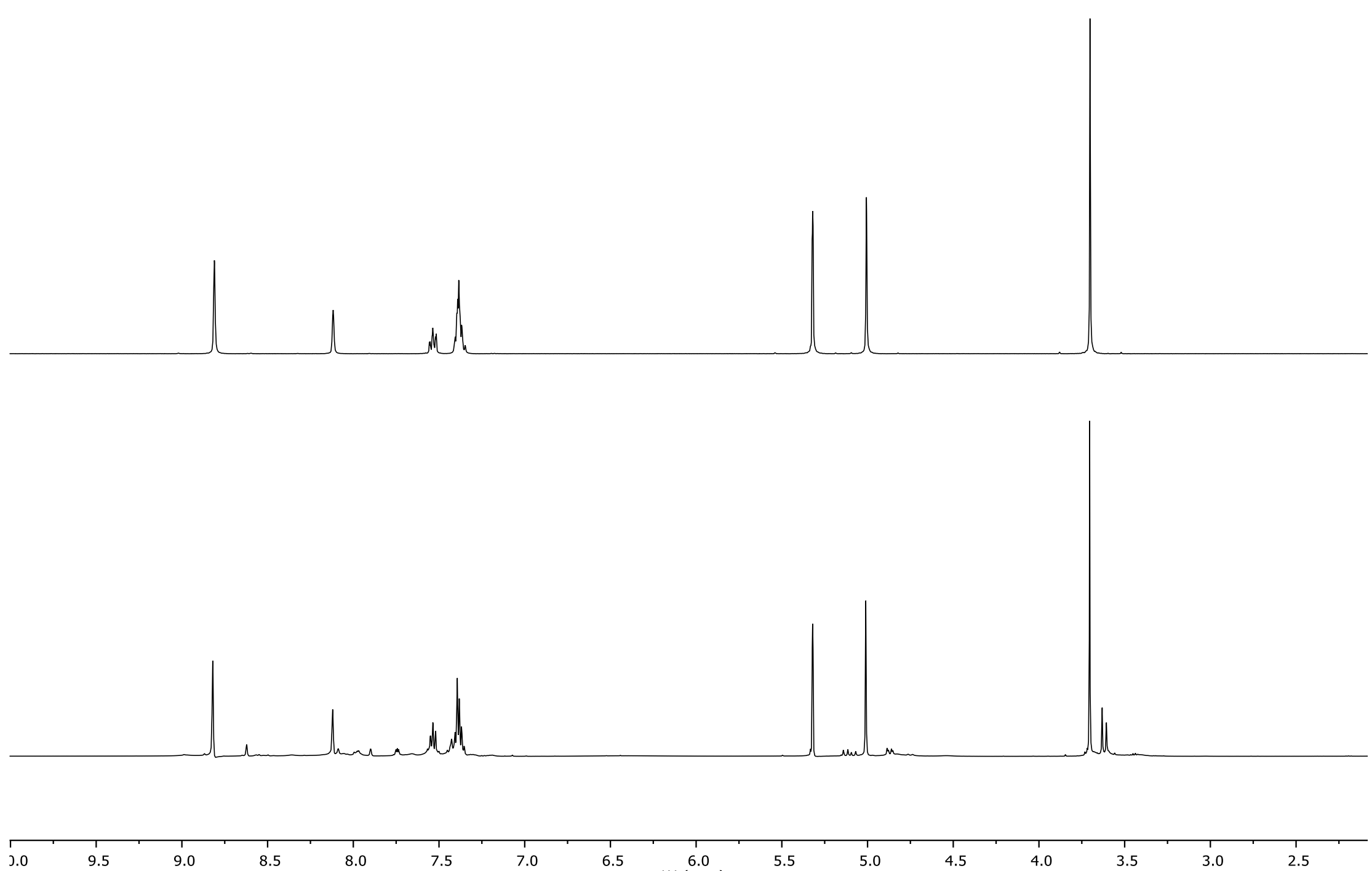

Figure S36. Top: ^{1}H NMR spectrum of compound **10** in CD_2Cl_2. Bottom: Reaction of compound **6** and 1.0 equiv. *o*-chloranil in CD_2Cl_2 at room temperature.

4.2 Mass Spectra

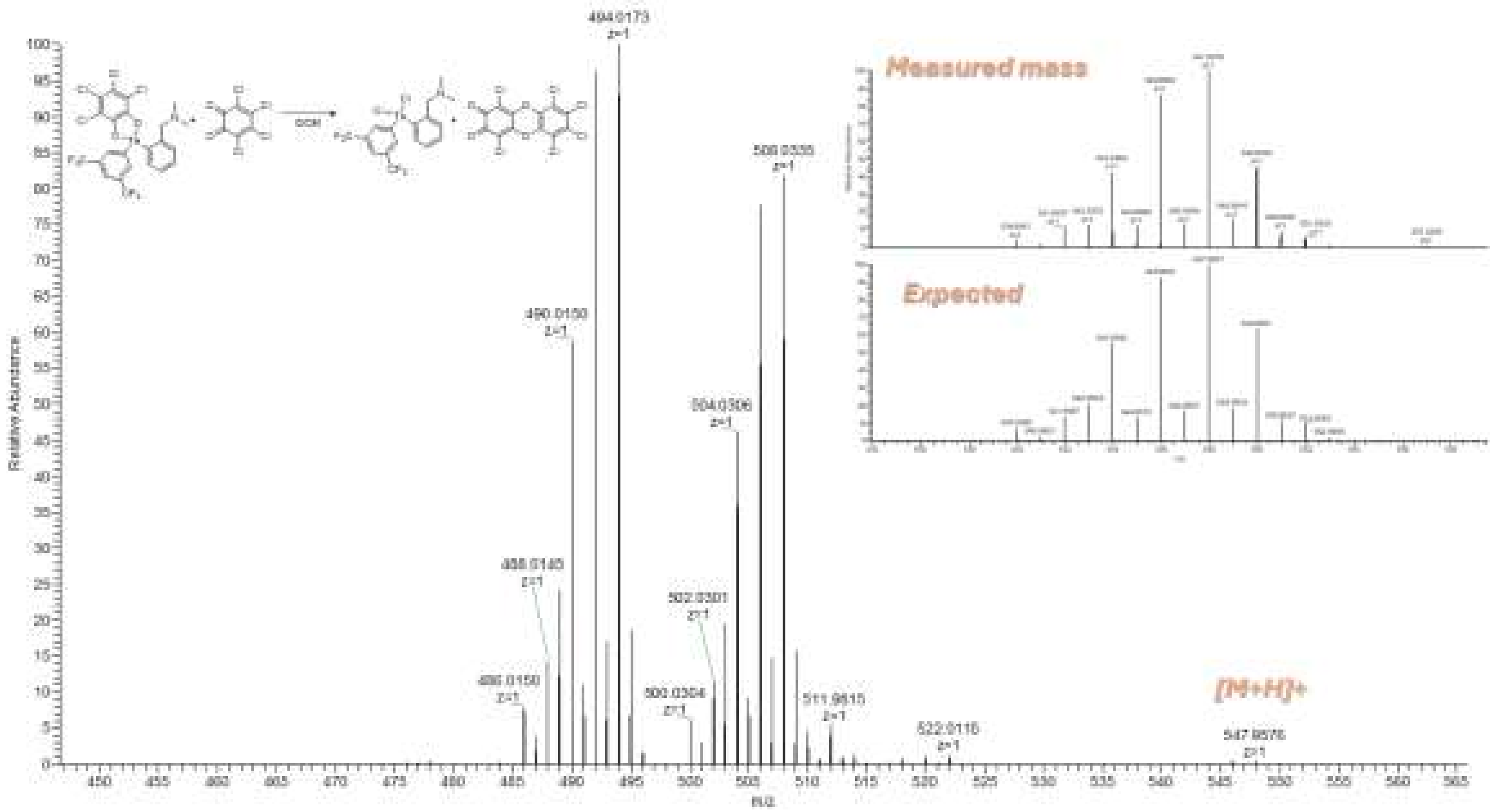

Figure S37. Mass spectrum of a mixture of compound **4** and 1.0 equiv. *o*-chloranil in CD_2Cl_2 at room temperature. Inset: Simulated mass spectrum of $[M+H]^+$ for compound **8**.

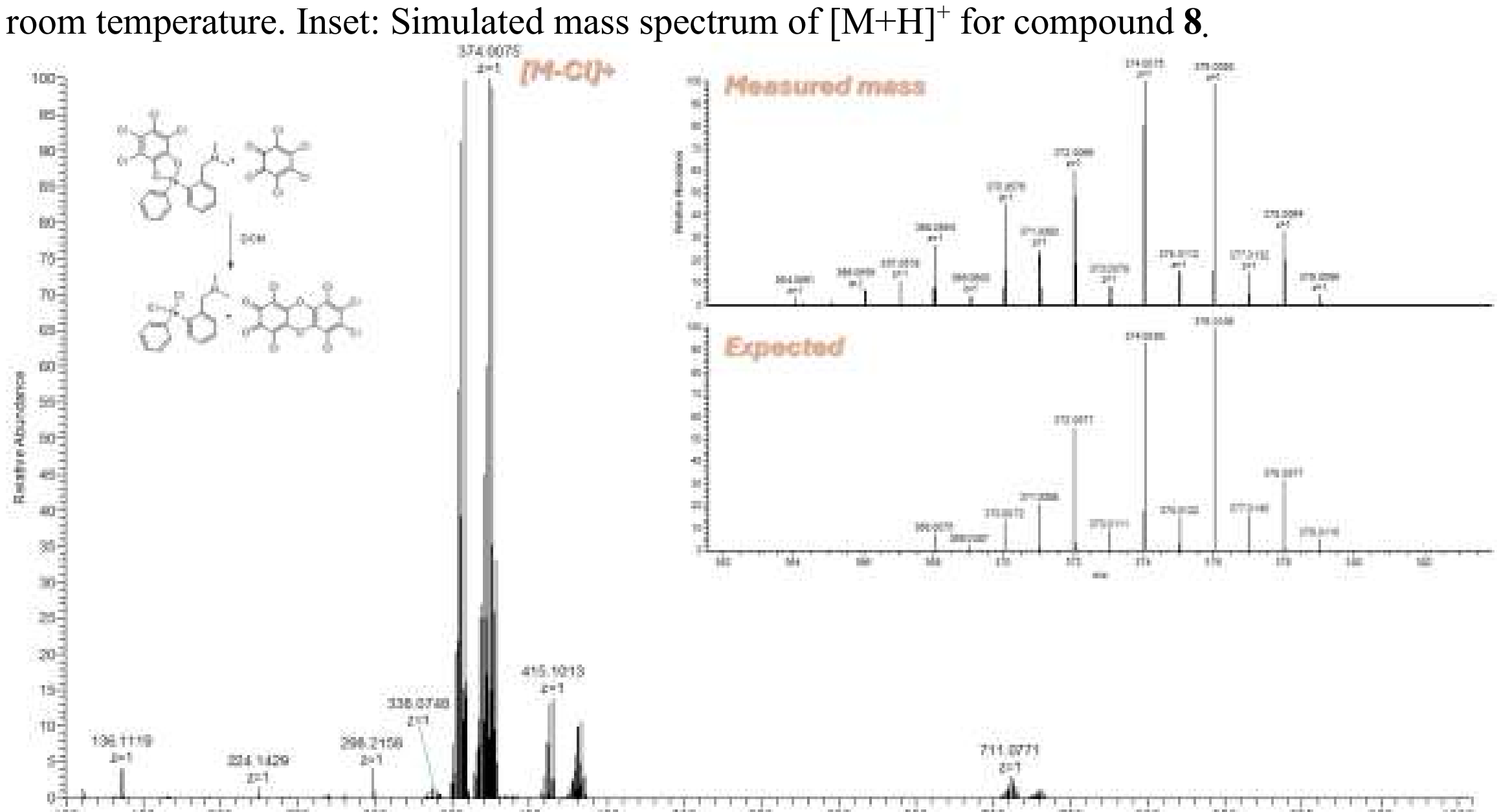

Figure S38. Mass spectrum of mixture of compound **5** and 1.0 equiv. *o*-chloranil in CD_2Cl_2 at room temperature. Inset: Simulated mass spectrum of $[M-Cl]^+$ for compound **9**.

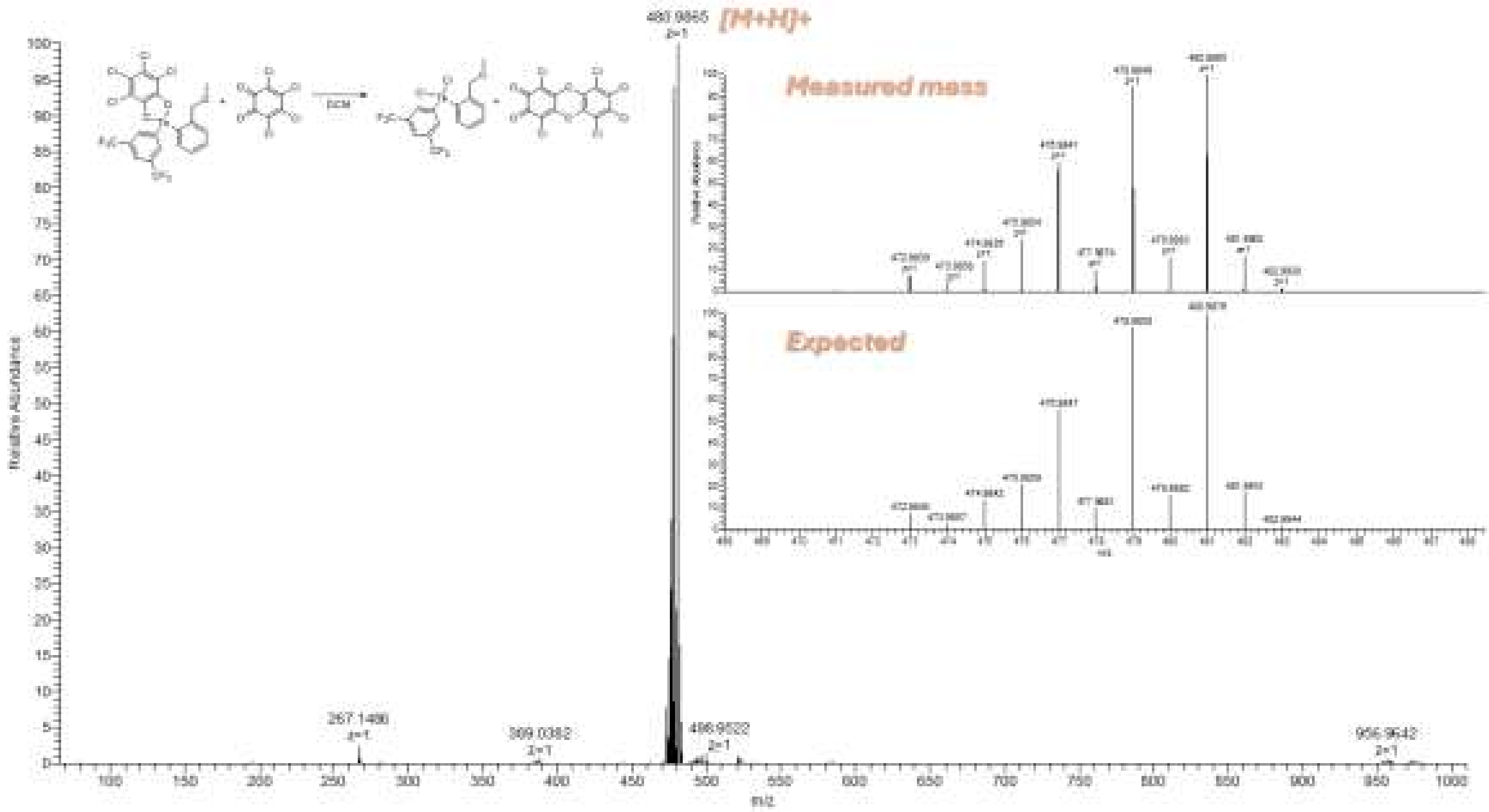

Figure S39. Mass spectrum of mixture of compound **6** and 1.0 equiv. *o*-chloranil in CD_2Cl_2 at room temperature. Inset: Simulated mass spectrum of $[M+H]^+$ for compound **10**.

4.3 Large Scale Reaction of Diaryl Tellurium(II) Compounds and *o*-Chloranil

o-Chloranil (100 mg, 0.4 mmol, 2.0 equiv.) was added to a solution of the diaryl tellurium(II) compound (0.2 mmol, 1.0 equiv.) in CH_2Cl_2. The resulting solution was stirred overnight. A red solid precipitated from the solution. This solid was isolated by filtration, dried under vacuum, and identified as dioxin **7**. The dioxin product yields obtained using the different diaryl tellurium(II) compounds are provided in Table S2.

Table S2. Yields of dioxin in the reaction of diaryl tellurium(II) compounds **1**-**3** with 2.0 equiv. *o*-chloranil.

Compounds	Yield (%)
**1**	77
**2**	23
**3**	48

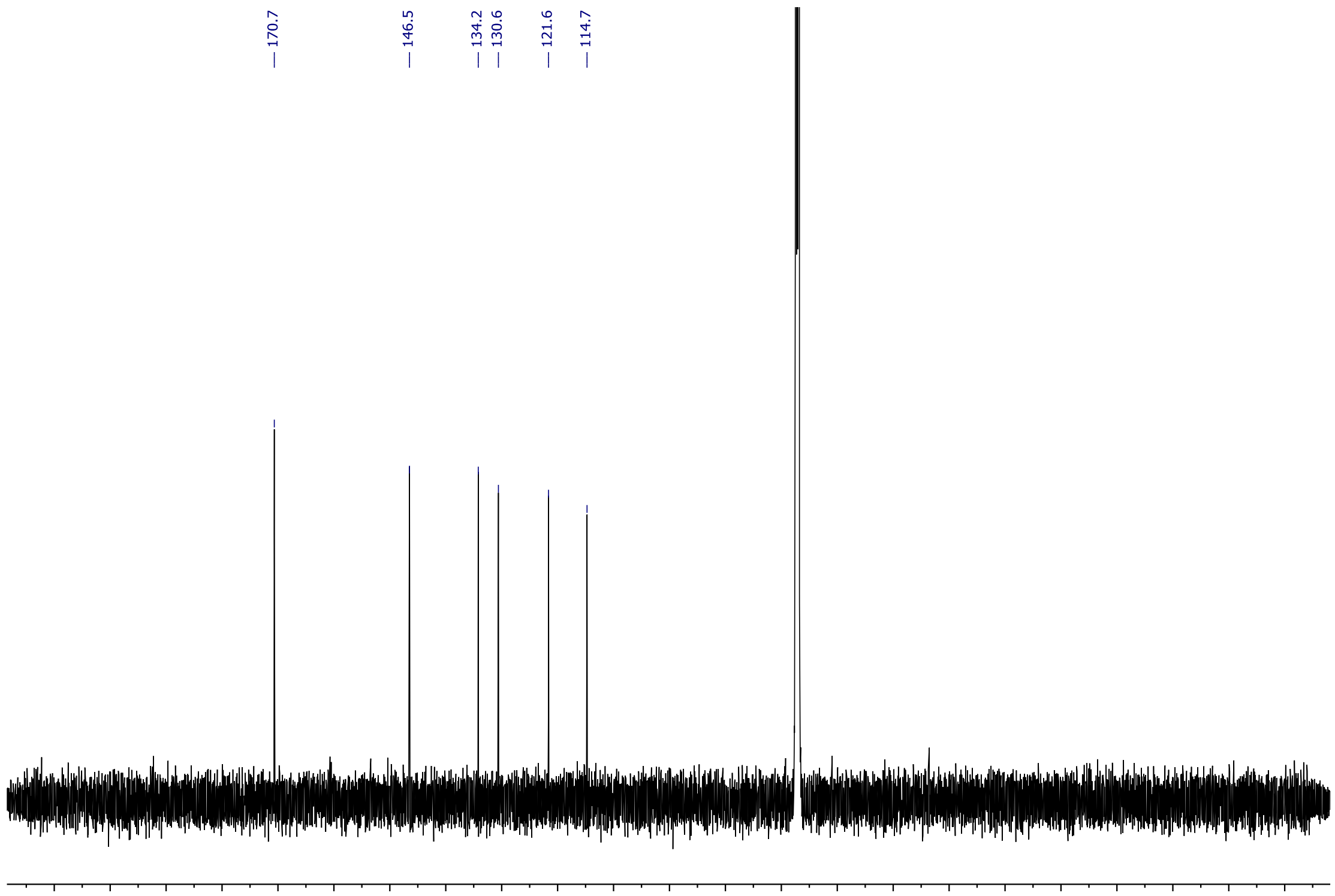

Figure S40. ^{13}C NMR spectrum of the isolated product, featuring the formation of the dioxin **7**.

5 Reduction of diaryl tellurium dichlorides

The reduction of diaryl tellurium dichloride derivatives to the corresponding diaryl tellurium (II) compounds can be achieved by using excess dithiothreitol (DTT). In the case of compounds **8** and **9**, Et_3N was required to prevent protonation of the amine functionality. The reactions were monitored by ^1H NMR in $CDCl_3$. The corresponding spectra are provided below. For **10**, the spectra obtained with other reductants, including glutathione (GSH), Na_2SO_3 and Hantzsch ester are also provided.

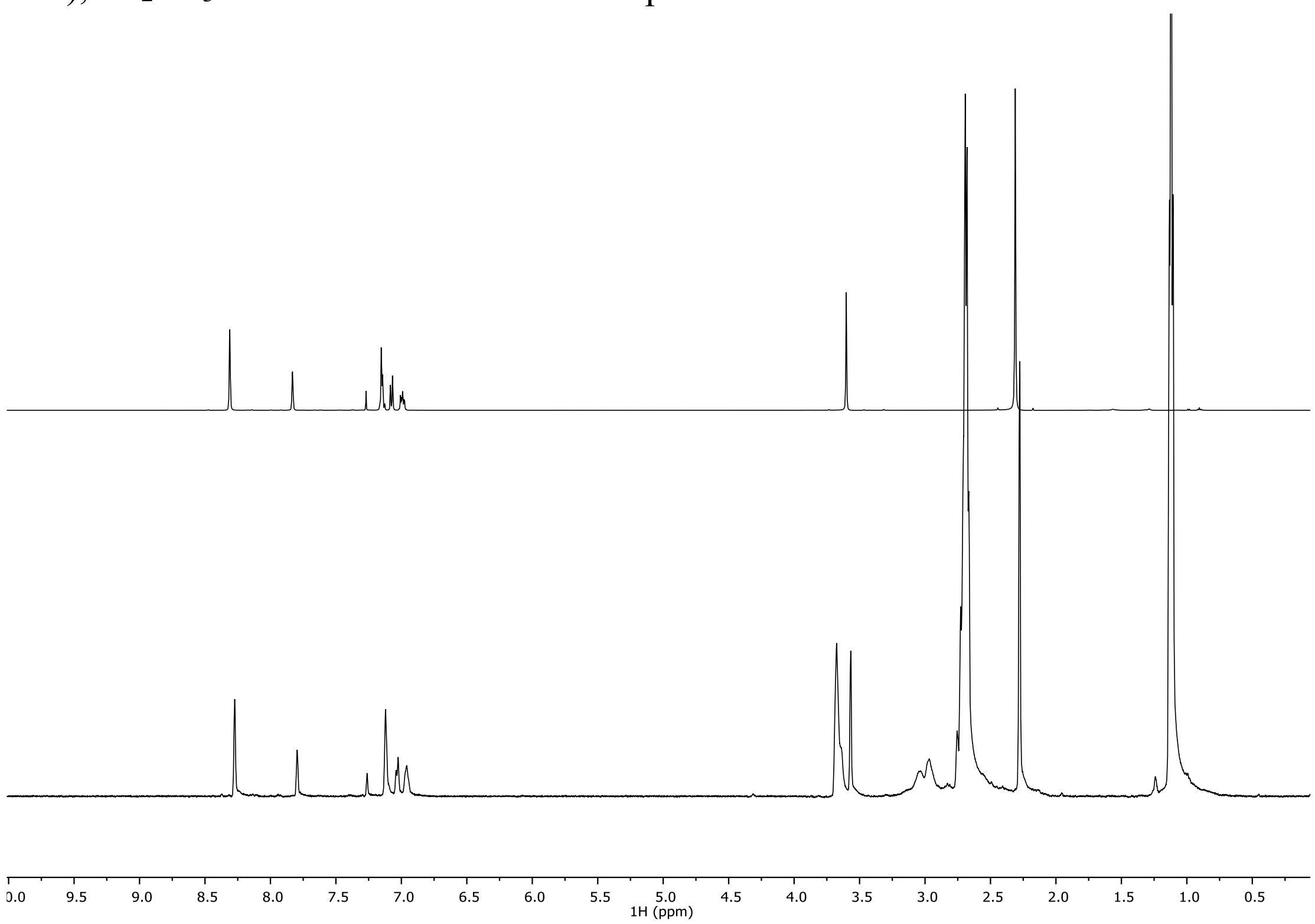

Figure S41. ^1H NMR spectra of compound **1** (top) and compound **8** with excess DTT and Et_3N (bottom). All spectra are recorded in $CDCl_3$.

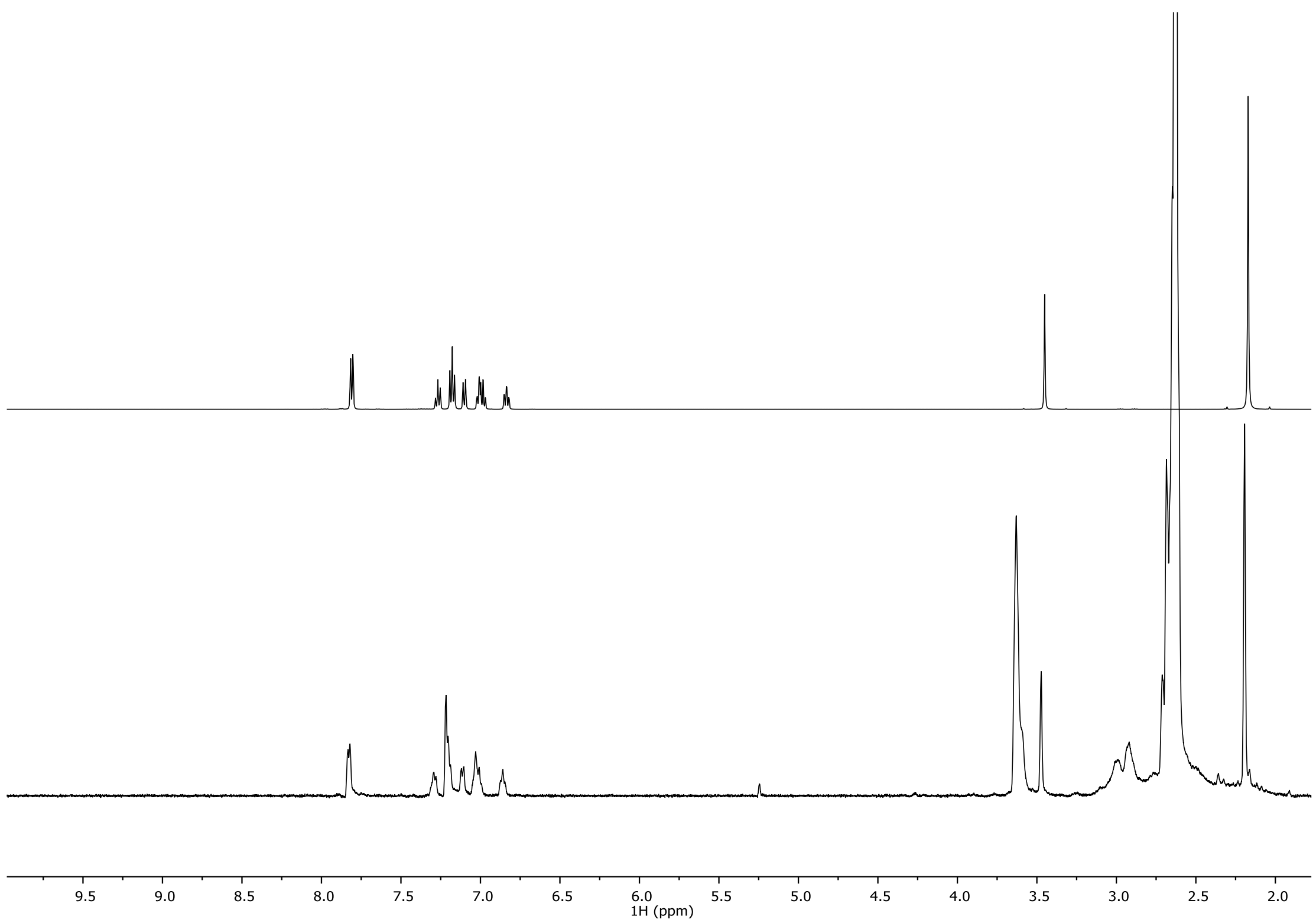

Figure S42. ^{1}H NMR spectra of compound **2** (top) and compound **9** with excess DTT and Et_3N (bottom). All spectra are recorded in $CDCl_3$.

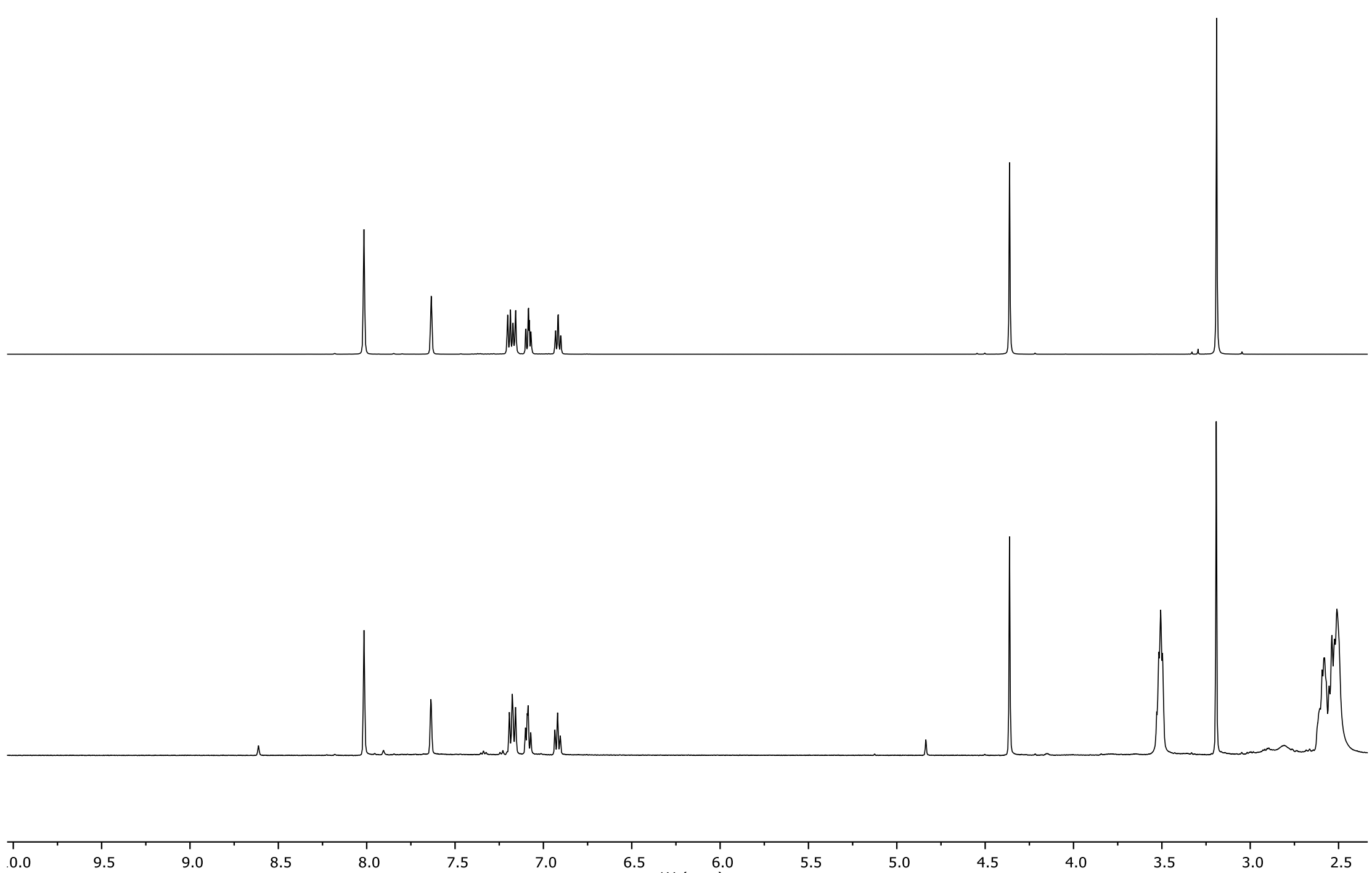

Figure S43. ^{1}H NMR spectra of compound **3** (top) and compound **10** with excess DTT (bottom). All spectra are recorded in $CDCl_3$.

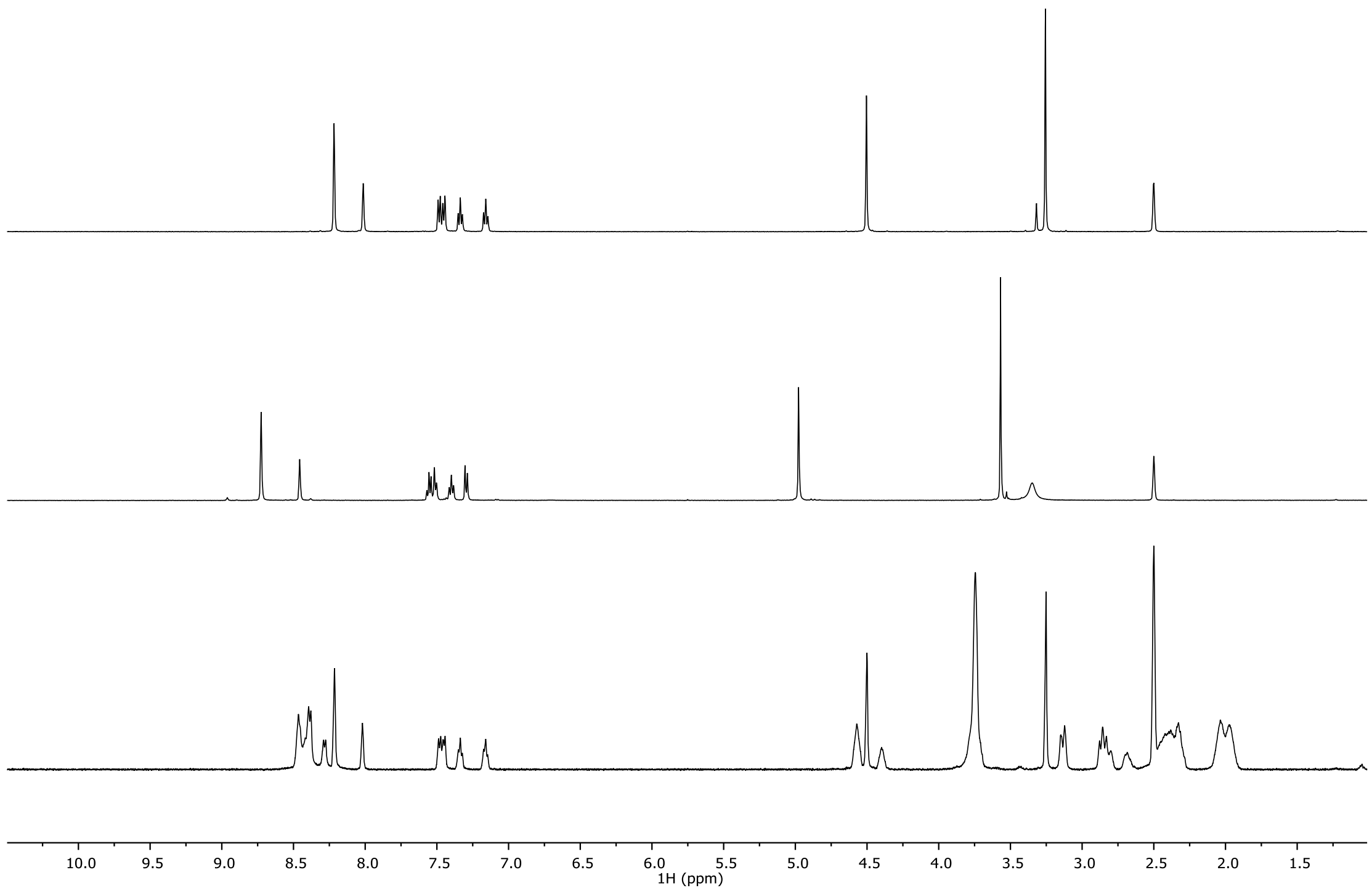

Figure S44. ^{1}H NMR spectra of compound **3** (top), compound **10** (middle) and compound **10** with excess GSH (bottom). All spectra are recorded in $(CD_3)_2SO$.

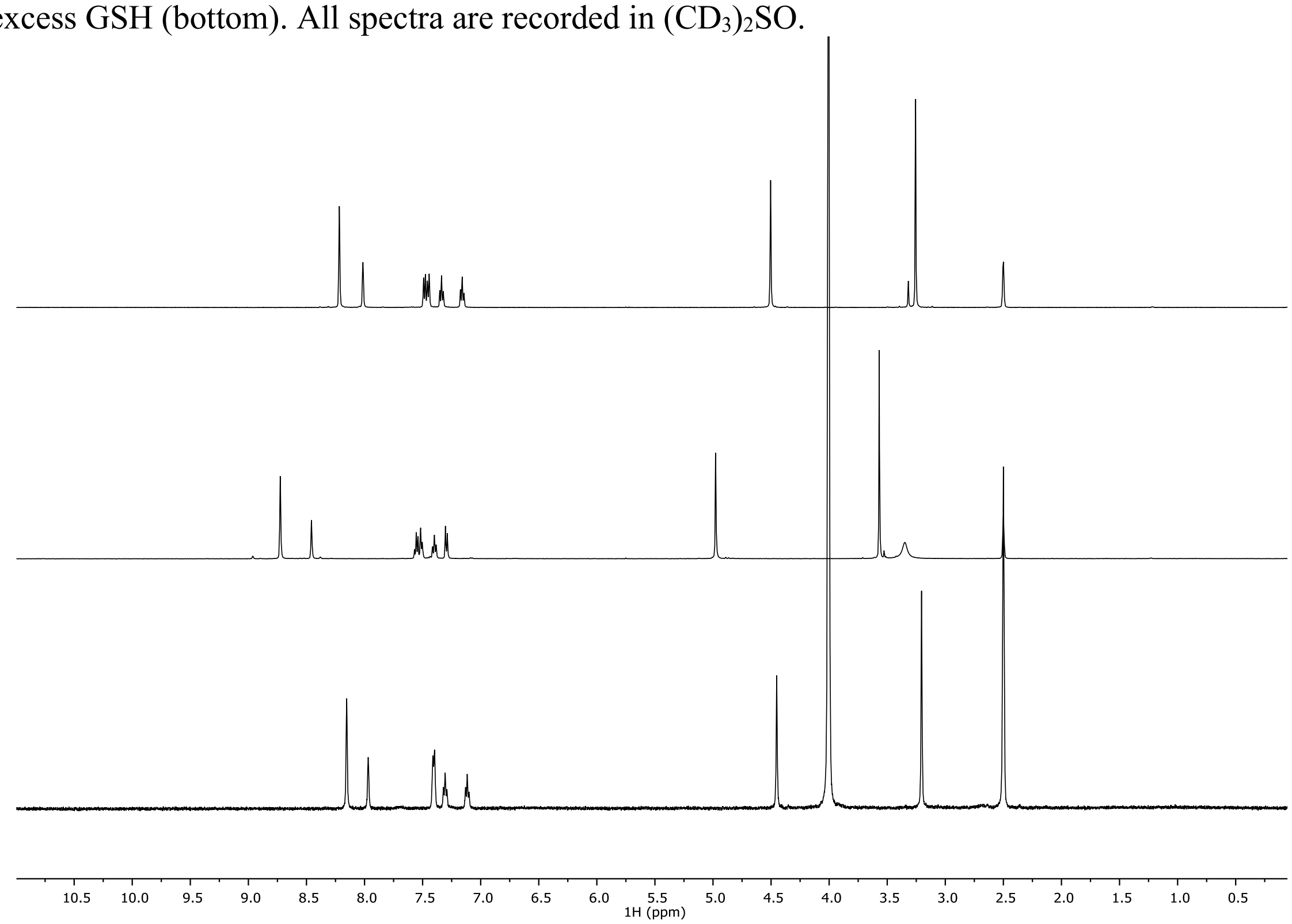

Figure S45. ^{1}H NMR spectra of compound **3** (top), compound **10** (middle) and compound **10** with excess Na_2SO_3 with a small amount of D_2O (bottom). All spectra are recorded in $(CD_3)_2SO$.

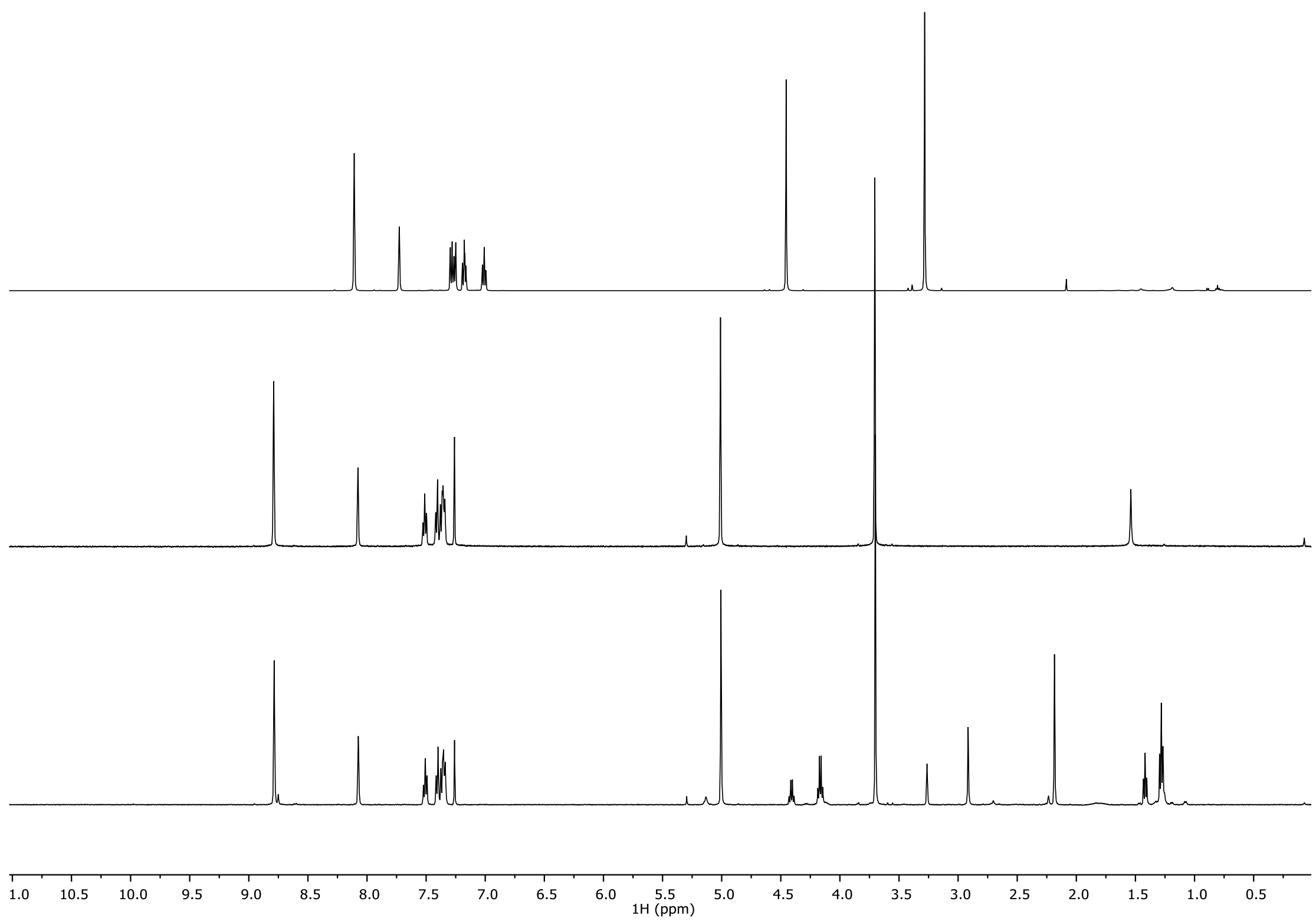

Figure S46. ^{1}H NMR spectra of compound **3** (top), compound **10** (middle) and compound **10** with excess Hantzsch ester (bottom). All spectra are recorded in $CDCl_3$.

6 X-ray Crystallographic Data

The crystallographic measurements were performed at 110 K using a Bruker D8 Quest (Mo source) diffractometer equipped with Photon III detectors or a Synergy Rigaku (Cu or Mo source) diffractometer equipped with a HyPix-6000HE detector. Semi-empirical absorption corrections were applied using the Bruker SADABS software package.[5] The structures were solved by direct methods with SHELXT to locate all non-hydrogen atoms.[6] Subsequent refinement using a difference fourier map against F^2 with the SHEXL package allowed for the anisotropic refinement of non-hydrogen atoms. H atoms were added in calculated positions using the riding atom model. Structure refinement was performed on Olex2.[7] CCDC 2554903-2554907 and 2554700 contain the supplementary crystallographic data for this paper. These data can be obtained free of charge via www.ccdc.cam.ac.uk/data_request/cif, or by emailing data_request@ccdc.cam.ac.uk.

7 Computational Methods

7.1 Geometry optimization and energy calculations

All compounds were optimized using Gaussian 16[8] (functional: B3LYP-D3(BJ)[9], basis set: 6-31g(d) for C, H, N, O, F, Cl, and def2-TZVP[10] for Te, with the SMD[11] solvent model of chloroform). All geometries were identified as local minima using frequency calculations at the same level of theory. To obtain enthalpies and Gibbs free energies from these electronic energies, the necessary thermochemical corrections were added from the frequency calculations. The optimized structures were visualized by GaussView 6. NBO analysis was performed using the same functional and basis set with the NBO 7.0 program.[12] The resulting NBOs were visualized using Avogadro.[13]

7.2 Comparison of Isomer Energies

Table S3. Summary of energies of cosmpounds **4-6** in equatorial and axial isomers, and free Gibbs energy (ΔG) difference between these two forms.

Equatorial form (**E**)	**4**	**5**	**6**
E (Hartree)	-3798.73564	-3124.65757	-3779.26443
G_{corr} (Hartree)	0.27070	0.27194	0.22741
G (Hartree)	-3798.46494	-3124.38563	-3779.03702

Axial form (**A**)	**4**	**5**	**6**
E (Hartree)	-3798.73727	-3124.65960	-3779.26597
G_{corr} (Hartree)	0.27017	0.27289	0.22969
G (Hartree)	-3798.46710	-3124.38671	-3779.03628

	**4**	**5**	**6**
$\Delta G_{\mathbf{A-E}}$ (kcal/mol)	-1.4	-0.7	0.5

7.3 Energy Summary

Table S4. Energy data of Te(II), Te(IV) compounds and *o*-chloranil.

	Ph_2Te	Adduct of Ph_2Te and o-chloranil	**1**	**2**
E (Hartree)	-731.45957	-2951.33407	-1578.84875	-904.76838
H_{corr} (Hartree)	0.19341	0.25460	0.31760	0.30101
G_{corr} (Hartree)	0.13955	0.17361	0.23429	0.23698
H (Hartree)	-731.26616	-2951.07947	-1578.53115	-904.46737
G (Hartree)	-731.32002	-2951.16046	-1578.61446	-904.53140

	**3**	**4**	**5**	**6**
E (Hartree)	-1559.38541	-3798.73564	-3124.65960	-3779.26443
H_{corr} (Hartree)	0.27526	0.37827	0.36280	0.33669
G_{corr} (Hartree)	0.19375	0.27070	0.27289	0.22741
H (Hartree)	-1559.11015	-3798.35737	-3124.29680	-3778.92774
G (Hartree)	-1559.19166	-3798.46494	-3124.38671	-3779.03702

	o-chloranil
E (Hartree)	-2219.84969
H_{corr} (Hartree)	0.05887
G_{corr} (Hartree)	0.00736
H (Hartree)	-2219.79081
G (Hartree)	-2219.84232

References

(1) Weiss, R.; Aubert, E.; Pale, P.; Mamane, V. Chalcogen-Bonding Catalysis with Telluronium Cations. *Angew. Chem. Int. Ed.* **2021**, *60*, 19281–19286, DOI: 10.1002/anie.202105482.
(2) Alberto, E. E.; Muller, L. M.; Detty, M. R. Rate Accelerations of Bromination Reactions with NaBr and H_2O_2 via the Addition of Catalytic Quantities of Diaryl Ditellurides. *Organometallics* **2014**, *33*, 5571–5581, DOI: 10.1021/om500883f.
(3) Gupta, A.; Deka, R.; Raju, S.; Singh, H. B.; Butcher, R. J. Synthesis of intramolecularly coordinated heteroleptic diorganotellurides and diorganotelluroxides: Isolation of monomeric diorganotelluroxide $[\{2,6\text{-}(Me_2NCH_2)_2C_6H_3\}_2TeO]$ and diorganohydroxytelluronium chloride $[\{2,6\text{-}(Me_2NCH_2)_2C_6H_3\}_2Te(OH)]Cl$. *J. Organomet. Chem.* **2019**, *894*, 10–17, DOI: 10.1016/j.jorganchem.2019.05.003.
(4) Brown, S. N. Metrical Oxidation States of 2-Amidophenoxide and Catecholate Ligands: Structural Signatures of Metal–Ligand π Bonding in Potentially Noninnocent Ligands. *Inorganic Chemistry* **2012**, *51*, 1251–1260, DOI: 10.1021/ic202764j.
(5) *SADABS.*; University of Göttingen, Germany, 2016.
(6) *SHELXL-2014: Program for Crystal Structure Refinement*; University of Göttingen, Germany, 2014.
(7) Dolomanov, O. V.; Bourhis, L. J.; Gildea, R. J.; Howard, J. A. K.; Puschmann, H. OLEX2: a complete structure solution, refinement and analysis program. *J. Appl. Crystallogr.* **2009**, *42*, 339–341, DOI: 10.1107/S0021889808042726.
(8) *Gaussian 16 Rev. C.01*; Wallingford, CT, 2016.
(9) Grimme, S.; Ehrlich, S.; Goerigk, L. Effect of the damping function in dispersion corrected density functional theory. *J. Comput. Chem.* **2011**, *32*, 1456–1465, DOI: https://doi.org/10.1002/jcc.21759.
(10) Weigend, F.; Ahlrichs, R. Balanced basis sets of split valence, triple zeta valence and quadruple zeta valence quality for H to Rn: Design and assessment of accuracy. *Phys. Chem. Chem. Phys.* **2005**, *7*, 3297–3305, DOI: 10.1039/B508541A.
(11) Marenich, A. V.; Cramer, C. J.; Truhlar, D. G. Universal Solvation Model Based on Solute Electron Density and on a Continuum Model of the Solvent Defined by the Bulk Dielectric Constant and Atomic Surface Tensions. *J. Phys. Chem. B* **2009**, *113*, 6378–6396, DOI: 10.1021/jp810292n.
(12) Glendening, E.D.; Badenhoop, J. K.; Reed, A. E.; Carpenter, J. E.; Bohmann, J. A.; Morales, C. M.; Karafiloglou, P.; Landis, C. R.; Weinhold, F. *NBO 7.0, Theoretical Chemistry Institute*, University of Wisconsin, Madison, WI, 2018.
(13) Hanwell, M. D.; Curtis, D. E.; Lonie, D. C.; Vandermeersch, T.; Zurek, E.; Hutchison, G. R. Avogadro: an advanced semantic chemical editor, visualization, and analysis platform. *J. Cheminformatics* **2012**, *4*, 17, DOI: 10.1186/1758-2946-4-17.
